# A *helitron*-induced RabGDIα variant causes quantitative recessive resistance to maize rough dwarf disease

**DOI:** 10.1038/s41467-020-14372-3

**Published:** 2020-01-24

**Authors:** Qingcai Liu, Suining Deng, Baoshen Liu, Yongfu Tao, Haiyue Ai, Jianju Liu, Yongzhong Zhang, Yan Zhao, Mingliang Xu

**Affiliations:** 1 State Key Laboratory of Plant Physiology and Biochemistry/College of Agronomy and Biotechnology/National Maize Improvement Center/Center for Crop Functional Genomics and Molecular Breeding, China Agricultural University, 2 West Yuanmingyuan Road, Beijing, 100193 P. R. China; 2 College of Agronomy/State Key Laboratory of Crop Biology, Shandong Agricultural University, Taian, 271018 P. R. China

**Keywords:** Agricultural genetics, Natural variation in plants, Plant domestication, Biotic

## Abstract

Maize rough dwarf disease (MRDD), caused by various species of the genus Fijivirus, threatens maize production worldwide. We previously identified a quantitative locus *qMrdd1* conferring recessive resistance to one causal species, rice black-streaked dwarf virus (RBSDV). Here, we show that Rab GDP dissociation inhibitor alpha (RabGDIα) is the host susceptibility factor for RBSDV. The viral P7-1 protein binds tightly to the exon-10 and C-terminal regions of RabGDIα to recruit it for viral infection. Insertion of a *helitron* transposon into *RabGDIα* intron 10 creates alternative splicing to replace the wild-type exon 10 with a *helitron*-derived exon 10. The resultant splicing variant RabGDIα-hel has difficulty being recruited by P7-1, thus leading to quantitative recessive resistance to MRDD. All naturally occurring resistance alleles may have arisen from a recent single *helitron* insertion event. These resistance alleles are valuable to improve maize resistance to MRDD and potentially to engineer RBSDV resistance in other crops.

## Abstract (web summary)

Maize rough dwarf disease threatens its production. Here, the authors show that a *helitron* transposon insertion in the Rab GDP dissociation inhibitor alpha leads to recessive viral resistance by affecting its interaction with viral P7-1 protein and that all naturally occurring alleles come from a single mutation event after domestication.

## Introduction

Plant viral diseases cause serious yield losses and quality reduction in major crops, threatening world food security^1,2^. Plant viruses encode only a few essential proteins and thus depend on host factors to complete their infection life cycles^3–7^. Generally, a plant activates its defense response to counterattack viral invasion, the so-called active resistance response. Alternatively, a plant modifies the target protein(s) that allows it to avoid viral recognition, leading to passive resistance^8,9^. The loci encoding these modified proteins generally act as recessive resistance genes. To date, many naturally occurring plant recessive resistance genes have been isolated and characterized, most of them related to the eukaryotic translation initiation factors eIF4E and eIF4G and their isoforms (eIFiso4E and eIFiso4G)^5,10–14^, and some are mutants of other types of genes, such as *HvPDIL5-1, cPGK2*, and *CmVPS41*^15–17^.

Maize rough dwarf disease (MRDD) poses a grave threat to maize (*Zea mays* L.) production worldwide. Since its discovery in the late 1940s in Italy, the disease has gradually become one of the most destructive diseases of maize and currently MRDD plagues all maize-growing continents around the world, causing heavy yield losses, ranging from 30 to 100%^18–21^. For instance, in recent decades, MRDD has become prevalent in the Yellow and Huai River valley, a major maize-growing area in China. In Shandong province alone, the disease led to heavy yield losses in over 733,000 hectares in 2008^22^.

**Fig. 1:**
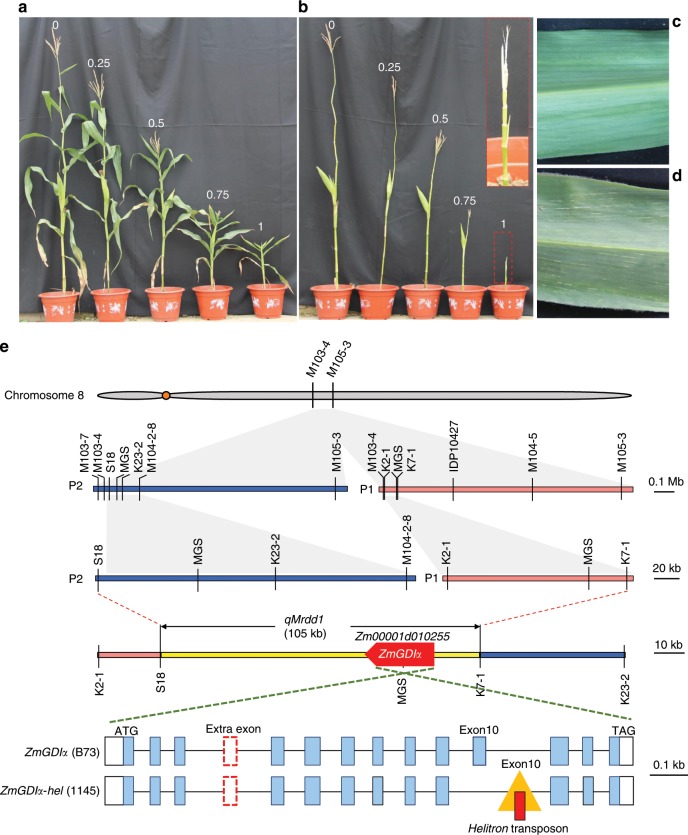
Fine-mapping of the MRDD resistance QTL *qMrdd1*. **a**, **b** The RBSDV-infected maize plants are characterized by stunted growth (**a**) and shortened internodes (**b**). The numbers in **a**/**b** indicate a five-grade scale, ranging from healthy plant (grade 0) to severely diseased plant (grade 1). **c**, **d** The absence (**c** healthy plants) or presence (**d** severely diseased plants) of waxy enations on the axial surfaces of upper leaves. **e** Fine-mapping of *qMrdd1*. The mappings with the P1 (salmon pink bar) and P2 (navy blue bar) populations, derived from crosses between the inbred lines NT409 (susceptible) and NT411 (resistant) and between HuangC (susceptible) and X178 (resistant), respectively, are depicted on the right and left, respectively. The overlapped S18/K7-1 interval (105 kb; yellow bar) is inferred to encompass the causal genetic factor for *qMrdd1*. The candidate gene *RabGDIα* (code: *Zm00001d010255*) is depicted as a red arrow. The structures of the candidate genes in the susceptible B73 (*ZmGDIα*) and resistant 1145 (*ZmGDIα-hel*) lines are shown below, with blue boxes representing exons, white boxes representing the 3′- and 5′-UTRs, and the red dashed box representing the extra exon of the long transcript. The *helitron* insertion (golden triangle) in intron 10 results in alternative splicing, which replaces the wild-type exon 10 with a *helitron*-derived exon 10 (red box within the golden triangle) to create *ZmGDIα-hel*.

The viruses that cause MRDD belong to the genus Fijivirus in the family *Reoviridae*. Viral species vary between continents. Rice black-streaked dwarf virus (RBSDV) and Southern rice black-streaked dwarf virus (SRBSDV) are mainly distributed in Asia; Maize rough dwarf virus (MRDV) was detected in Europe and Mal de Rio Cuarto virus (MRCV) was reported in South America^23–26^. A recent study indicates that MRDV and RBSDV should be classified as different geographic strains of a single species, named Cereal black-streaked dwarf Fijivirus (CBSDV), due to their high genome sequence identities^27^. These viruses are naturally transmitted by the small brown planthopper (*Laodelphax striatellus*) in a persistent propagative manner^28^. The genomes of these Fijiviruses consist of ten linear double-stranded RNA (dsRNA) segments (S1–S10), each encoding one or two (for S5, S7, and S9) proteins^18,29,30^. Of the resulting 13 viral proteins, P7-1 is a tubule-forming protein in both infected plants and insect vectors^31^. P7-1 can self-assemble into virus-containing tubules which usually appear as punctate structures at plasmodesmata, presumably to assist viral intercellular movement and dissemination^32,33^. MRDD-infected plants are usually severely stunted with dark-green leaves, shortened internodes, and waxy enations on the abaxial surfaces of upper leaves (Fig. 1a–d). When severely infected, plants may even lack tassels and ears. Given the lack of resistant maize cultivars, postponed sowing or agrochemical control of planthoppers has been widely used to contain viral infection^34^. However, these measures tend to waste light and temperature resources or cause environmental pollution, making them problematic. Identifying natural resistance genes and breeding resistant maize varieties would be an economical and environmentally friendly means to minimize the yield losses caused by MRDD. To date, researchers have put extensive efforts into unveiling the genetic basis of MRDD resistance. Maize resistance to MRDD is a typical quantitative trait, and a number of quantitative trait loci (QTL) have been identified in different chromosomal regions based on linkage mapping or genome-wide association study^35–37^. A recent study reported the fine mapping of a resistance QTL *qMrdd8* in a 347-kb interval on chromosome 8^38^. Nevertheless, no resistance gene has yet been identified, let alone any molecular mechanisms underlying resistance^21^.

This study reports the map-based cloning of maize *ZmGDIα-hel*, a causal gene for *qMrdd1*, which confers passive resistance to MRDD caused by RBSDV. The wild-type *ZmGDIα* gene encodes a Rab GDP dissociation inhibitor alpha (RabGDIα), which is required for vesicle trafficking. A *helitron* insertion in *ZmGDIα* induces alternative splicing to create a recessive resistance allele, *ZmGDIα-hel*, which reduces the MRDD disease severity index by ~30%. The viral P7-1 protein is the pathogenicity determinant that binds *ZmGDIα* on its exon-10-encoded peptide and C-terminal residues for viral infection. These findings enrich our knowledge of naturally occurring recessive resistance genes and the underlying molecular mechanisms controlling viral diseases.

## Results

### Cellular ultrastructure of MRDD symptoms

We infected two near-isogenic lines, NIL-S and NIL-R, which differ for *qMrdd1* to identify MRDD-induced cellular characteristics (Supplementary Fig. 1a). We found that in the ninth internodes, for example, cell elongation was suppressed in the diseased NIL-S as compared to the healthy NIL-R plants (Supplementary Fig. 1b–d). Moreover, we observed aberrant vascular cylinders, characterized by supernumerary phloem cells and atrophic xylem, in the diseased NIL-S plants (Supplementary Fig. 1e, f). Formation of waxy enations on the abaxial surface of upper leaves is the most distinct feature of the diseased plants (Fig. 1c, d). Astonishingly, we found that this waxy enation is actually the bulging vascular cylinder beneath the lower epidermis, which consists of massive, irregular phloem cells (Supplementary Fig. 1g, h). The vascular cylinder plays a vital role in the transport of water, inorganic salts, and organic nutrients, and thus aberrant vascular cylinders presumably hinder plant systemic transport, resulting in severely stunted plants.

### Sequential fine mapping of *qMrdd1*

We had previously mapped a major recessive resistance QTL, *qMrdd1*, in a 1.2-Mb interval of maize chromosome 8; *qMrdd1* reduces the disease severity index (DSI) of MRDD by 24.2–39.3%^37^. With the goal of cloning the causal resistance gene, we continued to resolve *qMrdd1* using two mapping populations, P1 and P2, prepared from crosses between the inbred lines NT409 (susceptible) and NT411 (resistant) and between HuangC (susceptible) and X178 (resistant), respectively. Accordingly, we developed a set of molecular markers to saturate the *qMrdd1* region for the two populations (Fig. 1e and Supplementary Data 1).

Based on the recombinant-derived progeny-testing strategy^39^, we identified 11 BC_1_F_5_ (backcrossed once, then selfed five times) recombinants from the P1 population in 2013, which we then selfed to produce BC_1_F_6_ progeny. Each BC_1_F_6_ plant was investigated for its genotype and MRDD resistance in a field test. Within the BC_1_F_6_ progeny, we estimated DSI values for the three genotypes (NT411/NT411, NT411/NT409, and NT409/NT409) at the *qMrdd1* region. A significant difference in DSI between the two homozygous genotypes indicated the presence of a heterozygous *qMrdd1* locus in their parental BC_1_F_5_ recombinant, or otherwise a homozygous or no *qMrdd1* locus. Analysis of 11 recombinants enabled us to restrict the location of *qMrdd1* to an interval flanked by the markers M103-4 and IDP-7 (Supplementary Fig. 2a). Over the next 3 years, we obtained three further recombinants, which allowed us to refine the right-flanking marker from IDP-7 to K7-1 (Supplementary Fig. 2b). In 2017, with four more recombinants, *qMrdd1* was restricted to the K2-1/K7-1 interval (Supplementary Fig. 2c). On average, *qMrdd1* homozygotes showed significantly reduced DSI by 17.7–34.3% in 2013, 20.5–31.9% in 2016, and 29.2–40.0% in 2017, respectively, as compared with non-*qMrdd1* homozygotes (Supplementary Fig. 2d). The lack of a significant difference in DSI between heterozygotes and non-*qMrdd1* homozygotes demonstrated that *qMrdd1* acts in a recessive genetic mode in MRDD resistance (Supplementary Fig. 2d).

Taking the same approach, we obtained four recombinants from the P2 population in 2013. Progeny testing allowed us to narrow down the location of *qMrdd1* to a region between markers M103-7 and M104-2-8 (Supplementary Fig. 3a). During 2014–2016, we obtained seven recombinants in the M103-7/M104-2-8 interval, which enabled us to map *qMrdd1* to a region flanked by markers S18 and M104-2-8 (Supplementary Fig. 3b). In 2017, progeny testing with eight more recombinants confirmed the mapped interval of S18/M104-2-8 (Supplementary Fig. 3c). Similarly, *qMrdd1* homozygotes showed DSI that was reduced by 19.2–27.9% in 2016 and 16.1–41.4% in 2017 (Supplementary Fig. 3d). The two mapped *qMrdd1* regions, K2-1/K7-1 from P1 and S18/M104-2-8 from P2, overlapped with each other, and we inferred that the 105-kb overlapping region flanked by markers K7-1 and S18 encompasses the causal genetic locus for *qMrdd1* (Fig. 1e).

### A gene encoding *RabGDIα* is the candidate for MRDD resistance

The markers in the mapped *qMrdd1* region were used to screen two bacterial artificial chromosome (BAC) libraries constructed from the resistant (1145) and susceptible (HZ4) inbred lines. Two overlapping 1145 BAC clones covering the 105-kb *qMrdd1* region and a *qMrdd1*-tagged HZ4 clone were subjected to sequencing and gene annotation (Supplementary Fig. 4a). Alignment of predicted genes among 1145, HZ4, and B73 (susceptible line) revealed five non-transposon-related genes in the *qMrdd*1 region, encoding, respectively, an invertase inhibitor, glutathione S-transferase T3-like, ALP1-like, a hypothetical protein, and Rab GDP dissociation inhibitor alpha (RabGDIα). For the first four predicted genes, no sequence variation was present between 1145 and B73 (Supplementary Fig. 4a). Moreover, these four putative genes showed no gene expression in any of the five lines tested (Supplementary Fig. 4b), and thus could be excluded as candidates for causing MRDD resistance. By contrast, the fifth gene *RabGDIα* showed distinct sequence divergence between resistant and susceptible lines, and we found that it contained a 2548-bp *helitron* transposable element (TE) in the resistant line 1145, but not in the susceptible lines HZ4 and B73 (Fig. 1e and Supplementary Fig. 4a). Intriguingly, *RabGDIα* had very similar gene expression levels between resistant and susceptible lines (Supplementary Fig. 4b). Taken together, these results pointed to *RabGDIα* as the candidate for *qMrdd1*, henceforth named *ZmGDIα* for the wild-type allele and *ZmGDIα-hel* for the *helitron*-inserted allele (Fig. 1e and Supplementary Data 2).

We obtained the full-length *ZmGDIα* and *ZmGDIα-hel* cDNAs (Supplementary Fig. 4c), and compared them to the corresponding genomic DNAs. The *ZmGDIα* and *ZmGDIα-hel* alleles each have two transcript isoforms, with the long transcripts, consisting of 14 exons, having an extra exon after exon 3 as compared with the short transcripts (13 exons). For both isoforms, the *ZmGDIα-hel* transcript differs from the *ZmGDIα* transcript in having an alternative exon 10, which is transcribed from the *helitron* TE rather than the original *ZmGDIα* sequence (Fig. 1e and Supplementary Data 3). Thus, the *helitron* insertion causes alternative splicing that creates mutually exclusive versions of exon 10 in the *ZmGDIα* and *ZmGDIα-hel* transcripts.

We collected 24 Rab GDP dissociation inhibitor protein sequences from various plant species in the NCBI database (http://www.ncbi.nlm.nih.gov) and conducted alignment and phylogenetic analyses of the different RabGDIα proteins. The deduced amino acid sequences for the extra exon in the long *ZmGDIα* and *ZmGDIα-hel* transcripts seem to be unique to maize (Supplementary Fig. 5). A short stretch of 27 amino-acid residues encoded by the alternative exon 10 is strictly confined to the ZmGDIα-hel protein (Supplementary Fig. 5). Among the other proteins we analyzed, maize RabGDIα has the highest sequence similarity with the sorghum protein Sb09g020530 (Supplementary Fig. 6).

### Validation of *ZmGDIα* and *ZmGDIα-hel* in resistance to MRDD

With the aim of identifying the relevant gene at the *qMrdd1* locus, we made seven constructs for functional verification of *ZmGDIα*/*ZmGDIα-hel*, including one complementation construct containing the native *ZmGDIα* gene, four overexpression constructs corresponding to the long and short full-size cDNAs from both *ZmGDIα* and *ZmGDIα-hel*, and two RNA interference (RNAi) constructs.

**Fig. 2:**
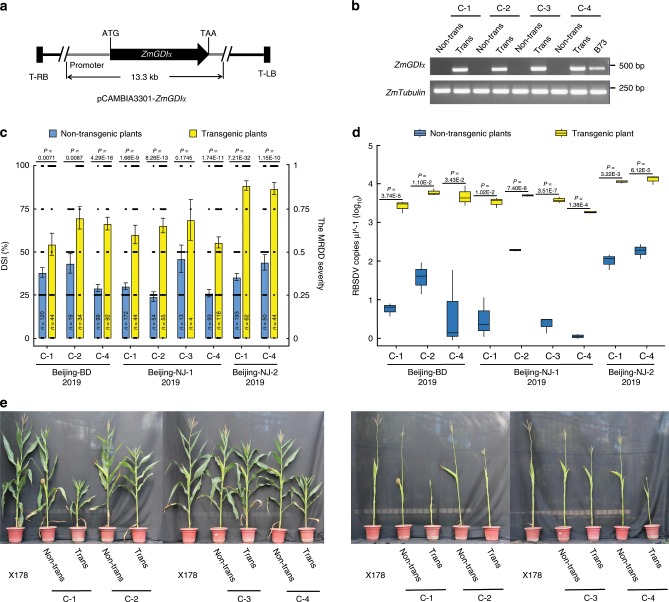
Functional validation of *ZmGDIα* via a transgenic approach. **a** Structure of pCAMBIA3301-*ZmGDIα* used for the complementation assay. T-RB and T-LB: right and left T-DNA borders, respectively. **b** Validation of transgenic plants by RT-PCR. The PCR products were observed in T_2_F_1_BC_2_ transgenic plants (Trans) but not in non-transgenic siblings (Non-trans). The maize gene *ZmTubulin* was used as an internal control. The experiment was repeated three times independently, with the similar results. **c** Disease severity index (DSI) values of T_2_F_1_BC_2_ plants. The viruliferous planthoppers reared in Baoding (BD) and Nanjing (NJ-1, NJ-2) were used for artificial inoculation in Beijing. DSI values were estimated for both transgenic and non-transgenic T_2_F_1_BC_2_ plants and are denoted as mean ± SEM. The number of plants is indicated in each column. Each dot represents the disease severity of a single plant. **d** The mean RBSDV copies µl^-1 (log_10_) and distribution in T_2_F_1_BC_2_ plants. Leaf tissues were sampled three times independently as three biological replicates. Box edges represent quartiles, and the medians were shown with the central line in the boxes. **e** The resistance performance of T_2_F_1_BC_2_ progeny plants. Transgenic plants (Trans) were more susceptible to MRDD than non-transgenic siblings (Non-trans) with stunting plants, shortened internodes, and malformed tassels/ears. The inbred line X178 was used as a resistant control. *P*-values were estimated by two-tailed Student’s *t*-test. Source data underlying **b**–**e** are provided as a Source Data file.

We transformed the complementation construct (pCAMBIA3301-*ZmGDIα*) into the susceptible recipient line B73. Four independent transgenic events were obtained, expressing the exogenous *ZmGDIα* gene (Fig. 2a, b). We crossed T_2_ transgenic plants with the resistant line X178 and backcrossed twice to X178 to obtain T_2_F_1_BC_2_ progeny. The T_2_F_1_BC_2_ plants that had the endogenous *ZmGDIα-hel* allele fixed were artificially inoculated with RBSDV at the seedling stage, and examined their RBSDV copy numbers in the top leaves at 58 days post inoculation (dpi) and resistance performance at 90 dpi. Notably, transgenic plants expressing the exogenous native gene *ZmGDIα* had significantly higher DSI values (except for one transgenic event C-3) and RBSDV copy numbers than did non-transgenic plants (Fig. 2c–e).

With the overexpression construct containing the short *ZmGDIα* cDNA (*pUbi:*:*ZmGDIα-EGFP*), two independent transgenic events were obtained, as shown by gene expression and immunoblotting assays (Supplementary Fig. 7a–c). Similarly, transgenic T_2_F_1_BC_2_ plants overexpressing the exogenous short *ZmGDIα* cDNA had significantly higher DSI values and RBSDV copy numbers (except for one transgenic event SS-2 in 2018) than did non-transgenic plants (Supplementary Fig. 7d–f). By contrast, overexpression of the long *ZmGDIα* cDNA (*pUbi:*:*ZmGDIα*^*L*^*-EGFP*) did not show any significant differences in DSI values between transgenic and non-transgenic plants (Supplementary Fig. 8). Taken together, it is obvious that complementation of *ZmGDIα-hel* with the native *ZmGDIα* gene or short *ZmGDIα* cDNA could largely restore the ability of RBSDV to systemically infect maize plants.

We also transformed two *ZmGDIα-hel* overexpression constructs, *pUbi:*:*ZmGDIα-hel-EGFP* and *pUbi:*:*ZmGDIα-hel*^*L*^*-EGFP*, into the recipient line B73, and yielded two and three independent transgenic events, respectively. Just like their susceptible B73 progenitor, all transgenic T_4_ lines overexpressing either short or long exogenous *ZmGDIα-hel* cDNA were all severely infected by RBSDV (Supplementary Fig. 9), indicating that *ZmGDIα-hel* could not enhance resistance to MRDD if the endogenous *ZmGDIα* allele is present. This further demonstrates that *ZmGDIα-hel* is a recessive resistance gene to MRDD.

Next, we transformed two RNA interference constructs (*pUbi:*:*ZmGDIα*^*230*^*-RNAi* and *pUbi:*:*ZmGDIα*^*196*^*-RNAi*) into the recipient B73, generating nine independent transgenic events in total. Gene expression levels of *ZmGDIα* were reduced by 33–68% in the transgenic plants compared to B73. Regardless of the reduced *ZmGDIα* expression, all transgenic plants were highly susceptible to MRDD, the same as their susceptible B73 progenitor (Supplementary Fig. 10). We speculated that even relatively low expression of the endogenous *ZmGDIα* is enough for RBSDV infection.

These assessments demonstrated that wild-type *ZmGDIα* is a dominant susceptibility allele for RBSDV infection, while the natural *ZmGDIα-hel* variant is a recessive resistance allele. Therefore, the polymorphic *helitron* TE insertion is the causal genetic variant that distinguishes *ZmGDIα* from *ZmGDIα-hel*.

### Molecular characterization of *ZmGDIα* and *ZmGDIα-hel*

**Fig. 3:**
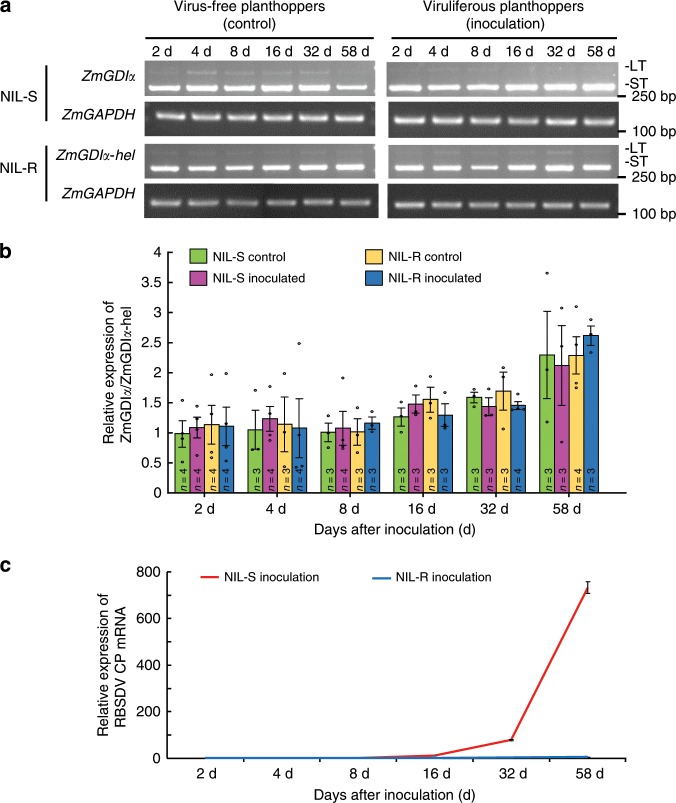
Expression profiles of *ZmGDIα*/*ZmGDIα-hel* and RBSDV accumulation. **a** The dynamic changes of two transcripts during viral infection. The long and short transcripts were simultaneously detected by RT-PCR at 2, 4, 8, 16, 32, and 58 days after inoculation. *ZmGAPDH* was used as an internal control. LT long transcript, ST short transcript. **b** Relative expression levels in *ZmGDIα* and *ZmGDIα-hel*. The values are denoted as mean ± SEM. Three or four samples were taken as biological replicates (*n*). Each dot indicates the expression level of a single biological replicate. **c** The relative expression of RBSDV coat protein (CP) mRNA. The number of samples is consistent with that in “**b**”. The values are denoted as mean ± SEM. Red line: inoculated NIL-S; blue line: inoculated NIL-R. Each of above three experiments was repeated three times independently, with the similar results. Source data are provided as a Source Data file.

We used NIL-S and NIL-R to investigate the expression of *ZmGDIα* and *ZmGDIα-hel* during viral infection. We inoculated maize seedlings with RBSDV at the two-leaf stage. The long and short transcripts were simultaneously assayed by reverse transcription PCR (RT-PCR) at 2, 4, 8, 16, 32, and 58 dpi. For both *ZmGDIα* and *ZmGDIα-hel*, the short transcript was much more abundant than the long transcript, and this biased expression profile was not influenced by either RBSDV infection or developmental stage (Fig. 3a). Because of this, we performed RT-qPCR assays to quantify the total number of transcripts. At each assay point, gene expression levels were comparable between *ZmGDIα* and *ZmGDIα-hel* under either infected or non-infected conditions. Moreover, transcript abundance showed no detectable change at the early infection stages and increased slightly after 16 dpi (Fig. 3b). Accumulation of RBSDV was detected only in NIL-S, and the quantity of RBSDV remained very low in the early stages of viral infection up until 16 dpi, and then rose rapidly to reach high level at 58 dpi (Fig. 3c). The *ZmGDIα* expression profile was almost unresponsive to either *helitron* insertion or RBSDV accumulation. Thus, we infer that it is the amino acid sequence rather than the gene expression level that determines maize resistance to MRDD.

### P7-1 binds strongly to RabGDIα but weakly to RabGDIα-hel

Having identified the host MRDD susceptibility factor as RabGDIα, we were eager to know which of the 13 RBSDV proteins recruits RabGDIα during viral infection. We therefore inoculated transgenic plants overexpressing the *ZmGDIα-EGFP* fusion gene with RBSDV at the two-leaf stage. Based on the RBSDV accumulation curve, we collected infected plants at 11, 13, 19, and 28 dpi for immunoprecipitation of the ZmGDIα-EGFP fusion protein with anti-GFP antibody, followed by mass spectrometry analysis (Supplementary Fig. 11a). Although numerous maize proteins were identified in this analysis, no viral proteins were immunoprecipitated until 19 dpi, when three RBSDV proteins (P7-1, P8, and P10) were detected (Supplementary Data 4).

**Fig. 4:**
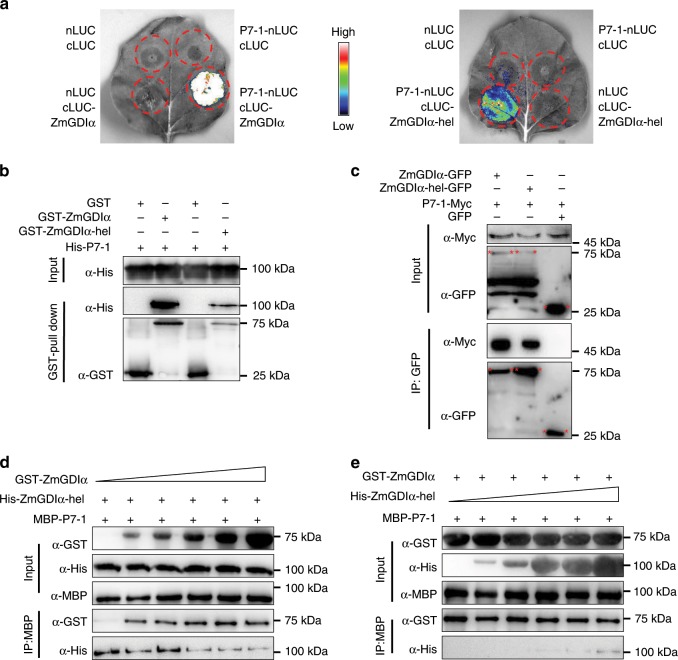
The viral P7-1 protein binds more tightly to ZmGDIα than to ZmGDIα-hel. **a** Split-luciferase complementation assay to show the interaction of viral P7-1 with ZmGDIα or ZmGDIα-hel. Fluorescence signals appeared where P7-1-nLUC was co-expressed with cLUC-ZmGDIα (left) or cLUC-ZmGDIα-hel (right) in *N. benthamiana* leaves. The high-low reference bar shows fluorescence signals, ranging from high (top, white with red) to low (bottom, blue). **b** GST pull-down assay to show the interactions of both ZmGDIα and ZmGDIα-hel with viral P7-1 in vitro. Pull-down of the His-P7-1 fusion protein is detected by immunoblotting with an anti-His antibody. **c** Co-immunoprecipitation assay to show the interaction of viral P7-1 with ZmGDIα or mGDIα-hel in vivo. P7-1-Myc and ZmGDIα-EGFP (or ZmGDIα-hel-EGFP) were detected by immunoblotting with anti-Myc and anti-GFP antibody, respectively. **d** Competitive protein-binding assay with a fixed amount of His-ZmGDIα-hel (200 μg). The interacting proteins were pulled down with anti-MBP amylose resin and detected by immunoblotting with either GST (for GST-ZmGDIα) or His (for His-ZmGDIα-hel) antibody. **e** Competitive protein-binding assay with a fixed amount of GST-ZmGDIα (200 μg). In the inverse experiment to that in **d**, the interacting proteins were pulled down with anti-MBP amylose resin and detected by immunoblotting with either GST (for GST-ZmGDIα) or His (for His-ZmGDIα-hel) antibody. Each of above five experiments was repeated three or more times independently, with the similar results. Source data are provided as a Source Data file.

To verify which viral protein interacts with RabGDIα, we conducted multiple assays for virus–host protein interactions. In a split-luciferase complementation assay, each of three viral fusion genes (*S7-1-nLUC*, *S8-nLUC*, and *S10-nLUC*) was co-expressed with *cLUC-ZmGDIα* or *cLUC-ZmGDIα-hel* in *N. benthamiana* leaves. Only P7-1-nLUC strongly interacted with cLUC-ZmGDIα, while neither P8-nLUC nor P10-nLUC showed any interaction with cLUC-ZmGDIα (Supplementary Fig. 11b). Similarly, P7-1-nLUC also interacted with cLUC-ZmGDIα-hel (Fig. 4a). In vitro pull-down assays confirmed the interactions of ZmGDIα and ZmGDIα-hel with P7-1 (Fig. 4b); by contrast, ZmGDIα did not pull down either P8 or P10 (Supplementary Fig. 11c). P7-1 was further shown to interact with both ZmGDIα and ZmGDIα-hel using an in vivo co-immunoprecipitation assay (Fig. 4c). To explore the differences between ZmGDIα and ZmGDIα-hel in regard to interaction with P7-1, we performed a competitive protein-binding assay. As shown, ZmGDIα could gradually replace ZmGDIα-hel in interacting with P7-1 as the amount of ZmGDIα increased (Fig. 4d); however, ZmGDIα-hel was much less effective at displacing ZmGDIα in the inverse assay (Fig. 4e). It is obvious that the viral P7-1 protein binds more tightly to ZmGDIα than to ZmGDIα-hel.

### Helitron insertion weakens the binding of P7-1 to ZmGDIα-hel

Given that helitron insertion creates an alternative exon 10 in *ZmGDIα-hel* cDNA, we infer that the variations in the amino-acid residues encoded by the different exon 10 variants might underlie the difference between the interactions of ZmGDIα and ZmGDIα-hel with P7-1. The wild-type RabGDIα (ZmGDIα) and splicing mutant RabGDIα-hel (ZmGDIα*-*hel) consist of 445 and 430 amino-acid residues, respectively (Supplementary Fig. 12a). Protein homology modeling indicated that *ZmGDIα* exon 10 encodes 42 amino-acid residues that form two β sheets, whereas *ZmGDIα-hel* exon 10 encoded 27 residues in which the β-sheets are almost entirely lacking (Supplementary Fig. 12b, c).

**Fig. 5:**
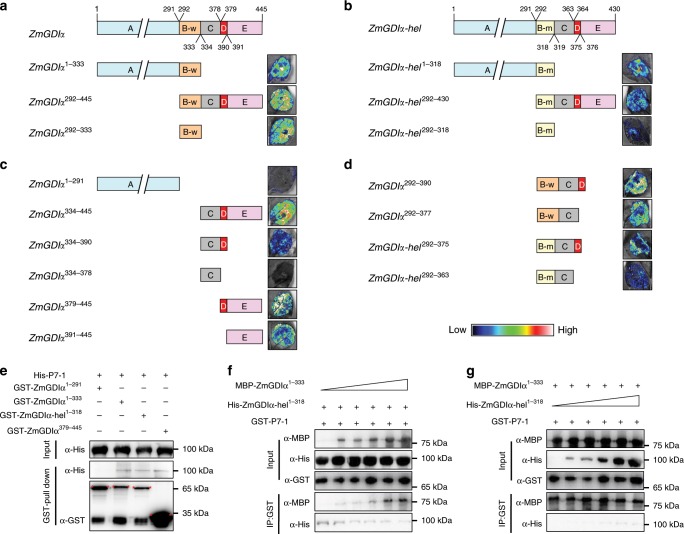
Searching for viral P7-1-binding regions in ZmGDIα and ZmGDIα-hel. To reveal the P7-1-binding regions, *ZmGDIα* was divided into multiple segments, marked with different colors (the numerals above/under segments are amino-acid numbers counted starting from the initiation codon): A: *ZmGDIα*^1–291^, light green rectangle; B-w: *ZmGDIα*^*292–333*^, orange rectangle; C: *ZmGDIα*^*334–378*^, gray rectangle; D: *ZmGDIα*^*379–390*^, red rectangle; E: *ZmGDIα*^*391–445*^, pink rectangle. The B-w counterpart in *ZmGDIα-hel* is denoted as B-m: *ZmGDIα-hel*^*292–318*^, light yellow rectangle. Images at right are from split luciferase complementation assays in which various *cLUC*-tagged *ZmGDIα* or *ZmGDIα-hel* gene segments were co-expressed with the *nLUC*-tagged viral *S7-1* in *N. benthamiana* leaves. **a** Viral P7-1 interacted with the exon-10-encoded residues in ZmGDIα. **b** Viral P7-1 also interacted with the *helitron*-derived exon-10-encoded residues in ZmGDIα-hel. **c** Viral P7-1 bound to theC-terminal 67 residues. **d** The ZmGDIα^334–378^ fragment has no influence on its flanking fragments regarding to their interactions with P7-1. The high-low reference bar shows fluorescence signals, ranging from high (left, white with red) to low (right, blue). **e** GST pull-down assay in vitro. Pull-down of the His-P7-1 fusion protein is detected for ZmGDIα^1–333^, ZmGDIα-hel^1–318^, and ZmGDIα^379–445^, but not ZmGDIα^1–291^, by immunoblotting with an anti-His antibody. **f** Competitive protein-binding assay with a fixed amount of His-ZmGDIα-hel^1–318^ (200 μg). The interacting proteins were pulled down with anti-GST agarose resin and detected by immunoblotting with either MBP (for MBP-ZmGDIα^1–333^) or His (for His-ZmGDIα-hel^1–318^) antibody. **g** Competitive protein-binding assay with a fixed amount of MBP-ZmGDIα^1–333^ (200 μg). The interacting proteins were pulled down with anti-GST agarose resin and detected by immunoblotting with either MBP (for MBP-ZmGDIα^1–333^) or His (for His-ZmGDIα-hel^1–318^) antibody. Each experiment was repeated three or more times independently, with the similar results. Source data are provided as a Source Data file.

We divided *ZmGDIα* and *ZmGDIα-hel* cDNAs (short isoforms) into multiple segments to help identify the P7-1-binding regions. In a split-luciferase complementation assay, all three exon-10-containing ZmGDIα segments (ZmGDIα^1–333^, ZmGDIα^292–445^, and ZmGDIα^292–333^) strongly interacted with P7-1, indicating that the exon-10-encoded peptide plays a key role in the interaction with ZmGDIα (Fig. 5a). For the three ZmGDIα-hel counterparts (ZmGDIα-hel^1–318^, ZmGDIα-hel^292–430^, and ZmGDIα-hel^292–318^), we also detected their interactions with P7-1, implying that the residues encoded by the *helitron*-derived exon 10 did not completely abolish its interaction with P7-1 (Fig. 5b).

Next, we focused on ZmGDIα to determine whether any other regions might assist ZmGDIα in interacting with P7-1. The N-terminus upstream of exon 10 (ZmGDIα^1–291^) did not show any interaction with P7-1 in assays, but the C-terminus downstream of exon 10 (ZmGDIα^334–445^) did show an interaction with P7-1 (Fig. 5c). Given that there is a structural variation in the β18 sheet between ZmGDIα and ZmGDIα-hel (Supplementary Fig. 12), we divided *ZmGDIα*^334–445^ into four segments, two with (*ZmGDIα*^334–390^ and *ZmGDIα*^379–445^) and two without (*ZmGDIα*^334–378^ and *ZmGDIα*^391–445^) the β18 sheet. Only one segment, *ZmGDIα*^334–378^, did not show a luciferase signal when co-expressed with *S7-1* (Fig. 5c). These facts demonstrated that the β18 sheet and the rest of C-terminus still interacted with P7-1. Furthermore, when the four *ZmGDIα*^334–378^-containing segments, *ZmGDIα*^292–390^, *ZmGDIα*^292–378^, *ZmGDIα-hel*^292–375^, and *ZmGDIα-hel*^292–363^, were individually co-expressed with *S7-1*, luciferase signals reappeared (Fig. 5d). This suggests that ZmGDIα^334–378^ has no influence on its flanking fragments in regard to their interactions with P7-1.

We then selected four fragments, ZmGDIα^1–291^, ZmGDIα^1–333^, ZmGDIα-hel^1–318^, and ZmGDIα^379–445^, to use in confirming the above interaction results by pull-down assay. Apart from ZmGDIα^1–291^, the other three segments could interact with P7-1 (Fig. 5e). This confirms that the exon-10-encoded peptide and C-terminal 67 residues are both P7-1-binding regions. We further used ZmGDIα^1–333^ and ZmGDIα-hel^1–318^ to conduct a competitive protein-binding assay to scrutinize the differences between the exon-10-encoded peptides of ZmGDIα and ZmGDIα-hel in their interaction with P7-1. The results showed that P7-1 bound more tightly to ZmGDIα^1–333^ than to ZmGDIα-hel^1–318^ (Fig. 5f, g). Naturally, substitution of a *helitron*-derived exon 10 for the wild-type exon 10 greatly reduces the efficiency of P7-1 in recruiting ZmGDIα-hel.

### Origin of the recessive resistance allele *ZmGDIα-hel*

We surveyed a large collection of diverse maize inbred lines (620), maize landraces (336), and teosintes (184), and found only 36 lines (34 inbred lines and 2 landraces) with a *helitron* TE in *ZmGDIα* (Supplementary Data 5–7). We re-sequenced the intact *helitron* TEs and their adjacent regions in these 36 lines and found that all of the *helitron* TEs are inserted into the same site in *ZmGDIα* and have no sequence divergence. We further selected a set of 24 *ZmGDIα-hel* maize lines, 25 *ZmGDIα* maize lines, and 19 teosinte entries and re-sequenced their intact *ZmGDIα-hel* or *ZmGDIα* alleles (Supplementary Data 5–7). Together with the polymorphic *helitron* TEs, 24 SNPs were found in the promoter, gene coding, and 3′ downstream regions, resulting in 20 haplotypes (Supplementary Fig. 13). Intriguingly, we did not find any nucleotide diversity of these regions among 24 *ZmGDIα-hel* alleles (Supplementary Fig. 14a). Moreover, HKA tests did not detect significant signal (*P* = 0.36) of past selection on *ZmGDIα/ZmGDIα-hel* (Supplementary Fig. 14a and Supplementary Table 1). The minimum-spanning tree analysis resulted in two main clusters: cluster I, containing all teosinte entries and *ZmGDIα* maize lines; and cluster II, composed entirely of *ZmGDIα-hel* maize lines (Supplementary Fig. 14b). These findings suggest that all natural *ZmGDIα-hel* alleles may have originated from a single *helitron* insertion event that occurred recently, well after maize domestication.

### Naturally occurring *ZmGDIα-hel* allele reduces MRDD severity

We selected 33 *ZmGDIα-hel* and 153 *ZmGDIα* maize lines to evaluate their resistance to MRDD for 3 years (2013–2015) in various locations under natural infection conditions. Regardless of the varying incidence rates of MRDD across different field trails, the *ZmGDIα-hel* maize lines consistently showed significantly lower DSI than the *ZmGDIα* maize lines (Supplementary Fig. 15a and Supplementary Data 8). Furthermore, we selected two *ZmGDIα-hel* (P138 and X178) and three *ZmGDIα* (JH59, Qi319, and Dan3130) inbred lines from which to prepare three F_2_ populations (P138 × JH59, P138 × Qi319, and X178 × Dan3130). Each F_2_ plant was investigated for its polymorphic *helitron* TE and MRDD resistance. The DSI values were significantly lower in the *ZmGDIα-hel* homozygous plants than in the *ZmGDIα* homozygous and heterozygous plants (Supplementary Fig. 15b).

Based on the polymorphic *helitron* TE, we developed three primers: one forward primer specific to the left-flanking region, and two separate reverse primers specific to the right-flanking region and *helitron* TE (Supplementary Data 1). The mixed triple primers can be easily used to differentiate *ZmGDIα* homozygous, heterozygous, and *ZmGDIα-hel* homozygous plants in any segregating populations and thus are very useful for marker-assisted introgression of the resistance allele *ZmGDIα-hel* (Supplementary Fig. 16a, b). We used a marker-assisted backcrossing strategy to introduce *ZmGDIα-hel* from the donor 1145 into numerous elite inbred lines. The converted lines showed dramatically enhanced MRDD resistance, for instance, in Chang7-2 versus converted Chang7-2 (Supplementary Fig. 16c), confirming the great value of the *ZmGDIα-hel* allele in protecting maize from attack by RBSDV.

## Discussion

RabGDIα is one of the two conserved Rab GDP dissociation inhibitors essential for cell vesicle trafficking by recycling of Rab proteins from the target back to the donor membranes^40^. RabGDIαinhibits mice defense against the intracellular vacuolar parasite *Toxoplasma gondii* by negatively regulating the Gbp2–Irga6 axis of IFN-γ-dependent cell-autonomous immunity^41^. Moreover, a 126-kDa replication protein of Tobacco mosaic virus interacts with GDI2 of plant species (*Arabidopsis*, *N. tabacum*, and *N. benthamiana*) to alter host vesicle trafficking and thereby enhance the establishment of infection^42^.

**Fig. 6:**
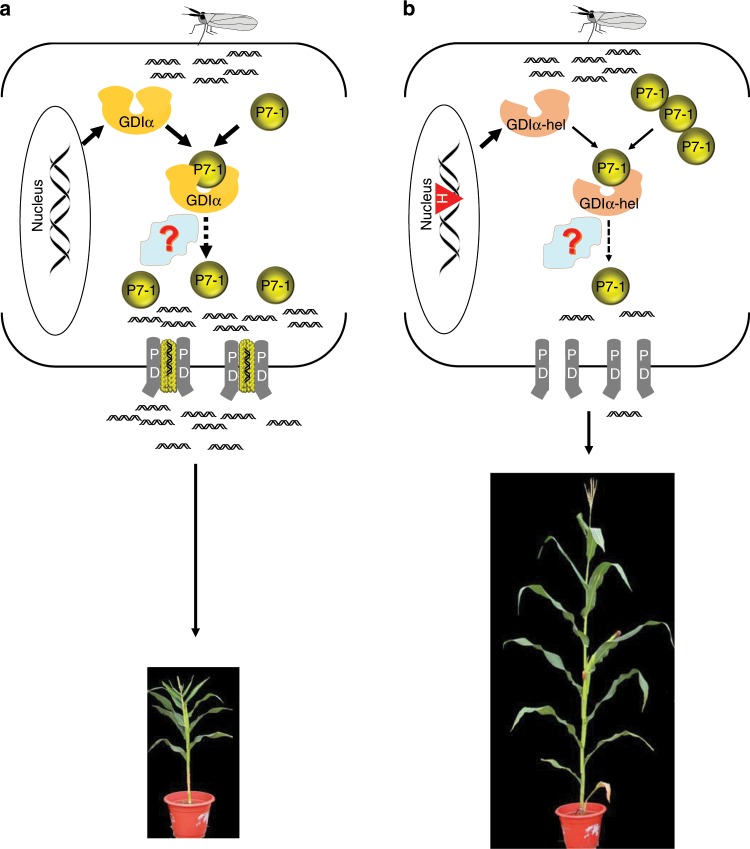
A working model of the RabGDI/RabGDI-hel in maize resistance to RBSDV. There was a quantitative difference in binding of the viral P7-1 proteins by the two protein isoforms RabGDI and RabGDI-hel, which is associated with the quantitative resistance to RBSDV. **a** In wild-type maize, viral P7-1 protein (olive circle) recruits host RabGDIα (gold horseshoe) to form a potential trafficking complex for plasmodesmata (PD, gray) targeting. P7-1 proteins form virus-containing tubules to assist viral intercellular movement and dissemination, resulting in maize susceptible to RBSDV. Black double helix: viral dsRNA. **b** Viral P7-1 protein has difficulty at recruiting RabGDIα-hel (lightsalmon horseshoe) due to its weak binding to the *helitron-*induced exon 10 peptide, and this compromises PD targeting and viral intercellular movement and dissemination, ultimately leading to quantitative recessive resistance to RBSDV. Red triangle with H indicates *helitron* transposon.

It seemed conceivable that certain RBSDV protein(s) need to hijack the host susceptibility factor RabGDIα in order for viral infection to occur. On the basis of this assumption, we used an immunoprecipitation and mass spectrometry assay to identify three viral proteins, among which only P7-1 showed tight binding to RabGDIα. Further investigation showed that P7-1 binds tightly to the exon-10-encoded peptide and C-terminal 67 residues of RabGDIα in maize. Of 13 viral genes, *S7-1* stands out as the only gene highly expressed at the earliest time point of viral infection^43^. P7-1 forms virus-containing tubules at plasmodesmata, presumably to assist viral intercellular movement and symptom development^32,33^. The P7-1 TM1 domain and its adjacent residues are required for plasmodesmata targeting, and this targeting depends on the host secretory pathway and actomyosin motility system^33^. Taken together, we speculate that P7-1 must serve as the viral pathogenicity determinant to recruit RabGDIα to form a potential trafficking complex (which may also require other cellular factors, like various Rab proteins) for viral plasmodesmata targeting, cell-to-cell movement, and dissemination (Fig. 6a). Thus, it is natural for RBSDV to produce abundant P7-1 to recruit RabGDIα for successful viral infection^43^. Coincidentally, RBSDV-induced damage of the host transport system results in the MRDD syndrome, such as severe plant stunting, shortened internodes, malformed tassels, ears, and among others.

Naturally occurring recessive resistance gene(s) generally result from amino-acid alteration or deletion of the susceptibility factor, thus impairing the virus–host interaction required for viral infection. Such resistance is likely to be passive and act in a recessive genetic mode. Insertion of a *helitron* TE into *ZmGDIα* creates a recessive resistance allele, *ZmGDIα-hel*, by inducing an alternative splicing that replaces the original exon 10 with a *helitron*-derived exon 10. So far, *helitron*-induced alternative splicing has been reported in several plant species^44,45^. A distinct feature in the current study is that the *helitron* insertion replaces only exon 10, while leaving the remainder of the gene unchanged. Perhaps the *helitron* insertion into *ZmGDI* interferes with recognition of the wild-type splice sites and introduces preferential splice sites within the *helitron* sequence.

Instead of the wild-type exon-10-encoded peptide, the newly arisen RabGDIα-hel has a short stretch of 27 amino-acid residues to which P7-1 shows weak binding affinity (Fig. 5f, g). In this scenario, P7-1 depends predominantly on the C-terminal residues to bind RabGDIα-hel; this makes P7-1 less able to recruit RabGDIα-hel, as indicated by the competitive protein-binding assay (Fig. 4d, e). The resultant low efficiency of assembly of the P7-1/RabGDIα-hel trafficking complex presumably confines RBSDV to the infected cells and thus limiting the viral movement within a plant (Fig. 6b). At a high dose of RBSDV, however, it remains possible for the resulting abundant P7-1 to recruit RabGDIα-hel to partially achieve the viral movement with a plant. Because of this, *ZmGDIα-hel* does not completely prevent RBSDV infection, but it confers quantitative recessive resistance to MRDD (Fig. 6b). So far, only one other recessive gene in the plant kingdom, encoding a melon vacuolar protein sorting 41 mutant, has been reported to prevent systemic infection, in that case by Cucumber mosaic virus^17^. Comparatively, we here revealed a naturally occurring recessive gene *ZmGDIα-hel* that directly targets viral transport to confer passive resistance to MRDD.

Although *ZmGDIα-hel* carries a *helitron* TE and has an altered exon 10, it displays the same gene expression profiles as *ZmGDIα* during RBSDV infection and at different developmental stages. Moreover, no deleterious phenotypes have been observed in maize carrying the fixed *ZmGDIα-hel* alleles^46^. Given that RabGDIα is indispensable for plant viability, it is speculated that either the RabGDIα exon 10 peptide is essential for virus–host interaction, but not for host vesicle trafficking and other functions, or the 27 residues encoded by the *helitron*-derived exon 10 behave the same as the wild-type exon 10 from the vesicle-trafficking perspective; or the loss of function could be compensated for by a functionally redundant Rab GDP dissociation inhibitor beta. We are fascinated by this *helitron*-induced alternative splicing event, and it seems less likely that an artificially mutagenized *ZmGDIα*, created for instance through CRISPR/Cas9 editing, would be able to mitigate the effects of MRDD without deleterious effects on plant growth. Furthermore, it may be possible to modify critical amino acids in the C-terminal residues of ZmGDIα-hel to create another version of *ZmGDIα-hel* that could completely evade recruitment by the viral P7-1 effector.

The identical *helitron* sequences and insertion sites suggest that all naturally occurring *ZmGDIα-hel* alleles may derive from a single *helitron* insertion event that occurred recently, well after maize domestication. It is surprising that such a resistance gene has not yet spread widely through maize germplasm. Two facts may be relevant to this puzzle. First, MRDD has only recently become a prevalent viral disease of maize, meaning that MRDD did not impose a serious threat on maize until recently. Therefore, other types of resistance genes may have emerged during maize domestication, but not been preserved due to the lack of sufficient selective pressure from MRDD. Second, unlike other major resistance gene(s), *ZmGDIα-hel* is a quantitative resistance gene and only reduces DSI by 30%, which may diminish its selective advantage and prevent it from spreading rapidly.

In maize, hundreds of families of TEs and repetitive sequences account for nearly 85% of the genome^47,48^. TE insertions are widely associated with the creation of mutant genes related to maize morphological or stress-tolerance traits^49,50^. For instance, a *Hopscotch* transposon is the functional variant at *teosinte branched1* (*tb1*), a gene related to apical dominance^51^. A *CACTA* transposon in *ZmCCT* attenuates photoperiod sensitivity and compromises resistance to stalk rot^52,53^. A *Harbinger-like* element inhibits *ZmCCT9* expression to promote flowering under long-day conditions^54^. It is also true that TE insertion is a major force creating resistance genes, as demonstrated in the current study. Since maize diseases are prevalent in tropical areas, tropical maize may be more likely to gain and retain various types of resistance genes to cope with pathogen stresses.

Given that RBSDV can be transmitted to rice, barley, and wheat in a persistent propagative manner by the small brown planthopper^55^, identification of the recessive resistance gene *ZmGDIα-hel* in maize provides a valuable tool to allow screening for similar resistance genes in other cereal crops and/or the creation of an artificial recessive gene via genome editing. Moreover, the discovery of the host susceptibility factor RabGDIα targeted by viral P7-1 can enable engineering of genetic resistance against the genus Fijivirus in multiple crop species.

## Methods

### Plant materials

A collection of 620 diverse maize inbred lines, 336 maize landraces, and 184 teosintes was provided by Prof. X.H. Yang (China Agricultural University) and Prof. B.S. Liu (Shandong Agricultural University) for analysis of the evolution of *ZmGDIα-hel*. Two populations were used for the fine mapping of *qMrdd1*. Population 1 (P1) was derived from a cross between the NT409 (susceptible) and NT411 (resistant) lines^37^, and population 2 (P2) is a recombinant inbred line (RIL) population derived from a cross between the HuangC (susceptible) and X178 (resistant) lines^56^. From the P2 population, a single F_10_ plant heterozygous at the *qMrdd1* locus was selected and self-pollinated twice to generate a pair of near-isogenic lines (NILs) differing solely at *qMrdd1*, NIL-R (with the resistance allele) and NIL-S (with the susceptibility allele). Transgenic plants were generated in the Maize Functional Genomic Project of China Agricultural University using the susceptible maize inbred line B73 as the recipient.

### Assessment of MRDD symptoms in the field

The fine-mapping populations were grown in multiple locations in China during 2013–2017 for a survey of MRDD symptoms under natural infection conditions, including Taian and Jining (Shandong province) in 2013, Taian, Jining, and Zaozhuang (Shandong province) in 2014, Taian and Kaifeng (Henan province) in 2015, Kaifeng in 2016, and Taian in 2017. Seeds were sown on May 15–26 to produce seedlings during the prime period of planthopper infestation. The MRDD severity in the field was classified into five grades (0, 0.25, 0.5, 0.75, and 1) in view of overall symptoms at the mature stage. Grade 0: high resistance without any symptoms; grade 0.25: moderate resistance with shortened plant height/internodes which equals to three-quarters of the normal plant; grade 0.5: moderate susceptibility with shortened plant height/internodes which equals to two-thirds of the normal plant; grade 0.75: susceptibility with severe shortened plant height/internodes which equals to one-half of the normal plant, accompanying with moderate waxy enations on the axial surfaces of upper leaves, and abnormal ear/tassel; grade 1: high susceptibility with typical MRDD symptoms, including severely stunted plant, extremely shortened internodes, massive waxy enations on the axial surfaces of upper leaves, and no ear/tassel. The DSI was used to represent MRDD severity, and was calculated as 1:

1 $${\mathrm{DSI}}\, \left( \% \right) = \, \Sigma \, ({\mathrm{Grade}}\,\times\,{\mathrm{Number}}\,{\mathrm{of}}\,{\mathrm{plants}}\,{\mathrm{in}}\,{\mathrm{grade}})\\ \, \times 100/(1\,\times\,{\mathrm{Total}}\,{\mathrm{number}}\,{\mathrm{of}}\,{\mathrm{plants}})$$

### Artificial inoculation of RBSDV

Maize seedlings at the two-leaf stages were artificially inoculated with RBSDV by allowing them to be fed on by viruliferous planthoppers for 3 days, and then transplanted into the field. Each plant was investigated for MRDD symptoms 90 days after inoculation. The scoring criteria and DSI calculation are the same as described above.

### Histological examination

The NIL-S and NIL-R seedlings were artificially inoculated with RBSDV. At the flowering stage, the upper blades and the middle part of the ninth internode were sampled, cut into small pieces, and then fixed for at least 2 h in 2.5% (v/v) glutaraldehyde and 0.1 M phosphate buffer (PBS, pH 7.4). After being washed three times with PBS, samples were post-fixed with 1% OsO_4_ in PBS buffer for 2 h and washed three more times with PBS. Samples were firstly dehydrated in a graded series of ethanol (30, 50, 70, 80, 90, and 100%), and further dehydrated twice with 100% ethanol. Thereafter, samples were dehydrated in a LEICA EM CPD300 critical-point dryer. In the end, dehydrated samples were coated with conductive materials, and photos were taken under an S-3400N Hitachi scanning electron microscope (Hitachi High Technologies, Tokyo, Japan).

### Sequential fine mapping of *qMrdd1*

Sequential fine mapping of *qMrdd1* was carried out by using the recombinant-derived progeny^39^. The markers in the mapped *qMrdd1* region were used to screen two mapping populations to identify recombinants, which were heterozygous at one flanking marker and homozygous at the other flanking marker. Recombinants were self-pollinated to produce fine-mapping progeny. The progeny was grown in the same plot under natural infection, and each individual was genotyped with markers in the heterozygous region of its parental recombinant and investigated for its disease incidence in the field. A DSI value was estimated for each of the three genotypes in a recombinant-derived selfed progeny. The two-tailed Student’s *t*-test was used to test for significant difference in DSI between any two of the three genotypes^37,39^, where significant difference indicates that *qMrdd1* is located in the heterozygous region of the parental recombinant, or otherwise, in the homozygous region or no *qMrdd1*.

### BAC sequencing and gene annotation

The markers in the final mapped *qMrdd1* region were used to screen the BAC libraries constructed from the resistant 1145 and susceptible HZ4 lines. Both 1145 and HZ4 BAC contigs were built, and the minimal overlapping BAC clones were subjected to sequencing, gene prediction (http://linux1.softberry.com/berry.phtml), and gene annotation (https://www.blast2go.com/). Together with the B73 reference sequence, predicted genes were aligned among 1145, HZ4, and B73 inbred lines.

### Construction of full-length cDNAs of *ZmGDIα-hel* and *ZmGDIα*

Rapid amplification of cDNA ends (RACE) was performed using the SMART RACE cDNA Amplification kit (TAKARA). Two primers, GDI-5′ and GDI-3′, each combined with the universal primer A mix, were used to amplify the 5′-cDNA and 3′-cDNA ends of *ZmGDIα-hel* from the resistant line X178, respectively. The 5′-RACE and 3′-RACE products were cloned into the pEasy-T1 vector for sequencing. The 5′-terminal and 3′-terminal sequences were merged to obtain the full-length *ZmGDIα-hel* cDNA. With the availability of the *ZmGDIα-hel* cDNA, primers Race-GDI were designed to amplify the HuangC cDNAs to obtain full-length *ZmGDIα* cDNAs.

### Transgenic populations of *ZmGDIα* and *ZmGDIα-hel*

One complementation, four over-expression, and two RNAi constructs were introduced into the maize recipient line B73 via *Agrobacterium tumefaciens* (strain EHA105). T_0_ positive plants from each transgenic event were self-crossed to produce T_1_ transgenic generations. T_2_ transgenic plants of the pCAMBIA3301-*ZmGDIα, pUbi:*:*ZmGDIα-EGFP* and *pUbi:*:*ZmGDIα*^*L*^*-EGFP* genotypes were crossed with X178 to generate T_2_F_1_ hybrids, which were further backcrossed to X178 twice to produce T_2_F_1_BC_2_ populations. For the two constructs of *ZmGDIα-hel*, *pUbi:*:*ZmGDIα-hel-EGFP* and *pUbi:*:*ZmGDIα-hel*^*L*^*-EGFP*, T_0_ transgenic plants were self-crossed twice to produce homozygous T_2_ transgenic plants, which were further selfed to produce pure T_4_ transgenic lines. The same operation was conducted for the two RNAi constructs.

### RNA extraction and real-time quantitative PCR

Total RNA was extracted from maize leaf using the EasyPure Plant RNA kit (TransGen Biotech, Beijing, China). First-strand cDNA was synthesized with 1.5 µg RNA using M-MLV reverse transcriptase (Invitrogen, Carlsbad, CA, USA). Real-time quantitative PCR (qRT-PCR) was performed on a Rotor-Gene Q 6000 cycler (Corbett Research, Cambridge, UK) at 45 cycles of 95 °C for 10 s and 60 °C for 30 s. By using SYBR Green (Takara Bio, Otsu, Japan), signal acquisition was conducted at the end of each amplification cycle. The primer pair qZmGDI was used to measure the transcript levels of *ZmGDIα*. The primer pair GAPDH-FP/RP was used to monitor *GAPDH2* expression as an internal control. The relative transcript level was calculated with the 2^−ΔΔCt^ method.

A pair of NILs was used to detect dynamic gene expression profiles for *ZmGDIα* and *ZmGDIα-hel*. Leaf tissues were taken from artificially inoculated plants (fed on by viruliferous planthoppers) at 2, 4, 8, 16, 32, and 58 dpi. The same sampling time points were conducted in non-inoculated plants (fed on by virus-free planthoppers) as controls. Each leaf tissue had three samples, and each sample was harvested from five plants. RNA expression for each sample was tested in triplicate.

### Reverse transcription PCR

We designed a specific primer pair, RT-ZmGDI-LS, for reverse transcription PCR (RT-PCR) assays that can distinguish long from short transcripts. For transgenic plants, gene expression level was assessed in maize leaves at the three-leaf stage via RT-PCR, with the primer pairs RT-ZmGDIα and RT-ZmGDIα-hel for *ZmGDIα* and *ZmGDIα-hel*, respectively.

### Immunoblot analysis

Total protein was extracted from 2 g maize leaves. The sampled tissue was ground in liquid nitrogen and resuspended in an equal volume (1:1 fresh weight/volume) of extraction buffer [50 mM Tris-MES, 10 mM EDTA, 17.1% (wt/vol) sucrose, 1 mM MgCl_2_, 5 mM DTT, 1 mM PMSF and 1% plant cocktail] (Sigma Aldrich) on ice for at least 30 min. The lysates were centrifuged at 13,200 × *g* for 20 min, and the supernatant was filtered and immunoprecipitated with anti-GFP magnetic agarose beads (MBL, Beijing, China) at 4 °C for 4 h. Thereafter, the tubes were placed on a magnetic rack (MBL, Beijing, China) for a few seconds to remove the supernatant. The beads were rinsed five times in extraction buffer, resuspended in 60 μl extraction buffer, and boiled for 10 min at 99 °C to release proteins.

The proteins were electrophoretically separated by SDS-PAGE (10% acrylamide gel) and transferred to a Hybond-p ECL PVDF membrane (GE Healthcare). After blocking in TBST (20 mM Tris, 0.137 M NaCl, pH 7.6, and 0.1% Tween-20) containing 5% nonfat milk at room temperature, the membrane was incubated overnight at 4 °C in Western Blot Immuno Booster Solution PBS (0.137 M NaCl, 2.68 mM KCl, 4 mM Na_2_HPO_4_, 1.76 mM KH_2_PO_4_, pH 7.4) with a Rabbit anti-GFP-Tag pAb (1:2000, ABclonal). After being washed in TBST at room temperature, the blots were incubated in Western Blot Immuno Booster Solution PBS with HRP goat anti-rabbit lgG secondary antibody (1:5000, ABclonal) for 2 h at room temperature. After addition of Novex ECL chemiluminescent substrate reagent (Thermo Fisher Scientific Inc., USA), signals were visualized under a chemiluminescence imaging system (Tanon 5200, Beijing, China).

### Split luciferase complementation assay

The full-length *ZmGDIα* and *ZmGDIα-hel* cDNA (short transcripts) and their gene segments were amplified and inserted into JW772-35S-CLuc to fuse with the C-terminal fragment of the luciferase gene (*cLUC*). In parallel, each of three viral genes (*S7-1*, *S8*, and *S10*) was amplified and inserted into JW771-35S-NLuc to fuse with the N-terminal fragment of the luciferase gene (*nLUC*). Transient expression in *N. benthamiana* leaf tissues was achieved by *Agrobacterium* infiltration. In brief, *Agrobacterium* strain EH105 containing the positive construct was cultured overnight in LB media. Equal amounts of OD600-normalized *Agrobacterium* cultures for CLuc and NLuc constructs were mixed to a final concentration of OD_600_ = 1.0 and then were collected and re-suspended in infiltration buffer (10 mM MES, pH 5.6, 10 mM MgCl_2_, 150 μM acetosyringone). The mixture was incubated at room temperature for ~3 h and then infiltrated into 4-week-old *N. benthamiana* plants. The infiltrated plants were placed at 28 °C for 72–84 h and then injected with 1 mmol/L beetle luciferase (Beetle luciferin, Promega) at the initial injection site, and then the fluorescence signal was measured.

### In vitro pull-down assay and competition experiment

The full-length *ZmGDIα* and *ZmGDIα-hel* cDNAs (short transcripts) were amplified and cloned into pGEX6P-1 with the GST-tag or pETM-40 with MBP-tag. Three viral genes, *S7-1*, *S-8*, and *S-10*, were separately cloned into pColdTF with His-tag or pHAT2 with His-tag. All recombinant plasmids were transformed into *Escherichia coli* strain BL21 (DE3) (TransGen Biotech, Beijing, China). The fusion proteins were purified with glutathione Sepharose 4B for GST-fused proteins (Yeasen, Shanghai, China), Amylose Resin for MBP-fused proteins (New England Biolabs, USA), or Ni Sepharose 6 Fast Flow for His-fused proteins (GE Healthcare) according to the manufacturers’ instructions.

For the pull-down assay, ZmGDIα-GST (or ZmGDIα-hel-GST) protein was suspended on glutathione agarose resin and gently rotated at 4 °C for 1 h, and then spun down to discard the supernatant. Each of three His-fused viral proteins was added into ZmGDIα-GST-bound (or ZmGDIα-hel-GST-bound) beads and incubated at 4 °C for 3 h. Subsequently, the beads were rinsed at least five times with washing buffer (136.8 mM NaCl, 2.7 mM KCl, 4 mM Na_2_HPO_4_, 1.8 mM KH_2_PO_4_). After being eluted from beads, the proteins were detected by immunoblotting with an anti-His antibody (MBL, Beijing, China). GST was used as the control.

In the competition experiment, we prepared six reactions in which MBP-P7-1 (50 μg) was firstly incubated in the anti-MBP-P7-1 beads, and then added His-ZmGDIα-hel (200 μg) and different amounts of GST-ZmGDIα (0, 12.5, 25, 50, 100, and 200 μg). In an inverse experiment, we fixed GST-ZmGDIα (200 μg) in each reaction and added variable amounts of His-ZmGDIα-hel (0, 12.5, 25, 50, 100, and 200 μg). GST-ZmGDIα and His-ZmGDIα-hel competed with each other in binding to MBP-P7-1. After 2 h incubation at 4 °C, the interacting proteins were pulled down using anti-MBP beads. Following thorough elution from anti-MBP beads, the proteins were separated and detected by immunoblotting with either His or GST antibody (MBL, Beijing, China). In addition, we used MBP-ZmGDIα^1–333^ and His-ZmGDIα-hel^1–318^ to conduct a protein competition assay to reveal the differences between the wild-type exon 10 and *helitron*-derived exon 10 residues in their interacting with GST-P7-1.

### Co-immunoprecipitation assay

The full-length *ZmGDIα* and *ZmGDIα-hel* cDNAs (short transcripts) were cloned into pSuper1300 tagged with the GFP. RBSDV *S7-1* was cloned into pSuper1300 tagged with the 6× Myc gene. The purified plasmids were separately transformed into *Agrobacterium* strain EH105. The positive clones carrying different constructs were equally mixed and co-infiltrated into *N. benthamiana* leaves. Total protein of the infiltrated leaf tissues was extracted as described above. The ZmGDIα-GFP and ZmGDIα-hel-GFP fusion proteins were co-immunoprecipitated with anti-GFP magnetic agarose beads (MBL, Beijing, China) at 4 °C for 3 h. The immunoprecipitates were detected by immunoblotting with anti-Myc antibody (MBL, Beijing, China).

### Protein homology modeling

Homology modeling was done by using SWISS-MODEL (https://www.swissmodel.expasy.org). The top-ranked model was selected from the set of candidate models^57^.

### Phylogenetic analysis of GDIα proteins

We downloaded Rab GDI protein sequences of multiple plant species, including Arabidopsis thaliana, Zea mays, Oryza sativa, Sorghum bicolor, Medicago truncatula, Solanum tuberosum, Glycine max, Nicotiana tabacum, Brachypodium distachyon, Brassica rapa, and Brassica napus, from the National Center for Biotechnology Information (http://www.ncbi.nlm.nih.gov/) database. A phylogenetic tree was constructed using the neighbor-joining method in the JTT matrix-based model in MEGA 7.0 (http://www.megasoftware.net)^58–61^. Bootstrap values from 1000 pseudo-replicates were used to provide support for the nodes in the phylogenetic tree.

### Nucleotide diversity

The intact *ZmGDIα* alleles were amplified from a panel of 24 diverse maize lines and 19 teosinte entries, and the intact *ZmGDIα-hel* alleles were amplified from 25 maize lines with *helitron* TEs in *ZmGDIα*. The PCR products were cloned into the pEasy-T1 vector (TransGen Biotech Co. Ltd, China) and at least five clones were selected for sequencing. All *ZmGDIα* and *ZmGDIα-hel* gene sequences were imported into ClustalX2 to produce a nucleotide alignment matrix. The alignment result was used for nucleotide diversity (*π*) analysis and Tajima’s *D*-test using DnaSP v5.1 software by using a 100-bp sliding window with a 25-bp step^62^.

### Minimum-spanning tree

A minimum-spanning tree was established based on nucleotide alignment among all sequenced *ZmGDIα* and *ZmGDIα-hel* alleles (excluding gaps) by using Arlequin3.5^63^, and visualized under HapStar v0.7^64^. The cycle size for a given haplotype was proportional to the sample size within the haplotype.

### Tests for neutrality

The HKA tests for neutrality were performed using DnaSP v5.1^62^. *ZmGDIα/ZmGDIα-hel* sequences of 49 individuals in the nucleotide diversity survey were aligned with that of *Tripsacum dactyloides*. Six previously described neutral loci^51^ were used as control genes (Supplementary Table 1). In HKA test^65^, an overall *χ*^2^ value was calculated by taking the sum of the individual *χ*^2^ values calculated for the six individual neutral loci. The overall *χ*^*2*^ values were then used to obtain an overall *P* value.

### Statistical analysis

Two-tailed Student’s *t*-test was used to test for significant difference between two groups. Paired two-tailed Student’s *t*-test was used to test for significant differences in mean viral copies between transgenic and non-transgenic plants. Multiple comparisons of DSI values among various genotypes were conducted using SAS 9.1 PROC general linear model with Tukey’s adjustment. *P* values and sample sizes (*n*) are indicated in individual figure legend. All values were represented as mean ± SEM. *P < 0.05; **P < 0.01; ***P < 0.001.

### Construction of vectors for functional validation

Procedure for constructing functional complementation, over-expression and RNAi vectors are described in the Supplementary Methods.

### Quantitative measurement of RBSDV

Real-time PCR assay to quantify RBSDV is described in the Supplementary Methods.

### Reporting summary

Further information on research design is available in the Nature Research Reporting Summary linked to this article.

## Supplementary information

Supplementary Information

Peer Review

Reporting Summary

Description of Additional Supplementary Files

Supplementary Data 1

Supplementary Data 2

Supplementary Data 3

Supplementary Data 4

Supplementary Data 5

Supplementary Data 6

Supplementary Data 7

Supplementary Data 8

## Data Availability

The authors declare that the data supporting the findings of this study are available within the paper and its supplementary information files. A reporting summary for this Article is available as a Supplementary Information file. The datasets generated and analyzed during the current study are available from the corresponding author upon request. The short (*ZmGDIα*) and long (*ZmGDIα*^*L*^) transcripts of HuangC are available at GenBank under primary accessions MK412408 and MK415062, respectively. The short (*ZmGDIα-hel*) and long (*ZmGDIα-hel*^*L*^) transcripts of X178 are available at GenBank under primary accessions MK412407 and MK415061, respectively. The *RabGDIα* homologs used in neutrality tests are available at GenBank under accessions MK412520 -MK412538 for teosinte, MK412542 -MK412566 for maize, and MK415063 for *Tripsacum*. *RabGDIα-hel* of *Zea mays* L. used in neutrality tests is available at GenBank under accessions MK420471 - MK420493. The source data underlying Figs. 2b–e, 3–5, as well as Supplementary Fig. 1b, 2, 3, 4b, 7b–f, 8b–e, 9b–e, 9g–j, 10b–g, 11, 15b, and 16b are provided as a Source Data file.

## Source Data

Source Data
