# Supplementary material for: A helitron-induced RabGDIα variant causes quantitative recessive resistance to maize rough dwarf disease
Source: Nat Commun. 2020 Jan 24;11:495. doi: 10.1038/s41467-020-14372-3 (PMC6981192; doi:10.1038/s41467-020-14372-3)
Supplement: Supplementary file 1 — Supplementary Information [file 41467_2020_14372_MOESM1_ESM.pdf]

A *helitron*-induced RabGDIα variant causes recessive quantitative resistance to maize rough dwarf virus disease

Liu *et al.*

Supplementary Methods

Construction of functional complementation vectors

The positive BAC clone 6-93 screened from the HZ4 BAC library was digested with *Bam*HI, and digested fragments larger than 10 kb in size were purified and ligated into the *Bam*HI-digested binary vector pCAMBIA3301. The *ZmGDIα*-taggedmarker MGS was used to screen the transgene-positive clones, which were thensequenced to confirm that they contained the intact *ZmGDIα* fragment. The constructused for transformation contained a 13.3-kb DNA fragment with the 8.8-kb codingregion, 3.1-kb promoter and 1.4-kb 3'-terminal region, and it was designated aspCAMBIA3301-*ZmGDIα*.

Construction of over-expression and RNAi vectors

The full-length cDNAs of *ZmGDIα* and *ZmGDIα-hel* were amplified from total HuangC and X178 cDNAs, respectively, by using the marker pair OEGDI-F/R and then cloned into the pEasy-T1 vector (TransGen Biotech, Beijing, China). Because both *ZmGDIα* and *ZmGDIα-hel* have long and short transcripts, we identified four positive clones harboring the corresponding four transcript isoforms. The cloned cDNAs were double-digested with *Xho*I and *Bam*HI and then sub-cloned into pEZS-NL to generate four EGFP-fused genes, *ZmGDIα^{L}-EGFP*, *ZmGDIα-EGFP*, *ZmGDIα-hel^{L}-EGFP*, and *ZmGDIα-hel-EGFP*. By using a pair of markers, OEGDI-F and GFP-1R, the four EGFP-fused genes were amplified and cloned into the *Xcm*I-digested pBCXUN vector. Thus far, four over-expression constructs, driven by the maize *Ubiquitin-1* promotor and terminated by the *nos* terminator, have been developed: *pUbi::ZmGDIα^{L}-EGFP*, *pUbi::ZmGDIα-EGFP*, *pUbi::ZmGDIα-hel^{L}-EGFP*, and *pUbi::ZmGDIα-hel-EGFP*.

To produce RNAi constructs, we identified two single-copy cDNA segments

common to both *ZmGDIα* and *ZmGDIα-hel* to design two primer pairs, iGDI-1 and iGDI-2. The *Sma*I-*Eco*RI and *Xba*I-*Xho*I sites were introduced into the 5′ terminus of the forward and reverse primers, respectively, for each pair of primers. Consequently, the 230-bp and 196-bp cDNA fragments were amplified with iGDI-1 and iGDI-2, respectively. The PCR products were cloned into the pEasy-T1 vector to produce the P1 plasmid. The P1 plasmid was digested with *Xho*I and *Eco*RI (New England Biolabs, Beverly, MA, USA) and then cloned into the RNAi vector pGreen-HY104 provided by Prof. SH Yang (China Agricultural University) to produce the P2 plasmid. The P1 and P2 plasmids were double-digested with *Xba*I and *Sma*I, and the digested *Xba*I-*Sma*I fragment of P1 was introduced into P2 to produce the P3 plasmid. Using the primer pair HY104-1, the RNAi fragments were amplified and cloned into the *Xcm*I-digested pBCXUN vector to produce two RNAi constructs: *pUbi::ZmGDIα*^{230}*-RNAi* and *pUbi::ZmGDIα*^{196}*-RNAi*.

Quantitative measurement of RBSDV

We used real-time quantitative PCR to directly quantify copy numbers of RBSDV[1]. Prior to this quantitative measurement, two standard curves were established: 1) concentration of the cDNA of the maize tubulin gene ZmTubulin (internal control): The cDNA sample prepared from non-inoculated maize leaves was diluted to five concentrations (30.16, 15.08, 3.016, 1.508, and 0.1508 ng μl^-1). A *ZmTubulin*-specific primer pair, pTubulin, was used in a real-time PCR assay. A linear regression relationship could be established between $\log_{10}$(cDNA concentration) and threshold cycles (Cts); 2) copy numbers of RBSDV-S10: The RBSDV-S10 segment (1677 bp) was amplified from the cDNAs of inoculated leaves using the primer pair pRBSDVs10 and cloned into the pEasy-T1 vector (TransGen Biotech, Beijing, China). The recombinant plasmid (5,605 bp) was extracted and diluted to a concentration of 175 ng μl^-1. The copy number of the RBSDV-S10 segment was calculated according to the following formula **2** and **3**:

Copy number = (amount × 6.02 × 10^{23}) / MV (**2**)

MV (g mol^-1) = length × 10^{9}(ng) × 660 (**3**)

where 'amount' equals the concentration of the recombinant plasmid (ng μl^-1), 6.02 × 10^{23} is the Avogadro's constant, 'length' is the number of base pairs in the target double-stranded DNA, and 660 is the average molecular weight per base pair (Daltons bp^-1). Thus, the copy number of recombinant plasmid at a concentration of 175 ng μl^-1 was estimated to be 2.847 × 10^{10} copies μl^-1. This is also the initial copy number of RBSDV-S10.

The recombinant plasmid was diluted with cDNA from non-infected leaf in tenfold increments to form 10 serial standard samples at concentrations from 2.847 × 10^{8} to 0.2847 copies μl^-1. The Ct value of each standard sample was obtained by using the viral primer pair qRBSDV on RBSDV-S10 fragment. Thus far, the standard curve was drawn by comparing the $\log_{10}$ (RBSDV-S10 copies μl^-1) values of the 10 standard samples with the corresponding Ct values.

The top leaf from the inoculated plant at 58 dpi was sampled to extract total RNA. A 1.5-μg sample of total RNA was reversely transcribed into cDNA using an M-MLV reverse transcriptase (Invitrogen, Carlsbad, CA, USA). The internal control *ZmTubulin* was used to calibrate the reverse transcription efficiency of each sample. The Ct value of *ZmTubulin* in each sample was detected by using the primer pair pTubulin, which was used to directly compare the *ZmTubulin* standard cure to obtain the concentration of *ZmTubulin* cDNA. Meanwhile, the Ct value of RBSDV in the same sample was detected by using the primer pair qRBSDV, which was used to obtain the RBSDV copy number according to the RBSDV-S10 standard curve. With both the *ZmTubulin* cDNA concentration and the RBSDV copy number in hand, we could estimate the copy numbers of RBSDV for each sample under the standard

ZmTubulin cDNA concentration (1.508 ng μl^-1) based on the following formula **4**:

Calibrated RBSDV copies μl^-1 = (1.508 ng μl^-1) / (sample cDNA concentration ng ul^-1) × (sample RBSDV copies μl^-1) **(4)**

In 2017, we artificially inoculated the $T_2F_1BC_2$ seedlings with the fixed endogenous *ZmGDIα-hel* alleles that were derived from two independent transgenic events expressing *pUbi*::*ZmGDIα-EGFP*. The artificial inoculation was conducted in triplicates, two in Beijing (with viruliferous planthoppers separately reared in Baoding and Nanjing) and one in Taian (with viruliferous planthoppers reared in Nanjing). The top leaves at 58 days post inoculation (dpi) were harvested from all plants, and then mixed into two samples, transgenic and non-transgenic samples, based on their genotypes. In 2018, the artificial inoculation was also conducted in triplicates in Beijing with viruliferous planthoppers reared in Nanjing. Each sample was performed in three technical replicates to calculate mean values of RBSDV copy numbers. Two-tailed Student's *t*-test was used to test for significant differences in mean viral copies between transgenic and non-transgenic plants.

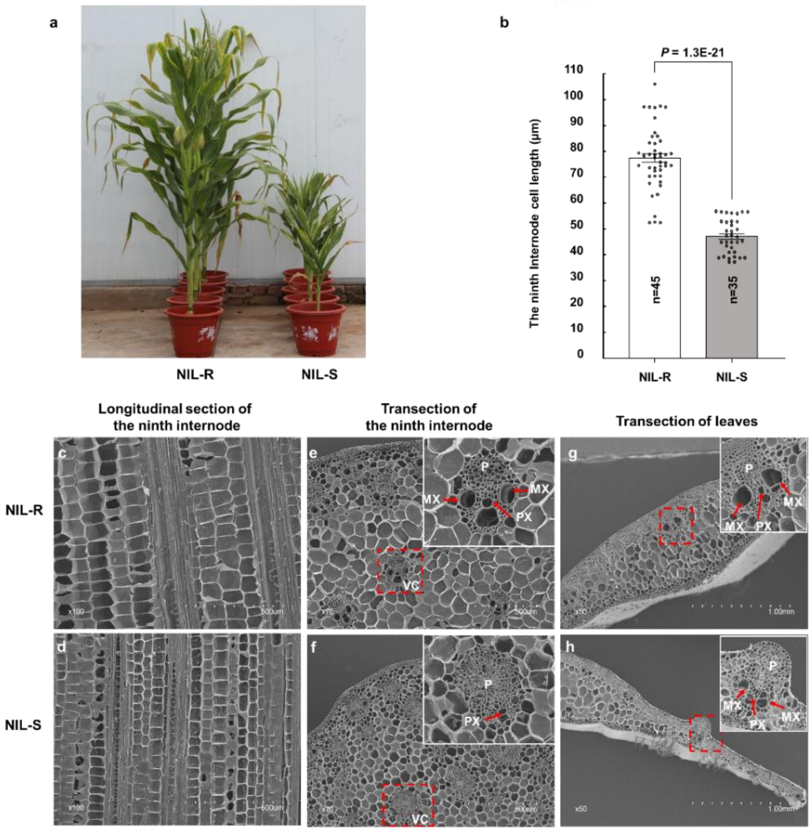

Supplementary Figure 1. Cellular ultrastructure of MRDD symptoms. a, Resistance performance of two NILs at 58 days after RBSDV inoculation. Healthy NIL-R plants are at left and diseased NIL-S plants at right. **b,** Cell lengths of the ninth internodes between two NILs. The healthy NIL-R plants (left) have longer cells than the diseased NIL-S plants (right). The numbers of plants tested are indicated in parentheses (NIL-R/NIL-S). Values are means ± SEM. Statistical significance was determined using two-tailed Student's *t*-test. Each dot represents the cell length of the ninth internode of a single tested plant. Source data are provided as a Source Data file. **c,d,** Longitudinal sections of the ninth internodes from the healthy NIL-R (**c**) and diseased NIL-S (**d**) plants. The cells of diseased NIL-S plants apparently are smaller in size relative to those of the healthy NIL-R. Scale bars, 500 μm. **e,f,** Transections of the ninth internodes from the healthy NIL-R (**e**) and diseased NIL-S (**f**) plants. Compared with the healthy NIL-R, the diseased NIL-S is characterized by an aberrant vascular cylinder with supernumerary phloem cells and atrophic xylem. Scale bars, 500 μm. VC: vascular cylinder; MX: metaxylem; PX: protoxylem; P: phloem. The dashed rectangle was annotated at the top right. **g,h,** Transections of upper leaves from the healthy NIL-R (**g**) and diseased NIL-S (**h**) plants. In diseased NIL-S, waxy enations on the abaxial surface actually consist of massive, irregular phloem cells. Scale bars, 1.00 mm. MX: metaxylem; PX: protoxylem; P: phloem. The dashed rectangle is annotated at the top right. The experiments were independently repeated nine times for '**c,d**', six times for '**e,f**', seven times for '**g**', and eight times for 'h', respectively, with similar results. Source data underlying Supplementary Figure 1b are provided as a Source Data file.

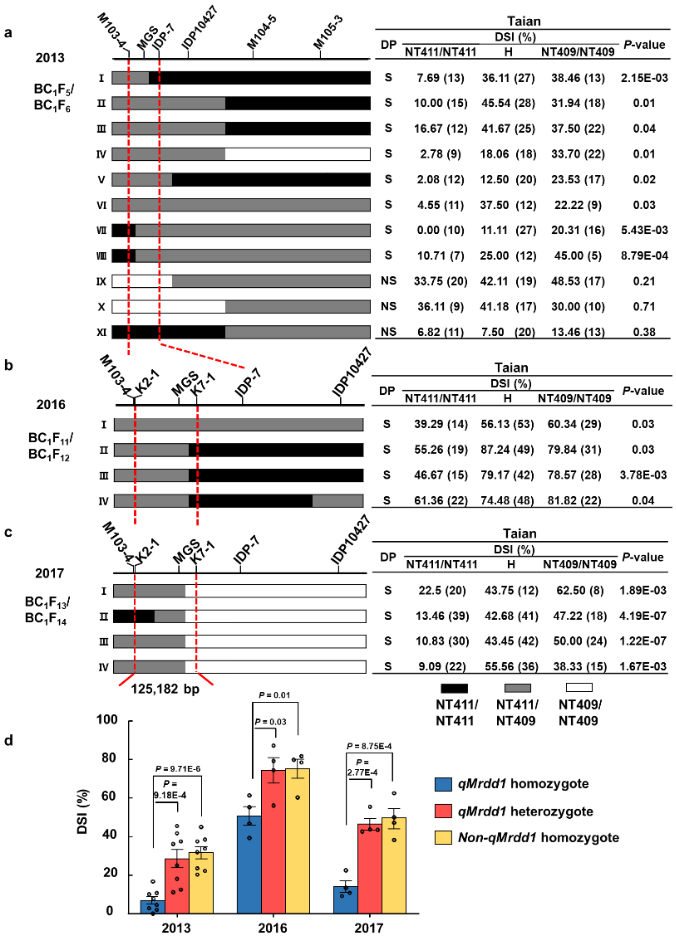

Supplementary Figure 2. Sequential fine-mapping of *qMrdd1* in the P1 population. a-c, Sequential fine-mapping of *qMrdd1* in 2013 (**a**), 2016 (**b**) and 2017 (**c**). Analysis of 11 BC_1F_5 (backcrossed once, then selfed five times) recombinants enabled us to restrict the location of *qMrdd1* to an interval flanked by the markers M103-4 and IDP-7 (**a**). Three further recombinants allowed us to refine the right-flanking marker from IDP-7 to K7-1 (**b**). In 2017, with four more recombinants, *qMrdd1* was restricted to the K2-1/K7-1 interval (**c**). Black, gray, and white rectangles represent homozygous NT411/NT411, heterozygous NT409/NT411, and homozygous NT409/NT409 chromosomal segments at *qMrdd1*, respectively. DP: deduced phenotypes in a recombinant-derived progeny as either segregation (S) or non-segregation (NS) at *qMrdd1*; The numerals in parentheses represent the numbers of plants for each genotype in the progeny; The significance in the DSI values between NT411/NT411 and NT409/NT409 genotypes was determined by two-tailed Student's *t*-test. *P*-value < 0.05 means *qMrdd1* localized to the heterozygous region; or otherwise, *qMrdd1* localized to the homozygous region or no *qMrdd1*. **d,** Genetic effect of *qMrdd1* in resistance to MRDD as calculated in 2013, 2016, and 2017. DSI values are means ± SEM. Multiple comparisons among *qMrdd1* homozygous, *qMrdd1* heterozygous, and non-*qMrdd1* homozygous genotypes were conducted using SAS 9.1 PROC general linear model with Tukey's adjustment. Each dot represents the DSI value of each genotype within a single recombinant-derived progeny. Source data are provided as a Source Data file.

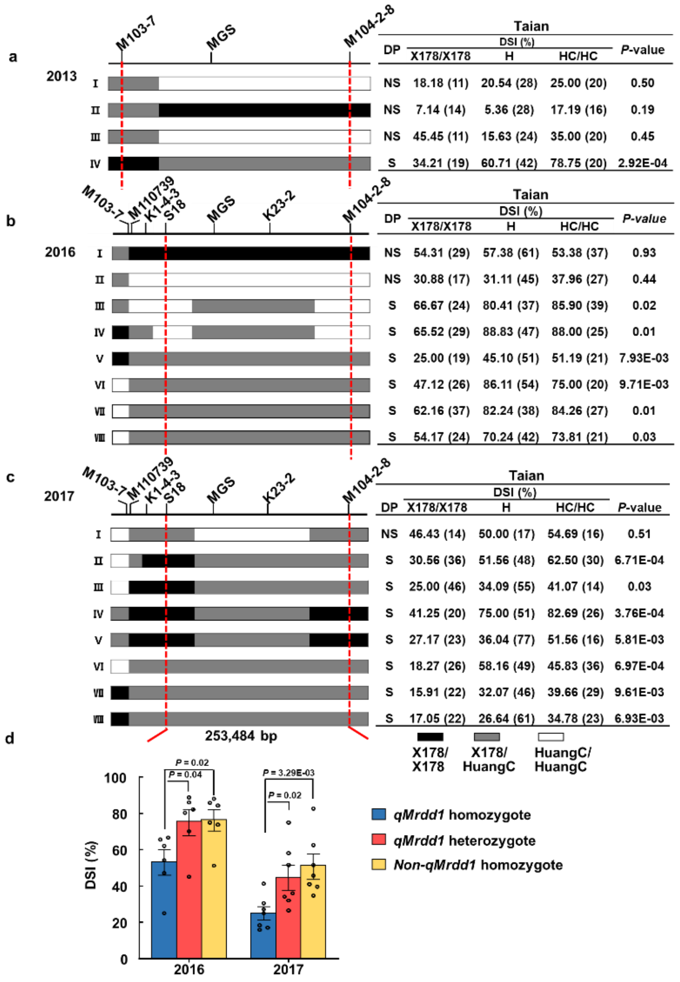

Supplementary Figure 3. Sequential fine-mapping of *qMrdd1* in the P2 population. a-c, Sequential fine-mapping of *qMrdd1* in 2013 (**a**), 2016 (**b**) and 2017 (**c**). In 2013, progeny testing allowed us to narrow down the location of *qMrdd1* to a region between markers M103-7 and M104-2-8 (**a**). During 2014–2016, *qMrdd1* was mapped to a region flanked by markers S18 and M104-2-8 (**b**). In 2017, with eight more recombinants, *qMrdd1* was confirmed to be in the interval of S18/M104-2-8 (**c**). Black, gray, and white rectangles correspond to homozygous X178/X178, heterozygous X178/HuangC, and homozygous HuangC/HuangC chromosomal segments at *qMrdd1*, respectively. DP: deduced phenotypes in a recombinant-derived progeny as either segregation (S) or non-segregation (NS) at *qMrdd1*; The numerals in parentheses represent the numbers of plants for each genotype in the progeny. The significance in the DSI values between X178/X178 and HuangC/HuangC genotypes was determined by two-tailed Student's *t*-test. *P*-value < 0.05 means that *qMrdd1* localized to the heterozygous region; or otherwise, *qMrdd1* localized to the homozygous region or no *qMrdd1*. **d,** Genetic effect of *qMrdd1* in resistance to MRDD as calculated in 2013, 2016, and 2017. DSI values are mean ± SEM. Multiple comparisons among *qMrdd1* homozygous, *qMrdd1* heterozygous, and non-*qMrdd1* homozygous genotypes were conducted using SAS 9.1 PROC general linear model with Tukey's adjustment. Each dot represents the DSI value of each genotype within a single recombinant-derived progeny. Source data are provided as a Source Data file.

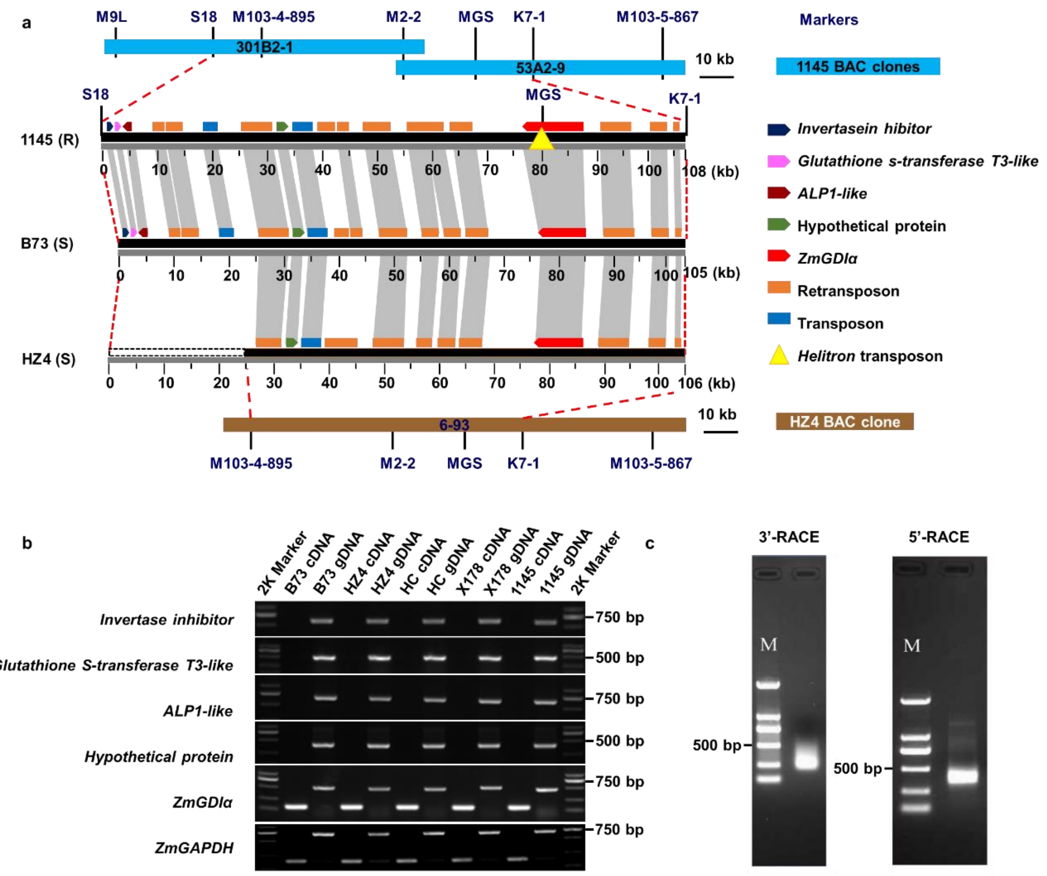

Supplementary Figure 4. Identification of the candidate gene in the *qMrdd1* region. a, The predicted genes in the *qMrdd1* region flanked by markers S18 and K7-1. The markers in the mapped *qMrdd1* region were used to screen 1145 and HZ4 BAC libraries. Two 1145 and one HZ4 positive BAC clones, separately marked as blue bars and brown bar, were sequenced for gene prediction. Alignment of predicted genes among 1145, HZ4, and B73 revealed five non-transposon-related genes in the *qMrdd*1 region, encoding, respectively, an invertase inhibitor, glutathione S-transferase T3-like, ALP1-like, a hypothetical protein, and Rab GDP dissociation inhibitor alpha (RabGDIα). Colored arrows show the position and orientation of predicted genes. The blue and orange boxes indicate the locations of transposons and retrotransposons, respectively. Light-gray shades denote collinear coding regions among 1145, B73, and HZ4. The yellow triangle represents the inserted *helitron* transposon. **b,** Gene expression assay for five predicted genes. Seedlings at the three-leaf stage were collected from resistant inbred lines 1145 and X178 as well as susceptible inbred lines B73, HZ4, and HC, and their cDNAs and genomic DNAs were used as templates for RT-PCR assay. For each of the first four predicted genes, no gene expression was detected in any of the five lines tested. By contrast, *RabGDIα* did show gene expression, but had no difference in gene expression levels between resistant and susceptible lines. *ZmGAPDH* was used as an internal control. Source data are provided as a Source Data file. **c,** The 3'-RACE and 5'-RACE PCR products of *ZmGDIα-hel* from the resistant line X178. The experiments shown in '**b**' and '**c**' were repeated three or more times independently, with similar results. Source data underlying Supplementary Figure 4b are provided as a Source Data file.

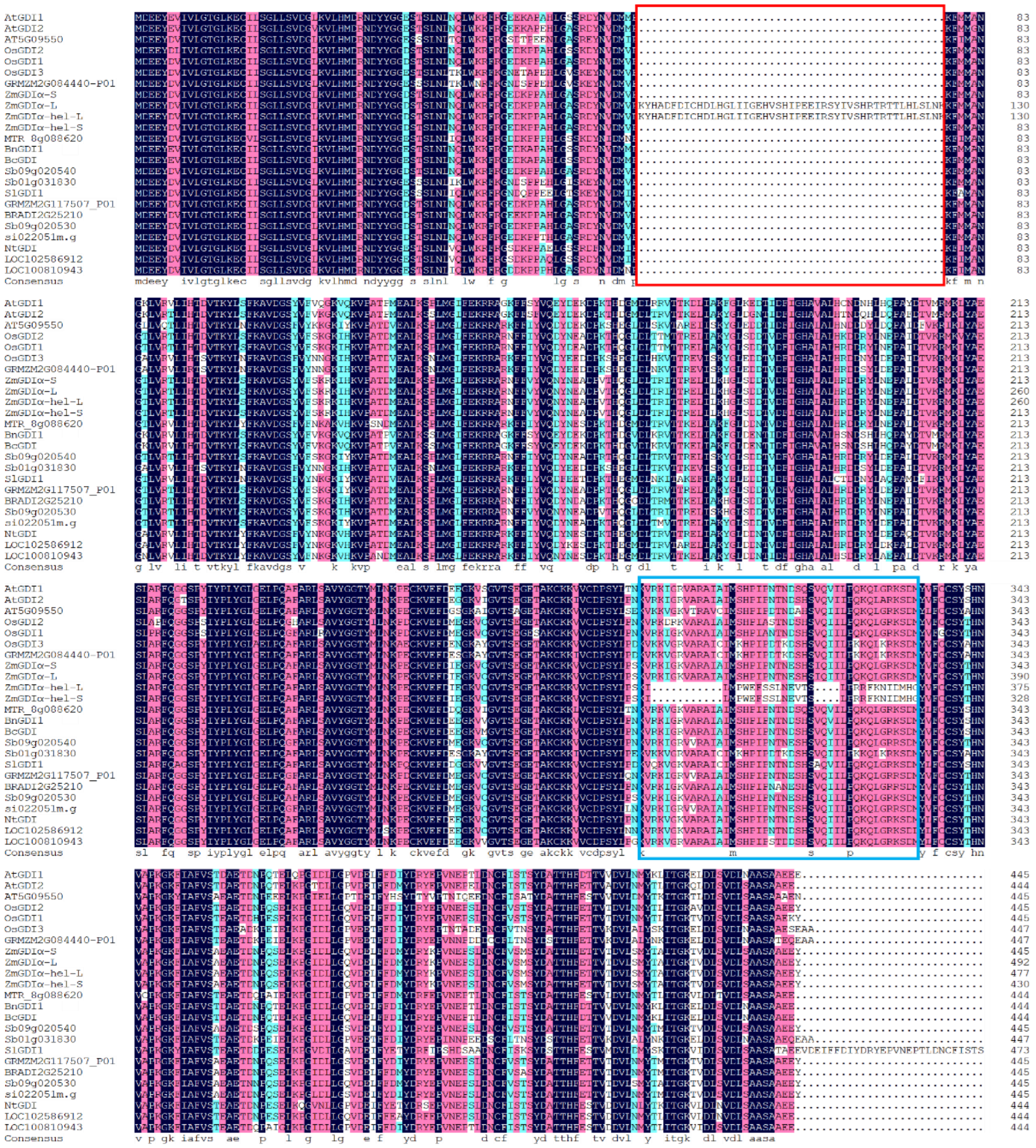

Supplementary Figure 5. Alignment of ZmGDIα orthologs of various plant species. ZmGDIα proteins were retrieved from *Arabidopsis thaliana*, *Zea mays*, *Oryza sativa*, *Sorghum bicolor*, and others. ZmGDIα-L and ZmGDIα-hel-L proteins are deduced from the long transcripts of inbred lines HuangC (susceptible) and X178 (resistant), respectively; while, ZmGDIα-S and ZmGDIα-hel-S proteins from the short transcripts of inbred lines Huang C (susceptible) and X178 (resistant), respectively. The extra exon 4 in either ZmGDIα-L or ZmGDIα-hel-L protein is unique to maize and boxed in red. The residues encoded by the wild-type exon 10 and *helitron*-derived exon 10 (ZmGDIα-hel-S and ZmGDIα-hel-L proteins only) are boxed in blue. A short stretch of 27 amino-acid residues encoded by the *helitron*-derived exon 10 is strictly confined to the ZmGDIα-hel protein.

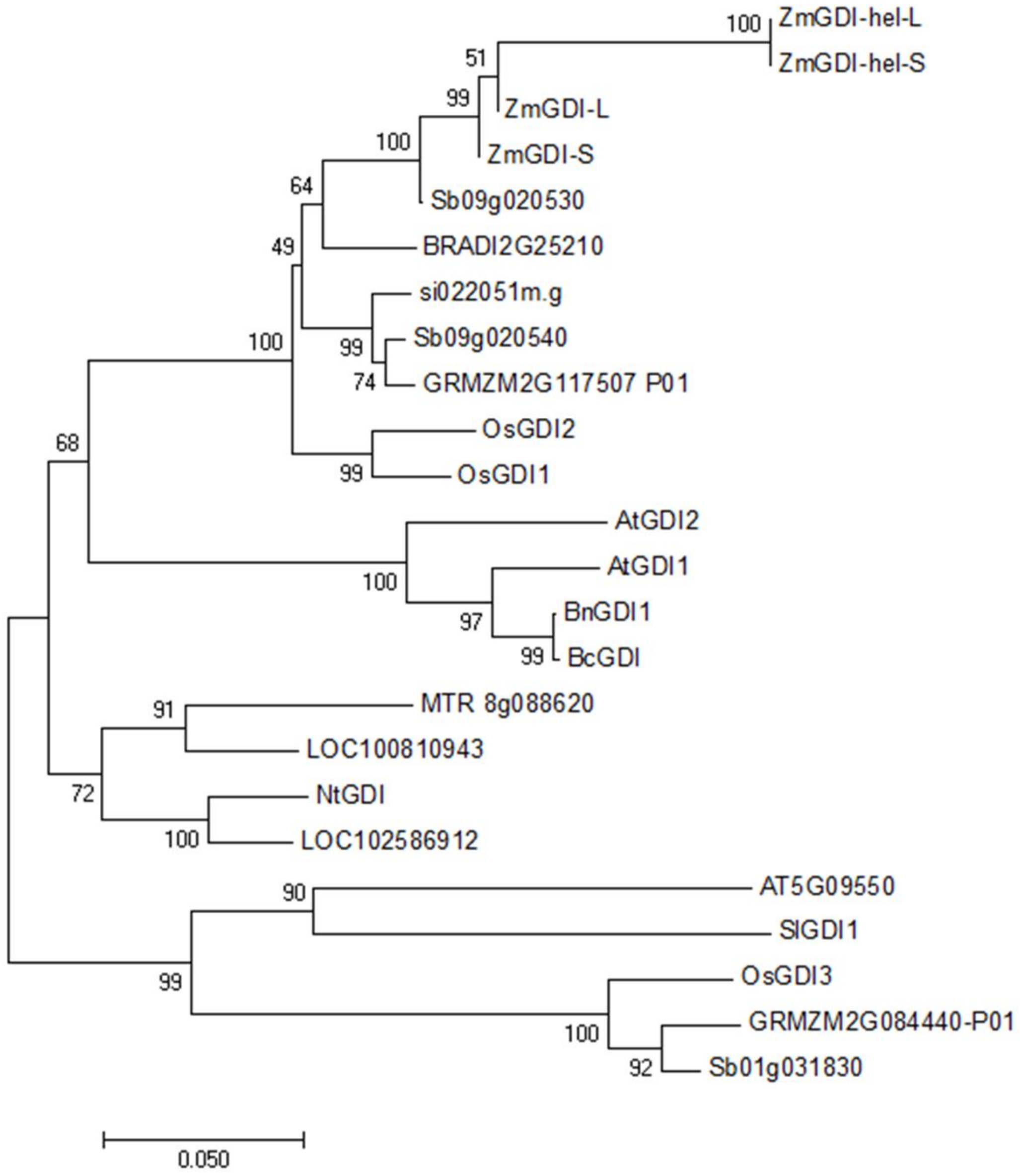

Supplementary Figure 6. Phylogenetic analysis of GDI from various plant species. GDI proteins were retrieved from *Arabidopsis thaliana* (At), *Zea mays* (Zm), *Oryza sativa* (Os), and *Sorghum bicolor* (Sb), and others. A phylogenetic tree was constructed using the maximum-likelihood method based on the JTT matrix-based model in MEGA 7.0. ZmGDIα-L and ZmGDIα-hel-L proteins are deduced from the long transcripts of inbred lines HuangC (susceptible) and X178 (resistant), respectively; while ZmGDIα-S and ZmGDIα-hel-S proteins are deduced from the short transcripts of inbred lines HuangC (susceptible) and X178 (resistant), respectively.

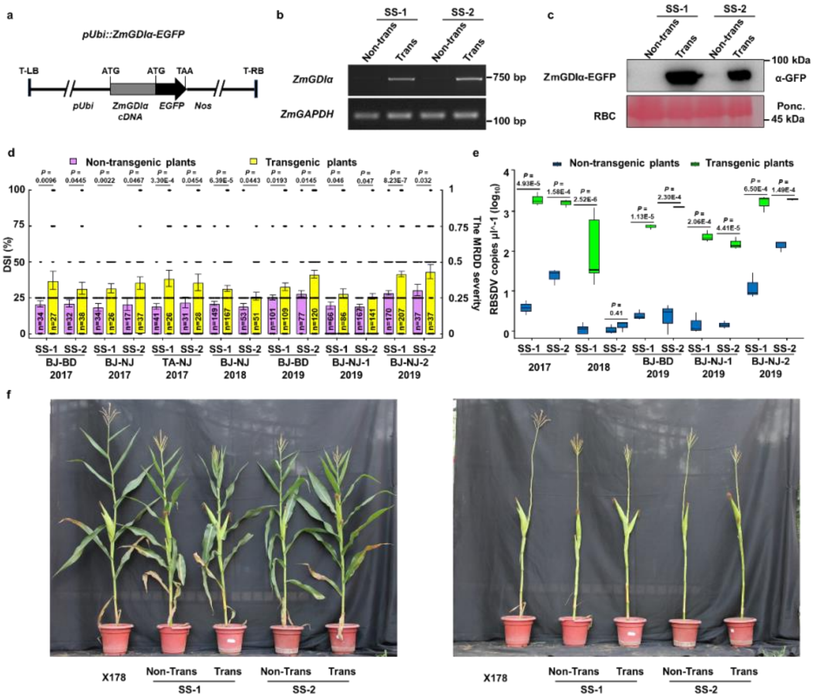

Supplementary Figure 7. Functional validation of the short *ZmGDIα* transcript. a, Structure of the *pUbi::ZmGDIα-EGFP* construct. *ZmGDIα*: short *ZmGDIα* transcript; T-RB and T-LB: right and left T-DNA borders, respectively. **b,** Validation of $T_2F_1BC_2$ transgenic plants by RT-PCR. A distinct band was observed in transgenic plants (Trans) but not in non-transgenic siblings (Non-trans). The maize gene *ZmGAPDH* was used as an internal control. The experiment was repeated three times independently, with similar results. **c,** Validation of transgenic plants by immunoblotting assay. The ZmGDIα-EGFP fusion protein was detected in transgenic plants but not in non-transgenic siblings. Rubisco (RBC) was used as a loading control. The experiment was repeated three times independently, with similar results. **d,** Disease severity index (DSI) values in $T_2F_1BC_2$ plants. The viruliferous planthoppers reared in Baoding (BD) and Nanjing (NJ) were used for artificial inoculation in Beijing and Taian. DSI values were estimated for both transgenic and non-transgenic $T_2F_1BC_2$ plants, and are denoted as mean ± SEM. The number of plants is indicated in each column. Each dot represents the disease severity of a single plant. **e,** The mean RBSDV copies μl^-1 ($\log_{10}$) and distribution in $T_2F_1BC_2$ plants. Two-tailed Student's *t*-test was used to test for significant differences between transgenic and non-transgenic plants. Leaf tissues were sampled three times independently as three biological replicates. Box edges represent quartiles, and the medians were shown with the central line in the boxes. **f,** The resistance performance of $T_2F_1BC_2$ progeny plants. Transgenic plants (Trans) were more susceptible to MRDD than non-transgenic siblings (Non-trans) with stunting plants and shortened internodes (especially those above ear leaves). The inbred line X178 was used as a resistant control. Source data underlying Supplementary Figure 7b-f are provided as a Source Data file.

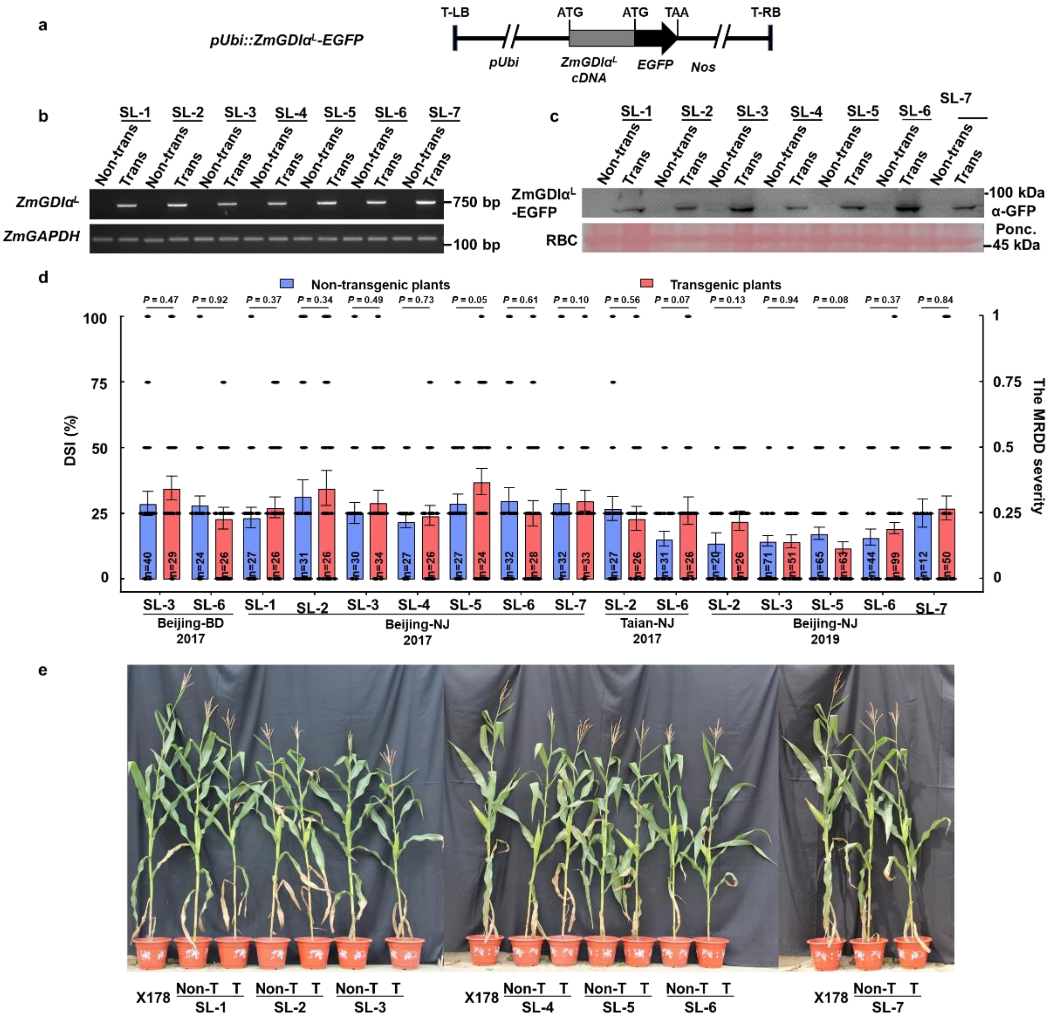

Supplementary Figure 8. Transgenic validation of the long *ZmGDIα* transcript. a, Structure of the *pUbi::ZmGDIα*^{L}*-EGFP* construct. *ZmGDIα*^{L}: the long *ZmGDIα* transcript; T-RB and T-LB: the right and left borders of T-DNA, respectively. **b,** Confirmation of transgenic plants using RT-PCR. A distinct RT-PCR band was observed in transgenic (Trans), but not in non-transgenic (Non-trans) plants. *ZmGAPDH* was used as an internal control. The experiment was repeated three times independently, with similar results. **c,** Confirmation of transgenic plants by immunoblotting assay. The ZmGDIα^{L}-EGFP fusion protein was detected in transgenic (Trans), but not in non-transgenic (Non-trans) plants. Rubisco (RBC) was used as a loading control. The experiment was repeated three times independently, with similar results. **d,** Disease severity index (DSI) between $T_2F_1BC_2$ transgenic and non-transgenic plants. The viruliferous planthoppers reared in Baoding (BD) and Nanjing (NJ) were used for artificial inoculation in Beijing and Taian. The values are mean ± SEM. Statistical significance was determined by using two-tailed Student's *t*-test. The numbers of plants are indicated within each column. Each dot represents the disease severity of a single plant. **e,** The resistance phenotypes of $T_2F_1BC_2$ progeny plants. All transgenic plants (T) showed the same as transgenic siblings (Non-T) at 90 dpi. The inbred line X178 was used as a resistant control. Source data underlying Supplementary Figure 8b-e are provided as a Source Data file.

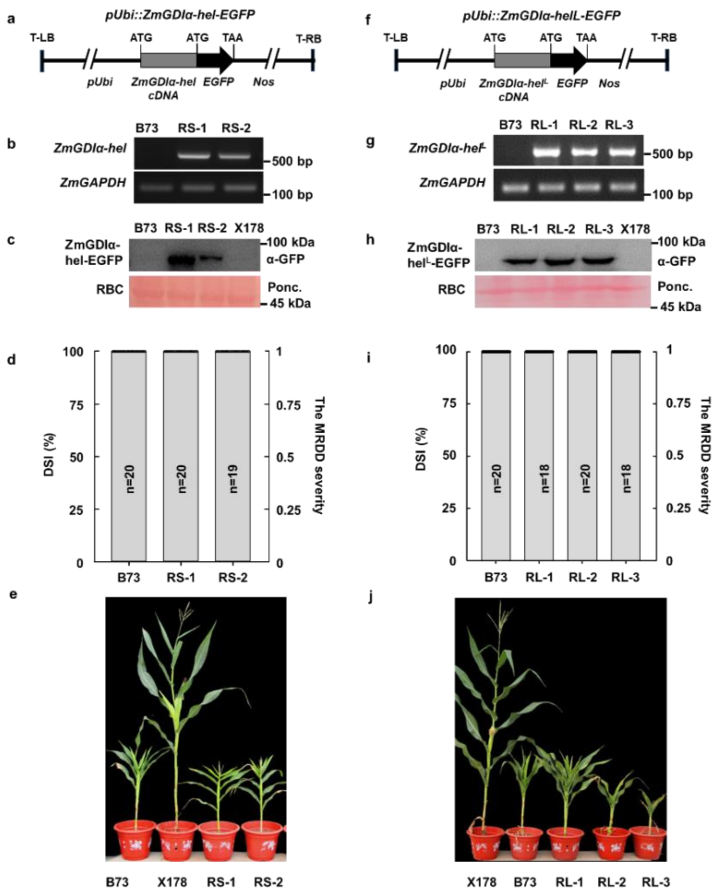

Supplementary Figure 9. Transgenic functional analysis of *ZmGDIα-hel*. a,f, Structure of the *pUbi::ZmGDIα-hel-EGFP* (**a**) and *pUbi::ZmGDIα-hel*^{L}*-EGFP* (**f**) constructs. Both *ZmGDIα-hel-EGFP* and *ZmGDIα-hel*^{L}*-EGFP* fusion genes are driven by the maize *Ubiqutin-1* promoter (*pUbi*) and terminated by the *Nos* terminator (*Nos*). *ZmGDIα-hel* and *ZmGDIα-hel*^{L}: the short and long *ZmGDIα-hel* transcripts, respectively. T-RB and T-LB: the right and left borders of T-DNA, respectively. **b,g,** Confirmation of transgenic plants by RT-PCR. A distinct RT-PCR band was observed in each transgenic plant, but not in the recipient B73 plant. *ZmGAPDH* was used as an internal control. The experiment was repeated three times independently, with similar results. **c,h,** Confirmation of transgenic plants by immunoblotting with the anti-GFP antibody. The ZmGDIα-hel-EGFP (**c**) and ZmGDIα^{L}-hel-EGFP (**h**) fusion proteins were detected in transgenic, but not B73 plants. Rubisco (RBC) was used as a loading control. The experiment was repeated three times independently, with similar results. **d,i,** The DSI values between transgenic and B73 plants. Together with B73, T$_4$ transgenic plants for each transgenic event were artificially inoculated at the two-leaf stage by allowing them to be fed on by viruliferous planthoppers. All transgenic and B73 plants were severely infected by RBSDV. The numbers of plants are indicated within each column. Each dot represents the disease severity of a single plant. **e,j,** The resistance phenotypes of T$_4$ transgenic plants. All transgenic and B73 plants showed severely stunted growth at 90 dpi. The inbred line X178 was used as a resistant control. Source data underlying Supplementary Figure 9b-e and 9g-j are provided as a Source Data file.

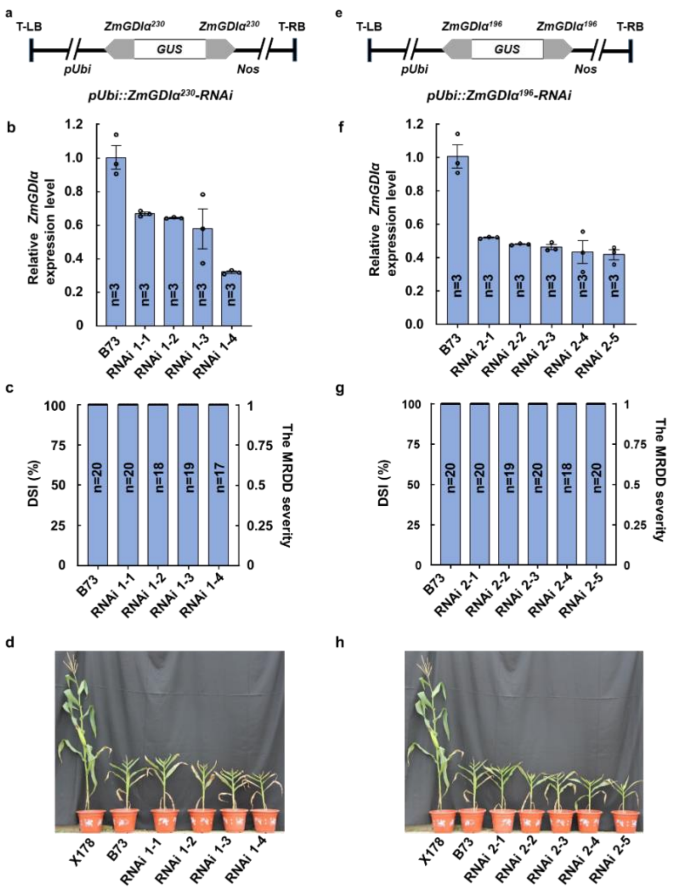

Supplementary Figure 10. RNAi-mediated transgenic functional assay of *ZmGDIα*. a,e, The schematic diagrams for RNAi constructs: *pUbi*::*ZmGDIα*^{230}-*RNAi* (**a**) and *pUbi*::*ZmGDIα*^{196}-*RNAi* (**e**). The *RNAi* construct is driven by the maize *Ubiquitin-1* promoter (*pUbi*) and terminated by the *Nos* terminator (*Nos*). T-RB and T-LB: the right and left borders of T-DNA, respectively. **b,f,** The relative expression levels of transgenic plants. Four (RNAi1-1 to -4) (**b**) and five (RNAi2-1 to -5) (**f**) independent transgenic events were obtained for *pUbi*::*ZmGDIα*^{230}-*RNAi* and *pUbi*::*ZmGDIα*^{196}-*RNAi*, respectively. For each transgenic event, the gene expression level of *ZmGDIα* is dramatically reduced compared to its susceptible recipient B73. The values are mean ± SEM. Three samples were taken as biological replicates (n=3). Each dot indicates the expression level of a single biological replicate. **c,g,** The DSI values between transgenic and B73 plants. Together with B73, T_4 transgenic plants for each transgenic event were artificially inoculated at the two-leaf stage by allowing them to be fed on by viruliferous planthoppers. The numbers of plants are indicated within each column. Just like their recipient B73, all transgenic plants from four RNAi1 (**c**) and five RNAi2 (**g**) transgenic events were severely infected by RBSDV. Each dot represents the disease severity of a single plant. **d,h,** The resistance phenotypes of T_4 transgenic plants. All transgenic plants from *pUbi*::*ZmGDIα*^{230}-*RNAi* (**d**) and *pUbi*::*ZmGDIα*^{196}-*RNAi* (**h**) showed severely stunted growth at 90 dpi. The inbred line X178 was used as a resistant control. Source data underlying Supplementary Figure 10b, 10c, 10f, and 10g are provided as a Source Data file.

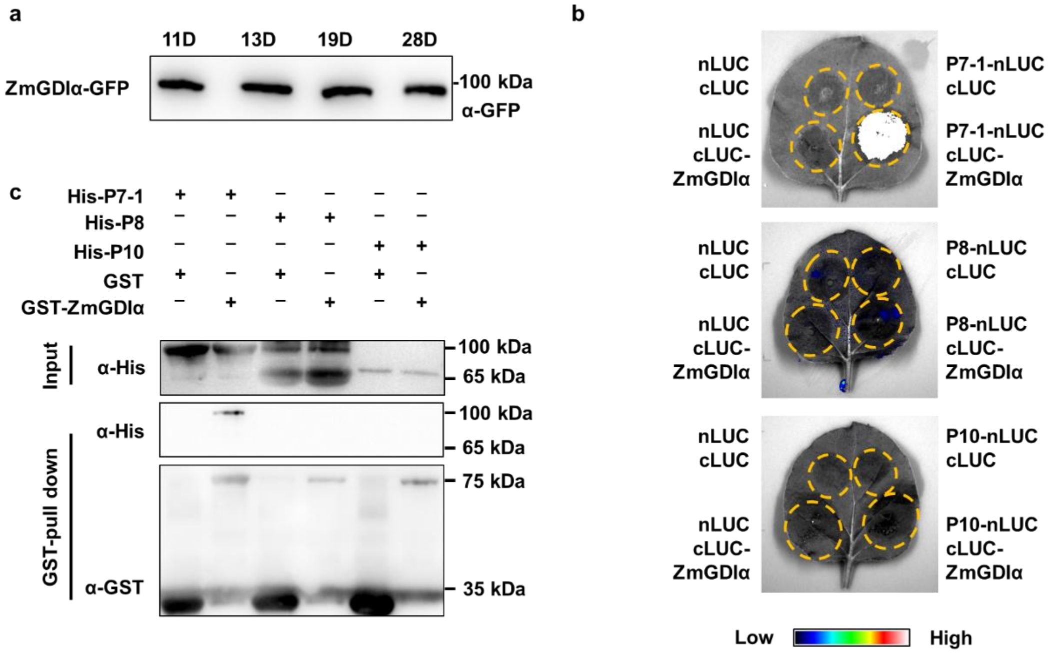

Supplementary Figure 11. Identification of the viral effector that recruits ZmGDIα. a, Immunoblot of ZmGDIα-EGFP fusion proteins. The transgenic plants over-expressing *ZmGDIα-EGFP* were artificially inoculated with RBSDV at the two-leaf stage, and total proteins were extracted from leaf tissues at 11, 13, 19, and 28 dpi. The ZmGDIα-EGFP fusion protein was immunoprecipitated, detected by immunoblotting with anti-GFP antibody, and extracted from the gel for mass spectrometry analysis. The experiment was repeated three times independently, with the similar results. **b,** Split-luciferase complementation assay to detect interactions of ZmGDIα with three viral proteins. Fluorescence signal was detected when cLUC-ZmGDIα was co-expressed with P7-1-nLUC (top), but not P8-nLUC (middle) or P10-nLUC (bottom) in *N. benthamiana* leaves. More than five leaves were examined for each pair of fusion proteins. **c,** GST pull-down assay to detect interactions of ZmGDIα with three viral proteins in vitro. Three His-fusion viral proteins, His-P7-1, His-P8 and His-P10, were pulled down by GST-ZmGDIα and detected by immunoblotting with an anti-His antibody. Apart from His-P7-1, neither His-P8 nor His-P10 fusion protein was detected. The experiment was repeated three times independently, with the similar results. Source data are provided as a Source Data file.

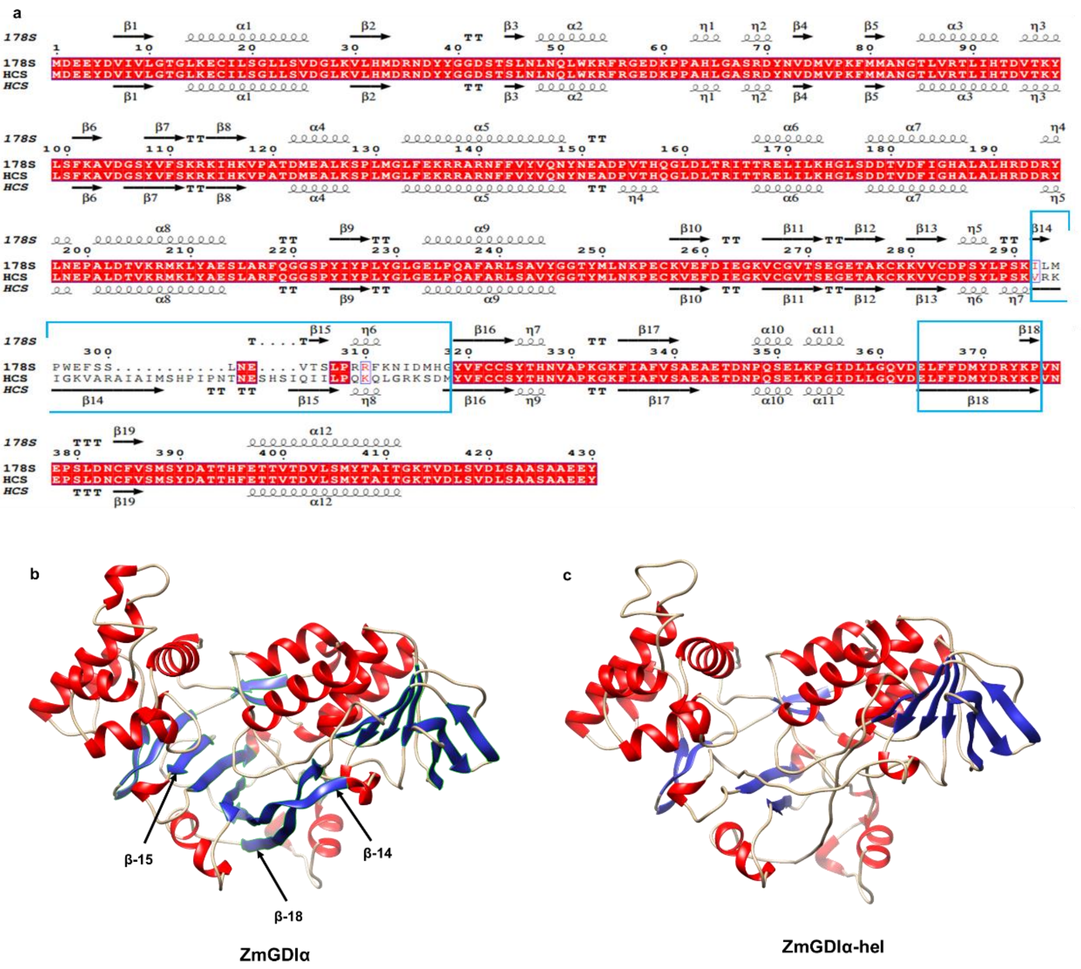

Supplementary Figure 12. Alignment of ZmGDIα/ZmGDIα-hel and their structures. a, Alignment of ZmGDIα (HCS, from the susceptible line HuangC) and ZmGDIα-hel (178S, from the resistant line 178) for both amino-acid sequences and protein structures. Differences in protein structure between HCS and 178S were observed in two regions as marked in two boxes: the first region corresponds to the exon 10-encoded residues where the *ZmGDIα* exon 10 encodes 43 residues that mainly forms two β sheets, β14 and β15, whereas the *ZmGDIα-hel* exon 10 encodes only 27 residues that almost abolish two β sheets. The second region covers ZmGDIα β18, which is largely depleted in ZmGDIα-hel, although both ZmGDIα and ZmGDIα-hel share the same residues in this region. **b,c,** Protein 3D structure predicted by protein homology modelling using SWISS-MODEL (https://www.swissmodel.expasy.org). The β-sheets are shown in blue and the α-helices are shown in red. ZmGDIα-hel lost three β-sheets compared with ZmGDIα.

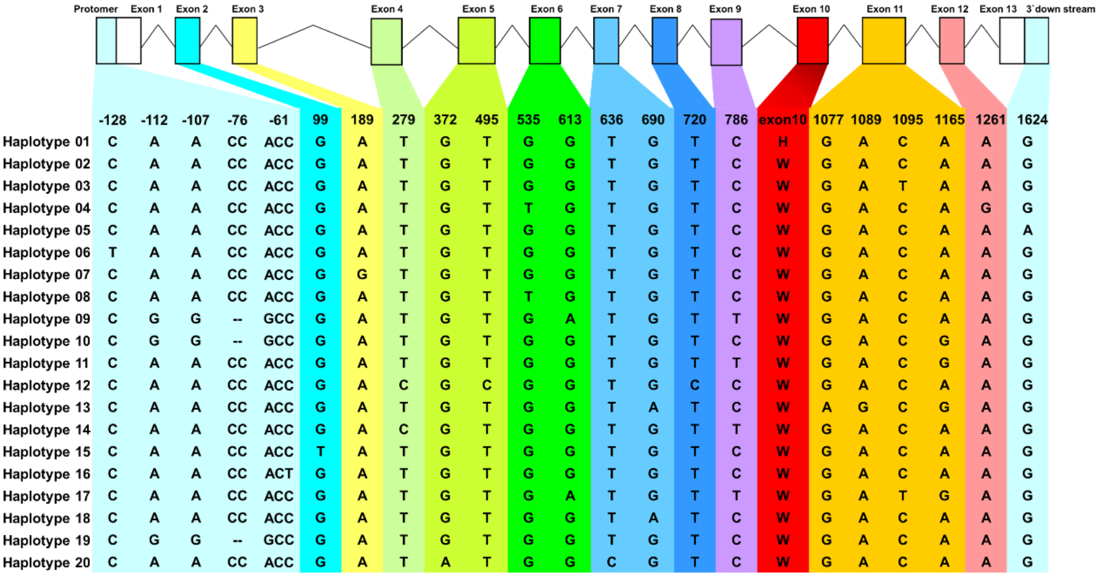

	-128	-112	-107	-76	-61	99	189	279	372	495	535	613	636	690	720	786	exon10	1077	1089	1095	1165	1261	1624
Haplotype 01	C	A	A	CC	ACC	G	A	T	G	T	G	G	T	G	T	C	H	G	A	C	A	A	G
Haplotype 02	C	A	A	CC	ACC	G	A	T	G	T	G	G	T	G	T	C	W	G	A	C	A	A	G
Haplotype 03	C	A	A	CC	ACC	G	A	T	G	T	G	G	T	G	T	C	W	G	A	T	A	A	G
Haplotype 04	C	A	A	CC	ACC	G	A	T	G	T	T	G	T	G	T	C	W	G	A	C	A	G	G
Haplotype 05	C	A	A	CC	ACC	G	A	T	G	T	G	G	T	G	T	C	W	G	A	C	A	A	A
Haplotype 06	T	A	A	CC	ACC	G	A	T	G	T	G	G	T	G	T	C	W	G	A	C	A	A	G
Haplotype 07	C	A	A	CC	ACC	G	G	T	G	T	G	G	T	G	T	C	W	G	A	C	A	A	G
Haplotype 08	C	A	A	CC	ACC	G	A	T	G	T	T	G	T	G	T	C	W	G	A	C	A	A	G
Haplotype 09	C	G	G	--	GCC	G	A	T	G	T	G	A	T	G	T	T	W	G	A	C	A	A	G
Haplotype 10	C	G	G	--	GCC	G	A	T	G	T	G	G	T	G	T	C	W	G	A	C	G	A	G
Haplotype 11	C	A	A	CC	ACC	G	A	T	G	T	G	G	T	G	T	T	W	G	A	C	G	A	G
Haplotype 12	C	A	A	CC	ACC	G	A	C	G	C	G	G	T	G	C	C	W	G	A	C	A	A	G
Haplotype 13	C	A	A	CC	ACC	G	A	T	G	T	G	G	T	A	T	C	W	A	G	C	G	A	G
Haplotype 14	C	A	A	CC	ACC	G	A	C	G	T	G	G	T	G	T	T	W	G	A	C	A	A	G
Haplotype 15	C	A	A	CC	ACC	T	A	T	G	T	G	G	T	G	T	C	W	G	A	C	A	A	G
Haplotype 16	C	A	A	CC	ACT	G	A	T	G	T	G	G	T	G	T	C	W	G	A	C	A	A	G
Haplotype 17	C	A	A	CC	ACC	G	A	T	G	T	G	A	T	G	T	T	W	G	A	T	G	A	G
Haplotype 18	C	A	A	CC	ACC	G	A	T	G	T	G	G	T	A	T	C	W	G	A	C	A	A	G
Haplotype 19	C	G	G	--	GCC	G	A	T	G	T	G	G	T	G	T	C	W	G	A	C	A	A	G
Haplotype 20	C	A	A	CC	ACC	G	A	T	A	T	G	G	C	G	T	C	W	G	A	C	A	A	G

Supplementary Figure 13. The 20 haplotypes detected at the *ZmGDIα* locus. A panel of 24 *ZmGDIα-hel* maize lines, 25 *ZmGDIα* maize lines, and 19 teosinte entries were re-sequenced for their intact *ZmGDIα-hel* or *ZmGDIα* alleles. Together with the polymorphic *helitron* TEs, 24 SNPs were found in the gene region, resulting in 20 haplotypes. Pink blue boxes represent the 3′- and 5′ UTRs, the other colored boxes denoted exons 1 to 13, and black lines represent introns. The positions of nucleotide changes are shown below the gene structure. The haplotypes are identified based on cDNA sequence diversity. W, the wild-type exon 10; H, the *helitron*-derived exon 10.

a

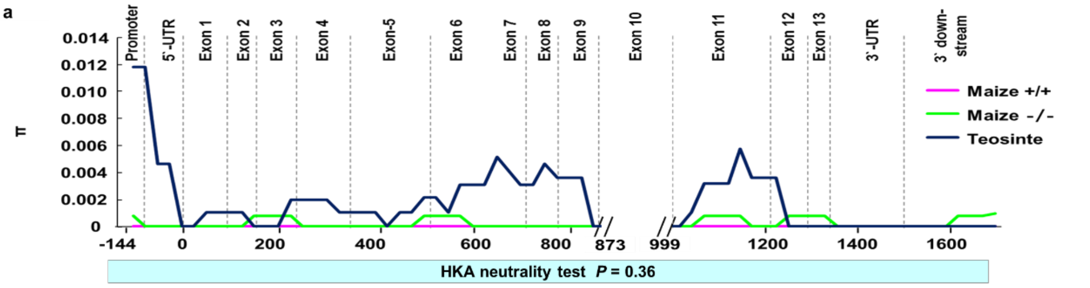

		Promoter	5`-UTR	Exon1	Exon2	Exon3	Exon4	Exon5	Exon6	Exon7	Exon8	Exon9	Exon11	Exon12	Exon13	3`UTR	3`down stream
Maize +/+	Tajima's D	0.00	0.00	0.00	0.00	0.00	0.00	0.00	0.00	0.00	0.00	0.00	0.00	0.00	0.00	0.00	0.00
Maize -/-		-1.16	0.00	0.00	0.00	-1.16	0.00	-0.17	-0.87	0.00	0.00	0.00	-0.51	-1.16	-0.58	0.00	-0.66
Teosinte		0.03	-0.31	-0.58	-1.16	-0.14	-0.71	-1.05	-1.23	-1.20	-0.80	0.31	-0.67	0.00	0.00	0.00	0.00
	π_+/π_-	0.00	NA	NA	NA	0.00	NA	0.00	0.00	NA	NA	NA	0.00	0.00	0.00	NA	0.00
	π_+/Π_T	0.00	0.00	0.00	0.00	0.00	0.00	0.00	0.00	0.00	0.00	0.00	0.00	NA	NA	NA	NA
	π_-/Π_T	0.07	0.00	0.00	0.00	1.55	0.00	0.10	0.25	0.00	0.00	0.00	0.12	NA	NA	NA	NA
	Length (bp)	58	86	87	60	87	108	173	112	81	63	102	211	75	54	137	209

b

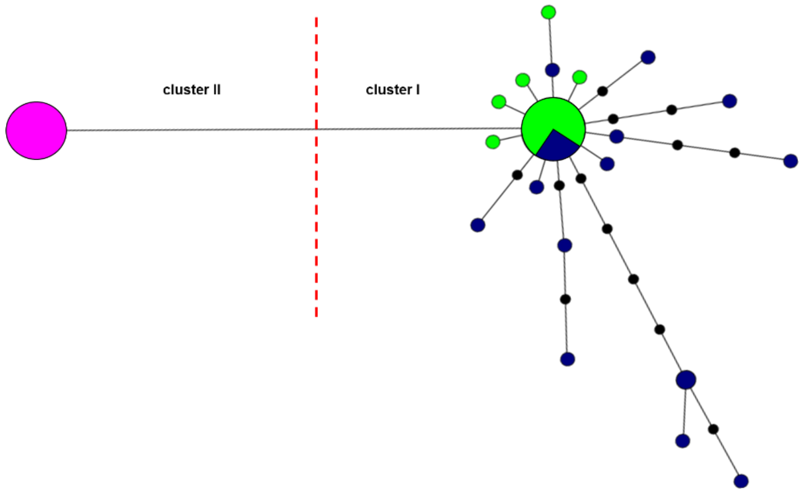

Supplementary Figure 14. The nucleotide diversities and minimum-spanning tree. a, Nucleotide diversity at the *ZmGDIα* locus. A panel of 24 *ZmGDIα-hel* maize lines (magenta, Maize+/+), 25 *ZmGDIα* maize lines (green, Maize-/-), and 19 teosinte entries (blue) were re-sequenced for their intact *ZmGDIα-hel* or *ZmGDIα* alleles.Nucleotide diversity (π) was separately calculated for the promoter, 5′-UTR, exon,3′-UTR, and 3′ downstream regions using a 100-bp sliding window with a 25-bp step.Results from the Tajima's *D* test and three π ratios, *ZmGDIα-hel* maize π_+) to *ZmGDIα* maize (π_-), π_+ to teosinte (π_T), and π_- to π_T, are shown. *P* value corresponds to HKA neutrality tests for the segment including 12 exons (excluding exon 10),144-bp promoter and 346-bp 3'-untranslated regions. **b,** A minimum-spanning tree forthe *ZmGDIα* locus. Each cycle represents one haplotype and the cycle size isproportional to the number of lines within the haplotype. Magenta, green, and bluecircles represent *ZmGDIα-hel* maize, *ZmGDIα* maize, and teosinte, respectively.

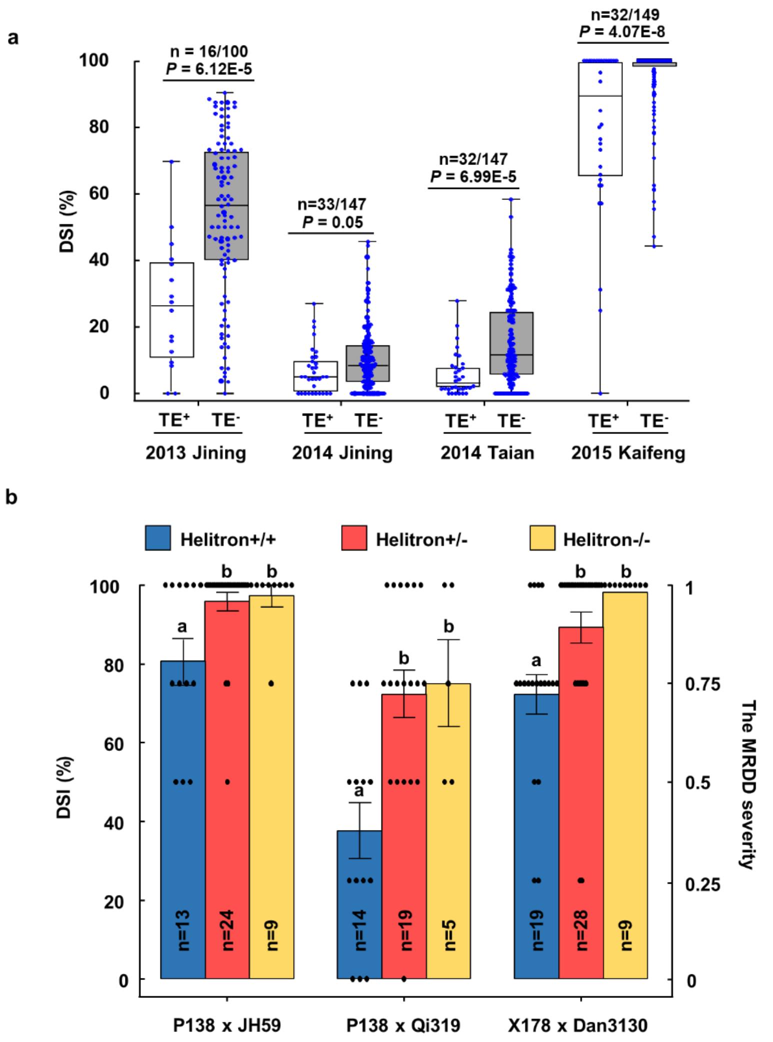

Supplementary Figure 15. The natural *ZmGDIα-hel* allele alleviates MRDD severity. a, The *helitron* TE insertion is tightly linked with MRDD resistance in a natural population. Despite the occurrence of light (Jining and Taian, 2014) or heavy (Jining, 2013; Kaifeng, 2015) MRDD in the field tests, 33 *ZmGDIα-hel* (TE^+) maize lines on average showed significantly lower disease severity index (DSI) than 153 *ZmGDIα* (TE^-) maize lines. Values are DSI means ± SEM. Statistical significance was determined using two-tailed Student's *t*-test. Each dot represents the DSI value of a single inbred line. Box edges represent quartiles, and the medians were shown with the central line in the boxes. **b,** Genetic contribution of *helitron* TE insertion to MRDD resistance in F_2 populations. Each F_2 plant was investigated for its polymorphic *helitron* TE and MRDD resistance. In each of three F_2 populations, DSI value was significantly lower in the *ZmGDIα-hel* homozygous (royalblue bars, *helitron* +/+) than *ZmGDIα* homozygous (yellow bars, *helitron* -/-) and heterozygous (tomato red bars, *helitron* +/-) plants. Values are means ± SEM. Multiple comparisons between genotypes were analyzed using SAS 9.1 PROC general linear model with Tukey's adjustment. Each dot represents the disease severity of a single F_2 plant. Source data underlying Supplementary Figure 15b are provided as a Source Data file.

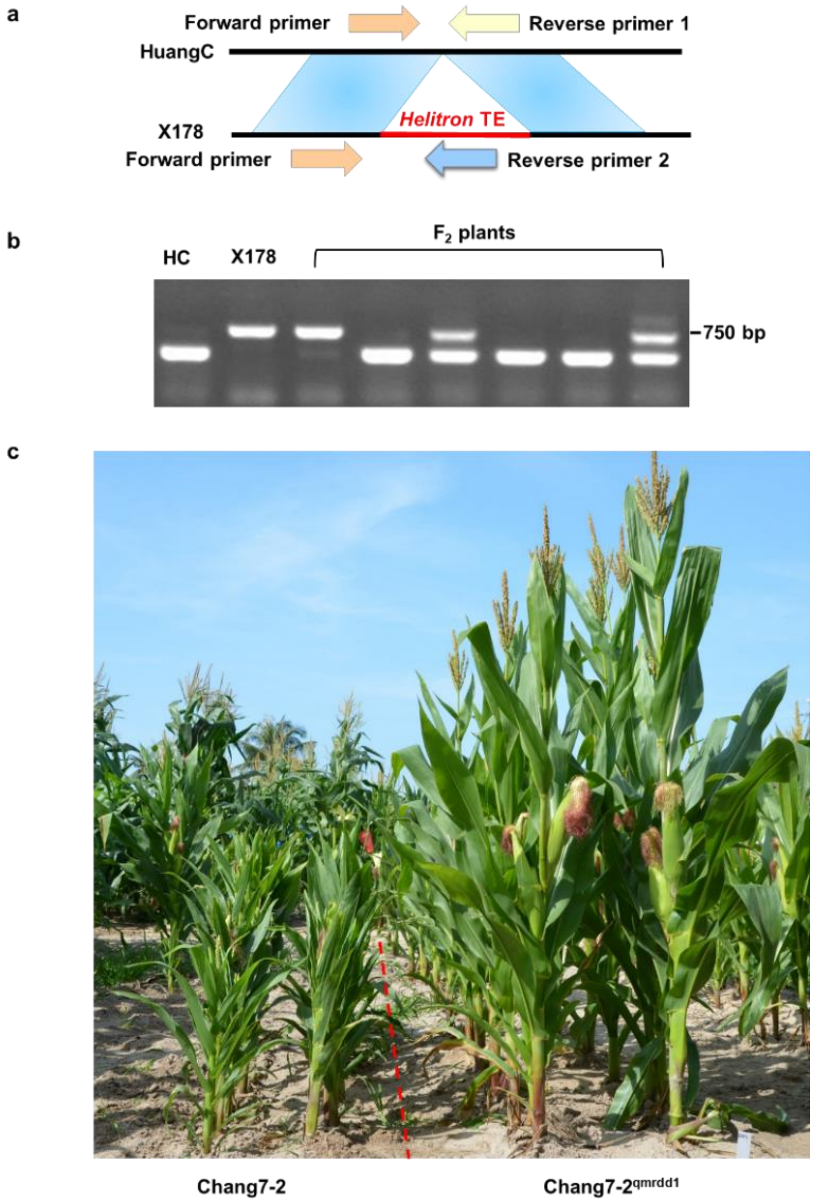

Supplementary Figure 16. Marker-assisted improvement of MRDD resistance. a, Primers designed based on the polymorphic *helitron* TE. One forward primer specific to the left-flanking region, and two separate reverse primer specific to the right-flanking region and *helitron* TE. **b,** Gel electrophoresis images of PCR products using triple primers. The mixed triple primers were used to amplify F_2 plants to distinguish three genotypes: *ZmGDIα-hel* homozygous (a single large band), heterozygous (two bands), and *ZmGDIα* homozygous (a single small band). The experiment was repeated three times independently, with the similar results. Source data are provided as a Source Data file. **c,** MRDD resistance in the field. The converted Chang7-2 with homozygous *ZmGDIα-hel* genotype showed dramatically higher resistance to MRDD than the original Chang7-2 with *ZmGDIα* homozygous genotype. Source data underlying Supplementary Figure 16b are provided as a Source Data file.

Supplementary Table 1. Input values used to perform the HKA test.

Loci[a]	S[b]	L[c]	N[d]	K[e]
AY104395	10	477	11	4.909
AY106816	39	532	13	37.615
AY107192	13	495	14	6.286
AY107248	21	538	14	7.286
AY111546	4	674	15	3.000
AY111711	26	528	15	13.200
ZmGDIα	6	1704	49	0.317

[a] Six neutral loci and ZmGDIα tested in this paper.

[b] Number of segregating sites.

[c] Number of total sites excluding gaps.

[d] Sample size.

[e] Average nucleotide difference.

Supplementary Reference

1. Wang F, et al. Improved method for assaying maize plant resistance to maize rough dwarf disease by artificial inoculation and real-time RT-PCR. *Eur J Plant Pathol* **116**:289-300 (2006).
