# Supplementary material for: A helitron-induced RabGDIα variant causes quantitative recessive resistance to maize rough dwarf disease
Source: Nat Commun. 2020 Jan 24;11:495. doi: 10.1038/s41467-020-14372-3 (PMC6981192; doi:10.1038/s41467-020-14372-3)
Supplement: Supplementary file 4 — Description of Additional Supplementary Files [file 41467_2020_14372_MOESM4_ESM.docx]

**Description of Additional Supplementary Files**

Supplementary Data 1
The list of primers used in the research;

Supplementary Data 2
Clustal alignment of 1145, B73, and HZ4 alleles at ZmGDIα

Supplementary Data 3
Clustal alignment of cDNAs of ZmGDIα and ZmGDIα-hel

Supplementary Data 4
Viral proteins interacting with ZmGDIα in co-IP-MS assay.

Supplementary Data 5
A survey of helitron TE insertion in a panel of 620 maize lines.

Supplementary Data 6
A survey of helitron TE insertion in a panel of 336 maize landraces.

Supplementary Data 7
A survey of helitron TE insertion in a panel of 184 teosinte entries.

Supplementary Data 8
Evaluation of MRDD resistance of 186 inbred lines in 2013, 2014, and 2016.
