# Supplementary material for: A helitron-induced RabGDIα variant causes quantitative recessive resistance to maize rough dwarf disease
Source: Nat Commun. 2020 Jan 24;11:495. doi: 10.1038/s41467-020-14372-3 (PMC6981192; doi:10.1038/s41467-020-14372-3)
Supplement: Supplementary file 6 — Supplementary Data 2 [file 41467_2020_14372_MOESM6_ESM.docx]

**Supplementary Data 2.**Clustal alignment of 1145, B73, and HZ4 alleles at *ZmGDIα.*

*ZmGDIα* start codon

1145 ATGGACGAGGAGTACGACGTGATCGTTCTGGGCACGGGGCTCAAGGAGTGCATCCTCAGC

B73 ATGGACGAGGAGTACGACGTGATCGTTCTGGGCACGGGGCTCAAGGAGTGCATCCTCAGC

HZ4 ATGGACGAGGAGTACGACGTGATCGTTCTGGGCACGGGGCTCAAGGAGTGCATCCTCAGC

************************************************************

1145 GGTCTCCTCTCTGTCGACGGCCTCAAGGTGAGCACCGACCACCCGCCTCCCGGTAACGGC

B73 GGTCTCCTCTCTGTCGACGGCCTCAAGGTGAGCACCGACCACCCGCCTCCCGGTAACGGC

HZ4 GGTCTCCTCTCTGTCGACGGCCTCAAGGTGAGCACCGACCACCCGCCTCCCGGTAACGGC

*************************************************************

1145 AATCGGACCTCACCCCAGTCCCCGAGTCCGATCTGCCACTCTCCCCCGCACGTACCCGGA

B73 AATCGGACCTCACCCCAGTCCCCGAGTCCGATCTGCCACTCTCCCCCGCACGTACCCGGA

HZ4 AATCGGACCTCACCCCAGTCCCCGAGTCCGATCTGCCACTCTCCCCCGCACGTACCCGGA

************************************************************

1145 CGCCGCATGTGGCCTGCGCGGTGGGTCTGCCGCGGTGCGTCGTGGTAGTAGATCAGGCGG

B73 CGCCGCATGTGGCCTGCGCGGTGGGTCTGCCGCGGTGCGTCGTGGTAGTAGATCAGGCGG

HZ4 CGCCGCATGTGGCCTGCGCGGTGGGTCTGCCGCGGTGCGTCGTGGTAGTAGATCAGGCGG

**************************************************************

1145 TGCTGGTCCGGCCCCTGTCTGATCCGGGACTGGCGCGTTCGGATGGGCGCTTGTCTAAGC

B73 TGCTGGTCCGGCCCCTGTCTGATCCGGGACTGGCGCGTTCGGATGGGCGCTTGTCTAAGC

HZ4 TGCTGGTCCGGCCCCTGTCTGATCCGGGACTGGCGCGTTCGGATGGGCGCTTGTCTAAGC

*************************************************************

1145 TGTCTACGCATCTGGCTCCTGGCATTGGTCCGGGGAGTTAACCAGTTGATGCAAACCATC

B73 TGTCTACGCATCTGGCTCCTGGCATTGGTCCGGGGAGTTAACCAGTTGATGCAAACCATC

HZ4 TGTCTACGCATCTGGCTCCTGGCATTGGTCCGGGGAGTTAACCAGTTGATGCAAACCATC

***********************************************************

1145 GTCTGCAAATGTTGCCACAACTTCTTGCATTCTGTGGACTGGGAAGATCAGGGCATGGTT

B73 GTCTGCAAATGTTGCCACAACTTCTTGCATTCTGTGGACTGGGAAGATCAGGGCATGGTT

HZ4 GTCTGCAAATGTTGCCACAACTTCTTGCATTCTGTGGACTGGGAAGATCAGGGCATGGTT

***********************************************************

1145 AGATTCTTTAATGAGGCAAACTAGTAACCTGGATTCTAGGGAGATTCACTGCTTCGAATT

B73 AGATTCTTTAATGAGGCAAACTAGTAACCTGGATTCTAGGGAGATTCACTGCTTCGAATT

HZ4 AGATTCTTTAATGAGGCAAACTAGTAACCTGGATTCTAGGGAGATTCACTGCTTCGAATT

**********************************************************

1145 TTGGTCTATCGTTTGTTCTTTGTTTCTGGGATGTAGAAGTGTTCCCATCCTGTAAGACGA

B73 TTGGTCTATCGTTTGTTCTTTGTTTCTGGGATGTAGAAGTGTTCCCATCCTGTAAGACGA

HZ4 TTGGTCTATCGTTTGTTCTTTGTTTCTGGGATGTAGAAGTGTTCCCATCCTGTAAGACGA

**********************************************************

1145 TAGATGGCATCTATATTCTGTCCTCTGATATCACTGCGGCGAGACAGGGGTAACTGAACG

B73 TAGATGGCATCTATATTCTGTCCTCTGATATCACTGCGGCGAGACAGGGGTAACTGAACG

HZ4 TAGATGGCATCTATATTCTGTCCTCTGATATCACTGCGGCGAGACAGGGGTAACTGAACG

***********************************************************

1145 GAGAGAGAAAGATGCTTAAGTTCAATAGTCAGCTTACTGCGTTAACTTGACCGATGATGG

B73 GAGAGAGAAAGATGCTTAAGTTCAATAGTCAGCTTACTGCGTTAACTTGACCGATGATGG

HZ4 GAGAGAGAAAGATGCTTAAGTTCAATAGTCAGCTTACTGCGTTAACTTGACCGATGATGG

***********************************************************

1145 ATTTTTTAAATTGATTTTGAAGAATTTTGAGTGACGCTTTTACAGGTTCTACACATGGAT

B73 ATTTTTTAAATTGATTTTGAAGAATTTTGAGTGACGCTTTTACAGGTTCTACACATGGAT

HZ4 ATTTTTTAAATTGATTTTGAAGAATTTTGAGTGACGCTTTTACAGGTTCTACACATGGAT

*********************************************************

1145 AGAAATGATTACTACGGAGGAGATTCCACCTCCCTAAACCTGAACCAGGCAAGAAGCTAT

B73 AGAAATGATTACTACGGAGGAGATTCCACCTCCCTAAACCTGAACCAGGCAAGAAGCTAT

HZ4 AGAAATGATTACTACGGAGGAGATTCCACCTCCCTAAACCTGAACCAGGCAAGAAGCTAT

***********************************************************

1145 AAATCTATGATTTTGGTTCTTTTTGAAACTGGAACATGTACTGAGTAATGTGTTCCATTT

B73 AAATCTATGATTTTGGTTCTTTTTGAAACTGGAACATGTACTGAGTAATGTGTTCCATTT

HZ4 AAATCTATGATTTTGGTTCTTTTTGAAACTGGAACATGTACTGAGTAATGTGTTCCATTT

*********************************************************

1145 TTTGTGTGTGCTGGCAACTCATTCTAATCCCTTTAATCACCCTTATCTTTTTTTTTGG-T

B73 TTTGTGTGTGCTGGCAACTCATTCTAATCCCTTTAATCACCCTTATCTTTTTTTTTGG-T

HZ4 TTTGTGTGTGCTGGCAACTCATTCTAATCCCTTTAATCACCCTTATCTTTTTTTTTTGGT

*********************************************************

1145 GAACAGCTCTGGAAGAGGTTTAGGGGGGAAGACAAGCCACCGGCACATCTAGGTGCAAGC

B73 GAACAGCTCTGGAAGAGGTTTAGGGGGGAAGACAAGCCACCGGCACATCTAGGTGCAAGC

HZ4 GAACAGCTCTGGAAGAGGTTTAGGGGGGAAGACAAGCCACCGGCACATCTAGGTGCAAGC

*************************************************************

1145 AGAGATTACAATGTAGACATGGTTCCAAAGGTGTGATAATTCCCGATCCCAATGGACCGT

B73 AGAGATTACAATGTAGACATGGTTCCAAAGGTGTGATAATTCCCGATCCCAATGGACCGT

HZ4 AGAGATTACAATGTAGACATGGTTCCAAAGGTGTGATAATTCCCGATCCCAATGGACCGT

**********************************************************

1145 ACCTAGCTATGTGCTTATTGTTTCTTGCCAAGGCCTCATCATACTTGGCAAATATATTGA

B73 ACCTAGCTATGTGCTTATTGTTTCTTGCCAAGGCCTCATCATACTTGGCAAATATATTGA

HZ4 ACCTAGCTATGTGCTTATTGTTTCTTGCCAAGGCCTCATCATACTTGGCAAATATATTGA

**********************************************************

1145 CTAAAAACATATATTTCCTTATGCTTGCACACTCATTAGTTCCTTACTTGTCTTATATAT

B73 CTAAAAACATATATTTCCTTATGCTTGCACACTCATTAGTTCCTTACTTGTCTTATATAT

HZ4 CTAAAAACATATATTTCCTTATGCTTGCACACTCATTAGTTCCTTACTTGTCTTATATAT

*******************************************************

1145 TTGTTACACTACCAGATACATCTTCGGACTGGTTAGTCTCATCCAGATAGCATTTACAAT

B73 TTGTTACACTACCAGATACATCTTCGGACTGGTTAGTCTCATCCAGATAGCATTTACAAT

HZ4 TTGTTACACTACCAGATACATCTTCGGACTGGTTAGTCTCATCCAGATAGCATTTACAAT

*********************************************************

1145 CAATATACTAGCTCAGTGCCCGTGCGTTGCAACGGGATCATATAATAGCTCGATAACTTA

B73 CAATATACTAGCTCAGTGCCCGTGCGTTGCAACGGGATCATATAATAGCTCGATAACTTA

HZ4 CAATATACTAGCTCAGTGCCCGTGCGTTGCAACGGGATCATATAATAGCTCGATAACTTA

**********************************************************

1145 TATATACAAATGTGTGTTATATTGTTATGAGATTGAGGACGACATCCACGAGCCGTCCAG

B73 TATATACAAATGTGTGTTATATTGTTATGAGATTGAGGACGACATCCACGAGCCGTCCAG

HZ4 TATATACAAATGTGTGTTATATTGTTATGAGATTGAGGACGACATCCACGAGCCGTCCAG

**********************************************************

1145 GATGACGTTGAAGAGAAACTCGGGCCTGTGACGCACGAACACTCACCTAGTAGTGTATTA

B73 GATGACGTTGAAGAGAAACTCGGGCCTGTGACGCACGAACACTCACCTAGTAGTGTATTA

HZ4 GATGACGTTGAAGAGAAACTCGGGCCTGTGACGCACGAACACTCACCTAGTAGTGTATTA

***********************************************************

1145 TACAAGATTTAACAAGACAATTTAAAGGCAAAAATTCAAGAATGATCTTTCATAAACTAC

B73 TACAAGATTTAACAAGACAATTTAAAGGCAAAAATTCAAGAATGATCTTTCATAAACTAC

HZ4 TACAAGATTTAACAAGACAATTTAAAGGCAAAAATTCAAGAATGATCTTTCATAAACTAC

*********************************************************

1145 TGTAAATTCATGACTAAAAGCTCACTGTAATTGACTTGCTTGAAGAACACAATAATACTA

B73 TGTAAATTCATGACTAAAAGCTCACTGTAATTGACTTGCTTGAAGAACACAATAATACTA

HZ4 TGTAAATTCATGACTAAAAGCTCACTGTAATTGACTTGCTTGAAGAACACAATAATACTA

*********************************************************

1145 CTGTGAATATGTGATACAACTTAATCACAGTAGTAAAATTACATCTGATGTTGTATTTGG

B73 CTGTGAATATGTGATACAACTTAATCACAGTAGTAAAATTACATCTGATGTTGTATTTGG

HZ4 CTGTGAATATGTGATACAACTTAATCACAGTAGTAAAATTACATCTGATGTTGTATTTGG

*********************************************************

1145 TAAAAAAATATTTTTTCAGAGTTCAGAGCCAACAACTAAACTCAGGTGTTACAAGGTACA

B73 TAAAAAAATATTTTTTCAGAGTTCAGAGCCAACAACTAAACTCAGGTGTTACAAGGTACA

HZ4 TAAAAAAATATTTTTTCAGAGTTCAGAGCCAACAACTAAACTCAGGTGTTACAAGGTACA

**********************************************************

1145 ACGAACCTTACTATTCAGATTATTATTAATATAAAAACTCAAGACAGGTGTAATCCTCAA

B73 ACGAACCTTACTATTCAGATTATTATTAATATAAAAACTCAAGACAGGTGTAATCCTCAA

HZ4 ACGAACCTTACTATTCAGATTATTATTAATATAAAAACTCAAGACAGGTGTAATCCTCAA

*********************************************************

1145 ACAATCATAAGCAATGTGTATAGGAATTTTAGACATAATGGTGCCTACAACATGCTTTAG

B73 ACAATCATAAGCAATGTGTATAGGAATTTTAGACATAATGGTGCCTACAACATGCTTTAG

HZ4 ACAATCATAAGCAATGTGTATAGGAATTTTAGACATAATGGTGCCTACAACATGCTTTAG

*********************************************************

1145 TATTCAGATTATTAACACAAACTCATGACATAATGACCAAAAAATATTAACACACAAACA

B73 TATTCAGATTATTAACACAAACTCATGACATAATGACCAAAAAATATTAACACACAAACA

HZ4 TATTCAGATTATTAACACAAACTCATGACATAATGACCAAAAAATATTAACACACAAACA

********************************************************

1145 TGGATATAATGTTGTTTAGGAATTTCAGACATATATAGAGGTCAAATAGAGGAATTTTAG

B73 TGGATATAATGTTGTTTAGGAATTTCAGACATATATAGAGGTCAAATAGAGGAATTTTAG

HZ4 TGGATATAATGTTGTTTAGGAATTTCAGACATATATAGAGGTCAAATAGAGGAATTTTAG

*********************************************************

1145 ACAATGAACAACTATCATCATCAATAGGCTAGAGTTCAAATTCTTAATATATGAACTTGA

B73 ACAATGAACAACTATCATCATCAATAGGCTAGAGTTCAAATTCTTAATATATGAACTTGA

HZ4 ACAATGAACAACTATCATCATCAATAGGCTAGAGTTCAAATTCTTAATATATGAACTTGA

*********************************************************

1145 CATATAATAGTGCCTCTAGCAGTGTCGCCTTAGTTTAAAAGCAGTAATATGGCATAAAGA

B73 CATATAATAGTGCCTCTAGCAGTGTCGCCTTAGTTTAAAAGCAGTAATATGGCATAAAGA

HZ4 CATATAATAGTGCCTCTAGCAGTGTCGCCTTAGTTTAAAAGCAGTAATATGGCATAAAGA

**********************************************************

1145 GGAGAGGAGAGCAGAGAAGAGAGGAAATAAACAAT-ATCTATTGCTTTCCATATAAACTC

B73 GGAGAGGAGAGCAGAGAAGAGAGGAAATAAACAAATATCTATTGCTTTCCATATAAACTC

HZ4 GGAGAGGAGAGCAGAGAAGAGAGGAAATAAACAAT-ATCTATTGCTTTCCATATAAACTC

**********************************: **********************

1145 ATAAAGTTATATATATTCCAATCCCATGGAGAGATGAGGGGAGGAGAGCTACAAAAACCA

B73 ATAAAGTTATATATATTCCAATCCCATGGAGAGATGAGGGGAGGAGAGCTACAAAAACCA

HZ4 ATAAAGTTATATATATTCCAATCCCATGGAGAGATGAGGGGAGGAGAGCTACAAAAACCA

**********************************************************

1145 ATCCTGTTCTGTTTCTCCTTTCAGGCCTAACCCTATGCAGCAGCATATTCATCTAAGAAA

B73 ATCCTGTTCTGTTTCTCCTTTCAGGCCTAACCCTATGCAGCAGCATATTCATCTAAGAAA

HZ4 ATCCTGTTCTGTTTCTCCTTTCAGGCCTAACCCTATGCAGCAGCATATTCATCTAAGAAA

**********************************************************

1145 CCAACAACCTGTAACATATCCCTTATTATCCTACCCATTACAAACTTGTCATTAAAATTA

B73 CCAACAACCTGTAACATATCCCTTATTATCCTACCCATTACAAACTTGTCATTAAAATTA

HZ4 CCAACAACCTGTAACATATCCCTTATTATCCTACCCATTACAAACTTGTCATTAAAATTA

********************************************************

1145 AATTCAGTACTAACTAATATTATCTCGATCAATCTTCAAGGACATCAAGCTCGATTTTGC

B73 AATTCAGTACTAACTAATATTATCTCGATCAATCTTCAAGGACATCAAGCTCGATTTTGC

HZ4 AATTCAGTACTAACTAATATTATCTCGATCAATCTTCAAGGACATCAAGCTCGATTTTGC

*********************************************************

1145 TATGCTACAGAGAATGGTGCATGTGGACCCTTCTTCCTTATATGCATCAGGTGGTCTAAA

B73 TATGCTACAGAGAATGGTGCATGTGGACCCTTCTTCCTTATATGCATCAGGTGGTCTAAA

HZ4 TATGCTACAGAGAATGGTGCATGTGGACCCTTCTTCCTTATATGCATCAGGTGGTCTAAA

**********************************************************

1145 GTTGGCTTTAACCTTTAGCCCACCTT-ACATTAGAGATTTGGACATCAAAAGAAAGCTAA

B73 GTTGGCTTTAACCTTTAGCCCACCTTTTCATTAGAGATTTGGACATCAAAAGAAAGCTAA

HZ4 GTTGGCTTTAACCTTTAGCCCACCTT-ACATTAGAGATTTGGACATCAAAAGAAAGCTAA

************************* *****************************

1145 AACATTCTAAACCTTTTAGATCTCTAAGTTTAGAAGAGGGAGGCAAACATGCCCTAGAAT

B73 AACATTCTAAACCTTTTAGATCTCTAAGTTTAGAAGAGGGAGGCAAACATGCCCTAGAAT

HZ4 AACATTCTAAACCTTTTAGATCTCTAAGTTTAGAAGAGGGAGGCAAACATGCCCTAGAAT

**********************************************************

1145 ATTGGAGATCGCAGTTGCTGCTATCACCAAGTAACACACTAAGATATCGAAGACCACAAC

B73 ATTGGAGATCGCAGTTGCTGCTATCACCAAGTAACACACTAAGATATCGAAGACCACAAC

HZ4 ATTGGAGATCGCAGTTGCTGCTATCACCAAGTAACACACTAAGATATCGAAGACCACAAC

***********************************************************

1145 TGCTGCTATCACCGTGCTTTTACTTCTACCTTAACAACTAAGATCTCAATCCATCTTAAT

B73 TGCTGCTATCACCGTGCTTTTACTTCTACCTTAACAACTAAGATCTCAATCCATCTTAAT

HZ4 TGCTGCTATCACCGTGCTTTTACTTCTACCTTAACAACTAAGATCTCAATCCATCTTAAT

************************************************************

1145 TTGTCCAACCGAACTCTGAATGAAAAAAAGGAATCGCATTTCAGGAATGATTGACGAGTT

B73 TTGTCCAACCGAACTCTGAATGAAAAAAAGGAATCGCATTTCAGGAATGATTGACGAGTT

HZ4 TTGTCCAACCGAACTCTGAATGAAAAAAAGGAATCGCATTTCAGGAATGATTGACGAGTT

***********************************************************

1145 AGCACAATGCAGGTTTAGAACCTTTGGTTATTTCTAAAATAAAGACTCGCATGTATGGCT

B73 AGCACAATGCAGGTTTAGAACCTTTGGTTATTTCTAAAATAAAGACTCGCATGTATGGCT

HZ4 AGCACAATGCAGGTTTAGAACCTTTGGTTATTTCTAAAATAAAGACTCGCATGTATGGCT

**********************************************************

1145 ATAACAATTTATACTTTTAAAAATGGATACTTATTGAGACCTGATAACCACATTTAGCTA

B73 ATAACAATTTATACTTTTAAAAATGGATACTTATTGAGACCTGATAACCACATTTAGCTA

HZ4 ATAACAATTTATACTTTTAAAAATGGATACTTATTGAGACCTGATAACCACATTTAGCTA

********************************************************

1145 GCTTAGCAGGAGGGAGCATGCTAACTCATAATGCCTTGTAATTACTGAATACTGATGAAC

B73 GCTTAGCAGGAGGGAGCATGCTAACTCATAATGCCTTGTAATTACTGAATACTGATGAAC

HZ4 GCTTAGCAGGAGGGAGCATGCTAACTCATAATGCCTTGTAATTACTGAATACTGATGAAC

**********************************************************

1145 CAGCGGAGCCATACCTCATTAACATCCTCAAGCAGCTCTGACAAACAAATTAGGGGACAT

B73 CAGCGGAGCCATACCTCATTAACATCCTCAAGCAGCTCTGACAAACAAATTAGGGGACAT

HZ4 CAGCGGAGCCATACCTCATTAACATCCTCAAGCAGCTCTGACAAACAAATTAGGGGACAT

***********************************************************

1145 GACAAATAAATTAGGGGACACTACCTCTGCGTATCTGCACAAGGGCCCAAGAGAGGTGGG

B73 GACAAATAAATTAGGGGACACTACCTCTGCGTATCTGCACAAGGGCCCAAGAGAGGTGGG

HZ4 GACAAATAAATTAGGGGACACTACCTCTGCGTATCTGCACAAGGGCCCAAGAGAGGTGGG

************************************************************

1145 GCCCGGCCATAGGAACGGAGCTCTCAGCTCCCACACCGTGCACCAGTGAATGCGAGCAGC

B73 GCCCGGCCATAGGAACGGAGCTCTCAGCTCCCACACCGTGCACCAGTGAATGCGAGCAGC

HZ4 GCCCGGCCATAGGAACGGAGCTCTCAGCTCCCACACCGTGCACCAGTGAATGCGAGCAGC

*************************************************************

1145 TGGTCTGGCCTTCCAGTAATCCGATCTGCATACAGTGTGATCCAGGATTCACGTGGATAA

B73 TGGTCTGGCCTTCCAGTAATCCGATCTGCATACAGTGTGATCCAGGATTCACGTGGATAA

HZ4 TGGTCTGGCCTTCCAGTAATCCGATCTGCATACAGTGTGATCCAGGATTCACGTGGATAA

***********************************************************

1145 GGATGAAATCAAGAGAGCGCTAGCTAGGATCACACGCCAATAGCACAAGGAAGGAAGGAA

B73 GGATGAAATCAAGAGAGCGCTAGCTAGGATCACACGCCAATAGCACAAGGAAGGAAGGAA

HZ4 GGATGAAATCAAGAGAGCGCTAGCTAGGATCACACGCCAATAGCACAAGGAAGGAAGGAA

************************************************************

1145 TGAGCGAACGAACCTTGAGGAAAACATCGCCGACGGGCTTCTTTGTGTGGCTCTCTGTCG

B73 TGAGCGAACGAACCTTGAGGAAAACATCGCCGACGGGCTTCTTTGTGTGGCTCTCTGTCG

HZ4 TGAGCGAACGAACCTTGAGGAAAACATCGCCGACGGGCTTCTTTGTGTGGCTCTCTGTCG

************************************************************

1145 TTGGTCGCAACCCCCGCCACTGCCCTCACTCGTCGCACATCGCGTTGTGGTCACGGTCGG

B73 TTGGTCGCAACCCCCGCCACTGCCCTCACTCGTCGCACATCGCGTTGTGGTCACGGTCGG

HZ4 TTGGTCGCAACCCCCGCCACTGCCCTCACTCGTCGCACATCGCGTTGTGGTCACGGTCGG

************************************************************

1145 GGCTGCCCTTCCGTCCGGTCGACCGGTTGAGCATGGGGCGATCAAGCAGGACTCGAGGAG

B73 GGCTGCCCTTCCGTCCGGTCGACCGGTTGAGCATGGGGCGATCAAGCAGGACTCGAGGAG

HZ4 GGCTGCCCTTCCGTCCGGTCGACCGGTTGAGCATGGGGCGATCAAGCAGGACTCGAGGAG

*************************************************************

1145 GAGGTAGCGGATTTATGGCGACGGATCGGGGTAGCGGGGCCGAGTGTGGGTTGTTGGCTT

B73 GAGGTAGCGGATTTATGGCGACGGATCGGGGTAGCGGGGCCGAGTGTGGGTTGTTGGCTT

HZ4 GAGGTAGCGGATTTATGGCGACGGATCGGGGTAGCGGGGCCGAGTGTGGGTTGTTGGCTT

*************************************************************

1145 GTTGCGCCCGTGGGCGTGGCAGGCGGGGGGTGGGGGCGGGCGGAGGGATCATGAGCTAGA

B73 GTTGCGCCCGTGGGCGTGGCAGGCGGGGGGTGGGGGCGGGCGGAGGGATCATGAGCTAGA

HZ4 GTTGCGCCCGTGGGCGTGGCAGGCGGGGGGTGGGGGCGGGCGGAGGGATCATGAGCTAGA

***************************************************************

1145 GGGGGAGATCGTGGAGGGAGTTGGCAGTTTGGAGGAAGATCATGGGGGATTCCTTTGCGT

B73 GGGGGAGATCGTGGAGGGAGTTGGCAGTTTGGAGGAAGATCATGGGGGATTCCTTTGCGT

HZ4 GGGGGAGATCGTGGAGGGAGTTGGCAGTTTGGAGGAAGATCATGGGGGATTCCTTTGCGT

*************************************************************

1145 GGGCGTCTGGGAGTGAGCGGGTGCTCCTAAGATGGCTGAGATCGACGATGAACATGAGAG

B73 GGGCGTCTGGGAGTGAGCGGGTGCTCCTAAGATGGCTGAGATCGACGATGAACATGAGAG

HZ4 GGGCGTCTGGGAGTGAGCGGGTGCTCCTAAGATGGCTGAGATCGACGATGAACATGAGAG

*************************************************************

1145 CACGGGACGGAGACGGCGCTAGCAGGAAGGGCTAGAGGGCGAGGGCTCGCTAGGCAGAGA

B73 CACGGGACGGAGACGGCGCTAGCAGGAAGGGCTAGAGGGCGAGGGCTCGCTAGGCAGAGA

HZ4 CACGGGACGGAGACGGCGCTAGCAGGAAGGGCTAGAGGGCGAGGGCTCGCTAGGCAGAGA

************************************************************

1145 AAGCAAGGGAGACTGACCCTAAAGCCAAGCTGGGTCAGGGTAAGAAGCGTGCGG-CCTCC

B73 AAGCAAGGGAGACTGACCCTAAAGCCAAGCTGGGTCAGGGTAAGAAGCGTGCGGGCCTCC

HZ4 AAGCAAGGGAGACTGACCCTAAAGCCAAGCTGGGTCAGGGTAAGAAGCGTGCGG-CCTCC

******************************************************* *****

1145 TAGCAGCTACTTGGGCCGCACCAAAGAAACACAGCCCACGCGACCTATGTCGTCATCACG

B73 TAGCAGCTACTTGGGCCGCACCAAAGAAACACAGCCCACGCGACCTATGTCGTCATCACG

HZ4 TAGCAGCTACTTGGGCCGCACCAAAGAAACACAGCCCACACGACCTATGTCGTCATCACG

*************************************** ********************

1145 GTTTGCACATTTTTATGACCATACCGGCCAGCCAACATAGCTTCTACCAGCTTATAAGCC

B73 GTTTGCACATTTTTATGACCATACCGGCCAGCCAACATAGCTTCTACCAGCTTATAAGCC

HZ4 GTTTGCACATTTTTATGACCATACCGGCCAGCCAACATAGCTTCTACCAGCTTATAAGCC

**********************************************************

1145 TTCCTTCTTTAGAGAAAGCAATCATGGTCAAATGTTCCTGCTAGTGTCAAATTGAGGCAA

B73 TTCCTTCTTTAGAGAAAGCAATCATGGTCAAATGTTCCTGCTAGTGTCAAATTGAGGCAA

HZ4 TTCCTTCTTTAGAGAAAGCAATCATGGTCAAATGTTCCTGCTAGTGTCAAATTGAGGCAA

***********************************************************

1145 CTTTTTGCGCGAGCAGACGTGGGTCCGGTGTGTGCAAAACTAATGGTGGCTGTCACGTGG

B73 CTTTTTGCGCGAGCAGACGTGGGTCCGGTGTGTGCAAAACTAATGGTGGCTGTCACGTGG

HZ4 CTTTTTGCGCGAGCAGACGTGGGTCCGGTGTGTGCAAAACTAATGGTGGCTGTCACGTGG

*************************************************************

1145 GCGTCCGTAGTCTGCAATGTGGGCCCAACGTCCGGAGTGTGTGATGGCTTGCACGATAGG

B73 GCGTCCGTAGTCTGCAATGTGGGCCCAACGTCCGGAGTGTGTGATGGCTTGCACGATAGG

HZ4 GCGTCCGTAGTCTGCAATGTGGGCCCAACGTCCGGAGTGTGTGATGGCTTGCACGATAGG

************************************************************

1145 AATGGCTGAATGGGAATGGGGGATTTTGTCATATGGGACCGGAGTGCCCCAAGTCAGGAG

B73 AATGGCTGAATGGGAATGGGGGATTTTGTCATATGGGACCGGAGTGCCCCAAGTCAGGAG

HZ4 AATGGCTGAATGGGAATGGGGGATTTTGTCATATGGGACCGGAGTGCCCCAAGTCAGGAG

************************************************************

1145 TTGCTCGACGTCCCG-CGTCACAGAATGTGATGGTTTGCCGGACAACAGCGGTGGAGGAG

B73 TTGCTCGACGTCCCCGCGTCACAGAATGTGATGGTTTGCCGGACAACAGCGGTGGAGGAG

HZ4 TTGCTCGACGTCCCG-CGTCACAGAATGTGATGGTTTGCCGGACAACAGCGGTGGAGGAG

************** ********************************************

1145 CCATTATGGCTTAGTGTGGCATCAGCCATACCTTACTTTTAATAACTCTCATATCTTCTA

B73 CCATTATGGCTTAGTGTGGCATCAGCCATACCTTACTTTTAATAACTCTCATATCTTCTA

HZ4 CCATTATGGCTTAGTGTGGCATCAGCCATACCTTACTTTTAATAACTCTCATATCTTCTA

*********************************************************

1145 CTATAACATCAAAATAAGATGTGGTGTGGTGGATATGATGTCTCTAATAGATGTTTTCTT

B73 CTATACCATCAAAATTCGATGTGGTGTGGTGGATATGCCGTCTCTCCTAGATGTTTTCAT

HZ4 CTATAACATCAAAATAAGATGTGGTGTGGTGGATATGATGTCTCTAATAGATGTTTTCTT

***** ********* ******************** ****** *********** *

1145 AACATATAGTTGATGGAGATACAATTCATTCATCAAATGAATAT----------------

B73 ACCCACTTGCTGATATATATATATAT--------ATATATATAT----------------

HZ4 AACATATAGTTGATGGAGATACAATTCATTCATCAAATGAATATTTATATATATATATAT

* * *:* **** * *** * * * ** ****

1145 ------------------------------------------------------------

B73 ------------------------------------------------------------

HZ4 ATATATATATATATATATATATATATATATATATATATATATATATATATATATATATAT

1145 ------------------------------------------------------------

B73 ------------------------------------------------------------

HZ4 ATATATATATATATATATATATATATATATATATATATATATATATATATATATATATAT

1145 ------------------------------------------------------------

B73 ------------------------------------------------------------

HZ4 ATATATATATATATATATATATATATATATATATATATATATATATATATATATATATAT

1145 ------------------------------------------------------------

B73 ------------------------------------------------------------

HZ4 ATATATATATATATATATATATATATATATATATATATATATATATATATATATATATAT

1145 ------------------------------------------------------------

B73 ------------------------------------------------------------

HZ4 ATATATATATATATATATATATATATATATATATATATATATATATATATATATATATAT

1145 ------------------------------------------------------------

B73 ------------------------------------------------------------

HZ4 ATATATATATATATATATATATATATATATATATATATATATATATATATATATATATAT

1145 ------------------------------------------ATATATATATATATATAT

B73 ------------------------------------------ATATATATATATATATAT

HZ4 ATATATATATATATATATATATATATATATATATATATATATATATATATATATATATAT

******************

1145 ATATATATATATATATATATATATATATATATATATATATATATATATATATATATATAT

B73 ATATATATATATATATATATATATATATATATATATATATATATATATATATATATATAT

HZ4 ATATATATATATATATATATATATATATATATATATATATATATATATATATATATATAT

*************************************************

1145 ATATAGCTGAATGCCCGTGCGTTGCAACGGAAATATATAATACCCGTATACTACGATAAC

B73 ATATAGCTGAATGCCCGTGCGTTGCAACGGAAATATATAATACCCGTATACTACGATAAC

HZ4 ATATAGCTGAATGCCCGTGCGTTGCAACGGAAATATATAATACCCGTATACTACGATAAC

*********************************************************

1145 TTATATACAAAATATGTGTTATACCGTTATGAGAAAATGTTTCATAATCAATTTGTGATT

B73 TTATATACAAAATATGTGTTATACCGTTATGAGAAAATGTTTCATAATCAATTTGTGATT

HZ4 TTATATACAAAATATGTGTTATACCGTTATGAGAAAATGTTTCATAATCAATTTGTGATT

*******************************************************

1145 CTGGCCATACATAAATTTTGTTATTTATAATCTATCTATTTCACCACTACATTGCAACCA

B73 CTGGCCATACATAAATTTTGTTATTTATAATCTATCTATTTCACCACTACATTGCAACCA

HZ4 CTGGCCATACATAAATTTTGTTATTTATAATCTATCTATTTCACCACTACATTGCAACCA

********************************************************

1145 TCAGTATCATGCAGACTTCGATATATGTCACGATTTGCATGGCCTCATTATTGGAGAGCA

B73 TCAGTATCATGCAGACTTCGATATATGTCACGATTTGCATGGCCTCATTATTGGAGAGCA

HZ4 TCAGTATCATGCAGACTTCGATATATGTCACGATTTGCATGGCCTCATTATTGGAGAGCA

**********************************************************

1145 CGTTTCACACATACCGGAAGAAATTCGCTCGTACATCGTTAGTCATCGGACACGTACCAC

B73 CGTTTCACACATACCGGAAGAAATTCGCTCGTACATCGTTAGTCATCGGACACGTACCAC

HZ4 CGTTTCACACATACCGGAAGAAATTCGCTCGTACATCGTTAGTCATCGGACACGTACCAC

*************************************************************

1145 CTTACACTTTTCCTTAAACAAAAAGGTAAGTGTGTGTGTTTGCAAGACTAATGGTCAAAC

B73 CTTACACTTTTCCTTAAACAAAAAGGTAAGTGTGTGTGTTTGCAAGACTAATGGTCAAAC

HZ4 CTTACACTTTTCCTTAAACAAAAAGGTAAGTGTGTGTGTTTGCAAGACTAATGGTCAAAC

*************************************************************

1145 ATTGTATAACCATAAAGTTAGAATACTATATTAATATATTAAATAAATTAATCCAATAGA

B73 ATTGTATAACCATAAAGTTAGAATACTATATTAATATATTAAATAAATTAATCCAATAGA

HZ4 ATTGTATAACCATAAAGTTAGAATACTATATTAATATATTAAATAAATTAATCCAATAGA

*********************************************************

1145 CATAGATTAACTCAATTAACATAGGATAAATAAATATCTAATTATAAAAGTATGAAACAT

B73 CATAGATTAACTCAATTAACATAGGATAAATAAATATCTAATTATAAAAGTATGAAACAT

HZ4 CATAGATTAACTCAATTAACATAGGATAAATAAATATCTAATTATAAAAGTATGAAACAT

**********************************************************

1145 GGTATAATCAATGTTGTTACTGCGTAGTAAATATTCATACGAGACTAACACAACTGGAAC

B73 GGTATAATCAATGTTGTTACTGCGTAGTAAATATTCATACGAGACTAACACAACTGGAAC

HZ4 GGTATAATCAATGTTGTTACTGCGTAGTAAATATTCATACGAGACTAACACAACTGGAAC

************************************************************

1145 GGTTCAATTTAGAATTAAAATGGGGAAGTTACGAATTTCCAAAGTTTCTATGTATTTAAT

B73 GGTTCAATTTAGAATTAAAATGGGGAAGTTACGAATTTCCAAAGTTTCTATGTATTTAAT

HZ4 GGTTCAATTTAGAATTAAAATGGGGAAGTTACGAATTTCCAAAGTTTCTATGTATTTAAT

***********************************************************

1145 ATAGGATTAATTAGGAAATCAGTTTTATTGTTGTTTTCATGACAAAACAGAGGTATTATG

B73 ATAGGATTAATTAGGAAATCAGTTTTATTGTTGTTTTCATGACAAAACAGAGGTATTATG

HZ4 ATAGGATTAATTAGGAAATCAGTTTTATTGTTGTTTTCATGACAAAACAGAGGTATTATG

***********************************************************

1145 TGATAAAAAATAATATTATAGAATTATAGAAATTGGAATAAACTCATTTGGATTTAATAT

B73 TGATAAAAAATAATATTATAGAATTATAGAAATTGGAATAAACTCATTTGGATTTAATAT

HZ4 TGATAAAAAATAATATTATAGAATTATAGAAATTGGAATAAACTCATTTGGATTTAATAT

*********************************************************

1145 GAAATTTCCATGAATTAAATGAGTTTCTGCAATTATTTTTAAACTAAAAATTAATTTCTA

B73 GAAATTTCCATGAATTAAATGAGTTTCTGCAATTATTTTTAAACTAAAAATTAATTTCTA

HZ4 GAAATTTCCATGAATTAAATGAGTTTCTGCAATTATTTTTAAACTAAAAATTAATTTCTA

**********************************************************

1145 AACTCCTTTTCTCTATTTTCTTTATTTCCTGGACCGCGTCCCAAATTCCAGAAAGATCAG

B73 AACTCCTTTTCTCTATTTTCTTTATTTCCTGGACTGCGTCCCAAATTCCAGAAAGATCAG

HZ4 AACTCCTTTTCTCTATTTTCTTTATTTCCTGGACCGCGTCCCAAATTCCAGAAAGATCAG

********************************** **************************

1145 GGGCCAAGCTATAAATGTTTTTCTAGACTCAGTGAGCATACAGAGTGAACGACAGGTTAA

B73 GGGCCAAGCTATAAATGTTTTTCTAGACTCAGTGAGCATACAGAGTGAACGACGGGTTAA

HZ4 GGGCCAAGCTATAAATGTTTTTCTAGACTCAGTGAGCATACAGAGTGAACGACGGGTTAA

****************************************************** *******

1145 ACACAAATAAAGTCTTAGTTGAAGTTGTACACAATTCAATATGAGTCACGTTGGTTAGAG

B73 ACACAAATAAAGTCTTAGTTGAAGTTGTACACAATTCAATATGAGTCACGTTGGTTAGAG

HZ4 ACACAAATAAAGTCTTAGTTGAAGTTGTACACAATTCAATATGAGTCACGTTGGTTAGAG

************************************************************

1145 GGTGGAAGGCTAGACATTCTACTCAATAGATCTAAGGTTCGATCCCTGAGTAGCACAGTT

B73 GGTGGAAGGCTAGACATTCTACTCAATAGATCTAAGGTTCGATCCCTGAGTAGCACAGTT

HZ4 GGTGGAAGGCTAGACATTCTACTCAATAGATCTAAGGTTCGATCCCTGAGTAGCACAGTT

*************************************************************

1145 TAATTTTTTTTCTGCGCCCGGAACGGGGGAGGCAGAACGGCAGACTACTCTTACCTTCTT

B73 TAATTTTTTTTCTGCGCCCGGAACGGGGGAGGCAGAACGGCAGACTACTCTTACCTTCTT

HZ4 TAATTTTTTTTCTGCGCCCGGAACGGGGGAGGCAGAACGGCAGACTACTCTTACCTTCTT

**************************************************************

1145 AATAAGTAGTATAGATGTTTATGTTAATTTCTTGATGATAAAGTATGTGTATATCAAACT

B73 AATAAGTAGTATAGATGTTTATGTTAATTTCTTGATGATAAAGTATGTGTATATCAAACT

HZ4 AATAAGTAGTATAGATGTTTATGTTAATTTCTTGATGATAAAGTATGTGTATATCAAACT

**********************************************************

1145 CTCCTCTCTGACCAGTTTATGATGGCAAACGGGACTTTGGTTCGCACTCTCATTCACACT

B73 CTCCTCTCTGACCAGTTTATGATGGCAAACGGGACTTTGGTTCGCACTCTCATTCACACT

HZ4 CTCCTCTCTGACCAGTTTATGATGGCAAACGGGACTTTGGTTCGCACTCTCATTCACACT

*************************************************************

1145 GATGTGACAAAATATTTGTCATTCAAAGCTGTTGATGGAAGCTATGTCTTCAGCAAACGG

B73 GATGTGACAAAATATTTGTCATTCAAAGCTGTTGATGGAAGCTATGTCTTCAGCAAACGG

HZ4 GATGTGACAAAATATTTGTCATTCAAAGCTGTTGATGGAAGCTATGTCTTCAGCAAACGG

************************************************************

1145 AAGGTAATTTCTTAAGTTTACACTCATCCATCCTAGTTCTATCTTGTAAGTTCAGCTTTG

B73 AAGGTAATTTCTTAAGTTTACACTCATCCATCCTAGTTCTATCTTGTAAGTTCAGCTTTG

HZ4 AAGGTAATTTCTTAAGTTTACACTCATCCATCCTAGTTCTATCTTGTAAGTTCAGCTTTG

************************************************************

1145 CTGGATTTCTCACAAGTGGTTTCTATATTGTAATCATGCCGCCTTATTGCATTTTCATCA

B73 CTGGATTTCTCACAAGTGGTTTCTATATTGTAATCATGCCGCCTTATTGCATTTTCATCA

HZ4 CTGGATTTCTCACAAGTGGTTTCTATATTGTAATCATGCCGCCTTATTGCATTTTCATCA

***********************************************************

1145 GATTCACAAGGTTCCTGCCACCGATATGGAGGCTCTAAAATCTCCTTTGATGGGTCTATT

B73 GATTCACAAGGTTCCTGCCACCGATATGGAGGCTCTAAAATCTCCTTTGATGGGTCTATT

HZ4 GATTCACAAGGTTCCTGCCACCGATATGGAGGCTCTAAAATCTCCTTTGATGGGTCTATT

*************************************************************

1145 TGAGAAACGTAGAGCAAGGAACTTTTTTGTTTACGTCCAAAATTACAATGAAGCTGATCC

B73 TGAGAAACGTAGAGCAAGGAACTTTTTTGTTTACGTCCAAAATTACAATGAAGCTGATCC

HZ4 TGAGAAACGTAGAGCAAGGAACTTTTTTGTTTACGTCCAAAATTACAATGAAGCTGATCC

*************************************************************

1145 AGTGACACATCAGGGGTTGGACCTCACAAGGATTACAACTAGAGAATTGATTTTGTGAGT

B73 AGTGACACATCAGGGGTTGGACCTCACAAGGATTACAACTAGAGAATTGATTTTGTGAGT

HZ4 AGTGACACATCAGGGGTTGGACCTCACAAGGATTACAACTAGAGAATTGATTTTGTGAGT

**************************************************************

1145 ATCTTGACCATCCTAGCATCGTAATACTTACTGGCATTGTTGTTCCTTGTTTGCCATGTT

B73 ATCTTGACCATCCTAGCATCGTAATACTTACTGGCATTGTTGTTCCTTGTTTGCCATGTT

HZ4 ATCTTGACCATCCTAGCATCGTAATACTTACTGGCATTGTTGTTCCTTGTTTGCCATGTT

************************************************************

1145 ATTATGTGTTGTATGATTCTTTGTATCACTCCACCTCATGTTGCTGCATGGATGTTGTAT

B73 ATTATGTGTTGTATGATTCTTTGTATCACTCCACCTCATGTTGCTGCATGGATGTTGTAT

HZ4 ATTATGTGTTGTATGATTCTTTGTATCACTCCACCTCATGTTGCTGCATGGATGTTGTAT

***********************************************************

1145 GATTCTTCACCTACTTTTGCCTCTTTCTGCTTTCGTATAGTATGATAATATTTTGTGCAT

B73 GATTCTTCACCTACTTTTGCCTCTTTCTGCTTTCGTATAGTATGATAATATTTTGTGCAT

HZ4 GATTCTTCACCTACTTTTGCCTCTTTCTGCTTTCGTATAGTATGATAATATTTTGTGCAT

***********************************************************

1145 CAATAATGTACACAAGTTAATTGTTGTAATGTTATATTGATGAACAGATAAACAAAACTG

B73 CAATAATGTACACAAGTTAATTGTTGTAATGTTATATTGATGAACAGATAAACAAAACTG

HZ4 CAATAATGTACACAAGTTAATTGTTGTAATGTTATATTGATGAACAGATAAACAAAACTG

***********************************************************

1145 CTATAACCGTTCAACTTCTAAATGACACTTTTTCTTAAAGCTGTAAAGAGGATGTGTAAT

B73 CTATAACCGTTCAACTTCTAAATGACACTTTTTCTTAAAGCTGTAAAGAGGATGTGTAAT

HZ4 CTATAACCGTTCAACTTCTAAATGACACTTTTTCTTAAAGCTGTAAAGAGGATGTGTAAT

************************************************************

1145 ACTTGGCATGTCCTCCTTTTGCATTATAAGTAACCTTATAAGTCATTTTGTTTTCAGGAA

B73 ACTTGGCATGTCCTCCTTTTGCATTATAAGTAACCTTATAAGTCATTTTGTTTTCAGGAA

HZ4 ACTTGGCATGTCCTCCTTTTGCATTATAAGTAACCTTATAAGTCATTTTGTTTTCAGGAA

************************************************************

1145 ACATGGATTGAGTGATGACACTGTGGATTTTATTGGCCATGCACTCGCTCTGCACAGGGA

B73 ACATGGATTGAGTGATGACACTGTGGATTTTATTGGCCATGCACTCGCTCTGCACAGGGA

HZ4 ACATGGATTGAGTGATTACACTGTGGATTTTATTGGCCATGCACTCGCTCTGCACAGGGA

**************** *********************************************

1145 TGATCGTTACCTAAATGAACCTGCCCTTGATACTGTAAAAAGGATGAAGGTATGTTTTGA

B73 TGATCGTTACCTAAATGAACCTGCCCTTGATACTGTAAAAAGGATGAAGGTATGTTTTGA

HZ4 TGATCGTTACCTAAATGAACCTGCCCTTGATACTGTAAAAAGGATGAAGGTATGTTTTGA

************************************************************

1145 GTGAAGATGTGTTAAACTATTGTTCTTTTTCATATTATAATGTAATTTTATCAAACTACT

B73 GTGAAGATGTGTTAAACTATTGTTCTTTTTCATATTATAATGTAATTTTATCAAACTACT

HZ4 GTGAAGATGTGTTAAACTATTGTTCTTTTTCATATTATAATGTAATTTTATCAAACTACT

**********************************************************

1145 TCCTTGCATATGTAGCCTTTTTTTTATATATTCCAGATTTCTAAGTATTCTTGGATGGG-

B73 TCCTTGCATATGTAGCCTTTTTTTTATATATTCCAGATTTCTAAGTATTCTTGGATCGGG

HZ4 TCCTTGCATATGTAGCCTTTTTTTTATATATTCCAGATTTCTAAGTATTCTTGGATGGG-

******************************************************** **

1145 AACATGATACAATTTTGTTGTCTGTCTTCTCTTAGCTTTACGCTGAGTCTCTTGCACGTT

B73 AACATGATACAATTTTGTTGTCTGTCTTCTCTTAGCTTTACGCTGAGTCTCTTGCACGTT

HZ4 AACATGATACAATTTTGTTGTCTGTCTTCTCTTAGCTTTACGCTGAGTCTCTTGCACGTT

************************************************************

1145 TTCAAGGAGGCTCGCCTTATATTTATCCATTATATGGGTTGGGTGAGCTGCCACAGGTCA

B73 TTCAAGGAGGCTCGCCTTATATTTATCCATTATATGGGTTGGGTGAGCTGCCACAGGTCA

HZ4 TTCAAGGAGGCTCGCCTTATATTTATCCATTATATGGGTTGGGTGAGCTGCCACAGGTCA

*************************************************************

1145 GCTATTTCTCAATTCCTTGCACCAACTCTAGTTGCTTTTATTATTGCACTTTGATGCTTC

B73 GCTATTTCTCAATTCCTTGCACCAACTCTAGTTGCTTTTATTATTGCACTTTGATGCTTC

HZ4 GCTATTTCTCAATTCCTTGCACCAACTCTAGTTGCTTTTATTATTGCACTTTGATGCTTC

************************************************************

1145 TAAGAAGCTTCCATGCTAAGATGATGTCTTATGGTTCAGGCTTTTGCACGTCTAAGTGCT

B73 TAAGAAGCTTCCATGCTAAGATGATGTCTTATGGTTCAGGCTTTTGCACGTCTAAGTGCT

HZ4 TAAGAAGCTTCCATGCTAAGATGATGTCTTATGGTTCAGGCTTTTGCACGTCTAAGTGCT

*************************************************************

1145 GTTTATGGTGGTACATATATGTTAAATAAACCAGAGTGCAAGGTAATGGCTTGAGCTTTT

B73 GTTTATGGTGGTACATATATGTTAAATAAACCAGAGTGCAAGGTAATGGCTTGAGCTTTT

HZ4 GTTTATGGTGGTACATATATGTTAAATAAACCAGAGTGCAAGGTAATGGCTTGAGCTTTT

************************************************************

1145 TTTACTCCTAATTATCATAGTCTGCGAAAACAGCTTATCAAATATGATCTTTTTGCTGAT

B73 TTTACTCCTAATTATCATAGTCTGCGAAAACAGCTTATCAAATATGATCTTTTTGCTGAT

HZ4 TTTACTCCTAATTATCATAGTCTGCGAAAACAGCTTATCAAATATGATCTTTTTGCTGAT

***********************************************************

1145 TTCACTATATAATATGTTTGTAGGTTGAATTTGATATCGAAGGGAAAGTGTGTGGTGTTA

B73 TTCACTATATAATATGTTTGTAGGTTGAATTTGATATCGAAGGGAAAGTGTGTGGTGTTA

HZ4 TTCACTATATAATATGTTTGTAGGTTGAATTTGATATCGAAGGGAAAGTGTGTGGTGTTA

************************************************************

1145 CTTCAGAAGGTGAAACGGCGAAATGCAAAAAGGTTGTCTGTGATCCTTCTTACTTGCCTA

B73 CTTCAGAAGGTGAAACGGCGAAATGCAAAAAGGTTGTCTGTGATCCTTCTTACTTGCCTA

HZ4 CTTCAGAAGGTGAAACGGCGAAATGCAAAAAGGTTGTCTGTGATCCTTCTTACTTGCCTA

*************************************************************

1145 GCAAGGTAAATGCCACTGCCCGTATAAAATTGAACTACTCTGTTTATTATAGGAATTCAC

B73 GCAAGGTAAATGCCACTGCCCGTATAAAATTGAACTACTCTGTTTATTATAGGAATTCAC

HZ4 GCAAGGTAAATGCCACTGCCCGTATAAAATTGAACTACTCTGTTTATTATAGGAATTCAC

************************************************************

1145 TGAAACCTTGATATATTTCAACCAAATCTACTGGAATTTAAACTTCCCTGCTACAATACC

B73 TGAAACCTTGATATATTTCAACCAAATCTACTGGAATTTAAACTTCCCTGCTACAATACC

HZ4 TGAAACCTTGATATATTTCAACCAAATCTACTGGAATTTAAACTTCCCTGCTACAATACC

************************************************************

1145 TCTGTTGACTAATTTCCTATATGGTGCTATCTTGATTTGGGGTTCACTTTGTGGTTGTCA

B73 TCTGTTGACTAATTTCCTATATGGTGCTATCTTGATTTGGGGTTCACTTTGTGGTTGTCA

HZ4 TCTGTTGACTAATTTCCTATATGGTGCTATCTTGATTTGGGGTTCACTTTGTGGTTGTCA

*********************************************************

1145 ATAGGTAAGGAAGATTGGAAAAGTTGCACGTGCAATCGCTATTATGAGCCACCCAATTCC

B73 ATAGGTAAGGAAGATTGGAAAAGTTGCACGTGCAATCGCTATTATGAGCCACCCAATTCC

HZ4 ATAGGTAAGGAAGATTGGAAAAGTTGCACGTGCAATCGCTATTATGAGCCACCCAATTCC

***********************************************************

1145 AAACACAAATGAGTCCCACTCGATTCAGATTATTTTGCCGCAGAAGCAACTTGGGCGCAA

B73 AAACACAAATGAGTCCCACTCGATTCAGATTATTTTGCCGCAGAAGCAACTTGGGCGCAA

HZ4 AAACACAAATGAGTCCCACTCGATTCAGATTATTTTGCCGCAGAAGCAACTTGGGCGCAA

************************************************************

1145 GTCAGACATGTGGGTCTCTTACTCATCTATACTATCTATTAAGAGGATAGTGTAGACCAT

B73 GTCAGACATGTGGGTCTCTTACTCAT-----------------------------------------------------------------

HZ4 GTCAGACATGTGGGTCTCTTACTCAT-----------------------------------------------------------------

**************************

1145 ATACATGCCCCCGCCTCCCCCTCCGCTCACGTCACATCCTCACTCGCCTGTCTGCCACTC

B73 ----------------------------------------------------------------------------------------------------------------------------

HZ4 ----------------------------------------------------------------------------------------------------------------------------

1145 GCCTGCCTCGGTGTCCTCCGCATCGTACTCCATGTCGAATCCTAAGGAACGAAGCCCGAT

B73 ---------------------------------------------------------------------------------------------------------------------------

HZ4 ---------------------------------------------------------------------------------------------------------------------------

1145 CCGCTTATGGGATATCGTGGCCCTGCAATCAGTGACCGTCCAACAGATGCGCGGACAGCT

B73 ----------------------------------------------------------------------------------------------------------------------------

HZ4 ----------------------------------------------------------------------------------------------------------------------------

1145 GTCGGATCAGAAACCCTAGCCCGGGAGGCTCTCGCCTATTATTAGAATCCACCTCGCACC

B73 --------------------------------------------------------------------------------------------------------------------------

HZ4 --------------------------------------------------------------------------------------------------------------------------

1145 CCGCGAGCGGTGACTTCCCTAGGGTTTAGTCGCGTCGCCGCTGCCGCCGCCAAAACAGCA

B73 ------------------------------------------------------------------------------------------------------------------------------

HZ4 ------------------------------------------------------------------------------------------------------------------------------

1145 CATCCCTCTCTCGACCTCAGCCATGGCGGAGGTGGAGCAGCCACCGGACGCGGTGAAGCA

B73 ------------------------------------------------------------------------------------------------------------------------------

HZ4 ------------------------------------------------------------------------------------------------------------------------------

1145 CTTCAACCGCTGGACCTTCGACGATGTCCAGGTACGCCAATGGCTCTACACATCTCTCAG

B73 ---------------------------------------------------------------------------------------------------------------------------

HZ4 ---------------------------------------------------------------------------------------------------------------------------

1145 CCAGCTCAGCCCGGGGAATTCGAGGGGGAAATCGACAGGGATCAGGTGGCGATCTGGGCT

B73 ------------------------------------------------------------------------------------------------------------------------------

HZ4 ------------------------------------------------------------------------------------------------------------------------------

1145 CGGGCGGGCGTATACCTTGCTGGTGGAAGTGAGGTCTAAATCGACAGGGACGACGCCCGC

B73 ------------------------------------------------------------------------------------------------------------------------------

HZ4 ------------------------------------------------------------------------------------------------------------------------------

1145 GCGAGGAGAACCCCGGAGGAGCACGACCATGGGGAGGAGGCGATCGGGGCCCTAGGCGCC

B73 ---------------------------------------------------------------------------------------------------------------------------------

HZ4 ---------------------------------------------------------------------------------------------------------------------------------

1145 GGCAAGGAGATCCGGGGGTGCCGAATTTGGGGCATCAGCGGCCGCGCGTGGGTGCTGCTG

B73 --------------------------------------------------------------------------------------------------------------------------------

HZ4 --------------------------------------------------------------------------------------------------------------------------------

1145 CGGCGGGGCTCCCTCGCGTGGCGCCTCTGTGGCCTCCGCCGTATGCGTCGACGAAGGTTG

B73 -------------------------------------------------------------------------------------------------------------------------------

HZ4 ------------------------------------------------------------------------------------------------------------------------------

1145 CCTGGACGGGGGCGGTGTTTTCCTCTCTCACTCTCTCTCTCTCCACCTCGCTCTTGTCTG

B73 ----------------------------------------------------------------------------------------------------------------------------

HZ4 ---------------------------------------------------------------------------------------------------------------------------

1145 CCTTGGCTCGCACGTGCTTGTGGGACGAGTTCGTCGAGAAGGAGAGGAAGAGAGAGGAGC

B73 -------------------------------------------------------------------------------------------------------------------------------

HZ4 -------------------------------------------------------------------------------------------------------------------------------

1145 GGTTTCTCTCTCCTATTCTCAGCCCGTCAGCCGCCGCGGCGGACGTATCGGCCGCTATCA

B73 ----------------------------------------------------------------------------------------------------------------------------

HZ4 ---------------------------------------------------------------------------------------------------------------------------

1145 AACCCGGTTTTTGGTGGGGACGTACGAGTCCGCGACCCACCGAAGCCGCGGTAAAATGGA

B73 ------------------------------------------------------------------------------------------------------------------------------

HZ4 -----------------------------------------------------------------------------------------------------------------------------

1145 AGCATTTCCCGGATTCTAAGGATTGTTAAGGCCAGAGTCAATGTGTGCCAGAGTCAATGC

B73 --------------------------------------------------------------------------------------------------------------------------

HZ4 -------------------------------------------------------------------------------------------------------------------------

1145 GTGGTGCCGCACGTCCAAGGAAGCGTTTTTTCCTTTCTGGCACTGGCAGCAAATCAGCGT

B73 ----------------------------------------------------------------------------------------------------------------------------

HZ4 ---------------------------------------------------------------------------------------------------------------------------

1145 TCACCGATCTTGATCTTCCATTTGTTAGCAGTAGATACTATACTAGCAGCAATTCTGGTG

B73 ----------------------------------------------------------------------------------------------------------------------

HZ4 ---------------------------------------------------------------------------------------------------------------------

1145 CCGAGACTATCGGGGGATTCGGACGGCACTCCTCTAAGGCAAACTAGATCGGCAGATCGG

B73 ----------------------------------------------------------------------------------------------------------------------------

HZ4 ----------------------------------------------------------------------------------------------------------------------------

1145 TAAAATTGCTGTAATCGTCCAATTGTCAATAGGCACATTTGTTCCAAATGAGCTTACTAG

B73 ----------------------------------------------------------------------------------------------------------------------

HZ4 ---------------------------------------------------------------------------------------------------------------------

1145 CTGAATTTTACCTGCTATGTCTGTTTCGTGATGCTTCTACTCGAGACTCTGTAACTACAT

B73 ----------------------------------------------------------------------------------------------------------------------

HZ4 ----------------------------------------------------------------------------------------------------------------------

1145 GAGCGTTCAATAATTTTCCACGATATGGCGAAGTGATTTTATTAGAGCGTGCAAATAACG

B73 -----------------------------------------------------------------------------------------------------------------------

HZ4 ----------------------------------------------------------------------------------------------------------------------

1145 GTTTACACGAACGAACGACGTCCGCACGAAACATCACGTAAATAACGTGCGGACCAGACG

B73 ----------------------------------------------------------------------------------------------------------------------------

HZ4 ----------------------------------------------------------------------------------------------------------------------------

1145 CACTCGGTAAAATATTTGGCACGAGACAAAGATCCAAATTTCGGTTGTGAATCAAATATT

B73 -----------------------------------------------------------------------------------------------------------------------

HZ4 ----------------------------------------------------------------------------------------------------------------------

1145 TGTAATTGGAAAATTAGCGCCATAAAAATAGCCTCGTTTAAAATCTGCCTTCTAAAAAAT

B73 ----------------------------------------------------------------------------------------------------------------------

HZ4 ----------------------------------------------------------------------------------------------------------------------

1145 AAAAATACGAACTCAGGACCTCGAATGATGTTACTCAGATCATCGCAACTCAGACTGCTT

B73 -------------------------------------------------------------------------------------------------------------------------

HZ4 -------------------------------------------------------------------------------------------------------------------------

1145 GCATGTTTTTTTCACACGTCGCATGGTTTACAAATTGAAACTTCATCTTTCTTATAGAAA

B73 ---------------------------------------------------------------------------------------------------------------------

HZ4 ---------------------------------------------------------------------------------------------------------------------

1145 ACACTCCTAATGCATGCACGGTCTCGCCCAAGGAAACCTCATAGTCATGACTCGCCCAAG

B73 ---------------------------------------------------------------------------------------------------------------------------

HZ4 --------------------------------------------------------------------------------------------------------------------------

1145 GAAACCTCATAGTCACGACTCTCTCGATGCCAACCTCGGGCGGGAGACGCAATCACGCTT

B73 ----------------------------------------------------------------------------------------------------------------------------

HZ4 ----------------------------------------------------------------------------------------------------------------------------

1145 TGGCCAAAGCCATCCTTGGGTGAGAGAATGCATTCACGCCTCGCCCGAGGCCGCCCTCAA

B73 -----------------------------------------------------------------------------------------------------------------------------

HZ4 -----------------------------------------------------------------------------------------------------------------------------

1145 GCGGGAGAACGCATTCATGGCTCGCTCGAGGCCACCCTCGGGCGGGAGACATAGTCACGA

B73 ------------------------------------------------------------------------------------------------------------------------------

HZ4 ---------------------------------------------------------------------------------------------------------------------------

1145 CTCACTTGAGGCCATCCCCAGGCAGGAGACGCAGCCATGGCTTGCCTGAGACCATCCTCG

B73 -----------------------------------------------------------------------------------------------------------------------------

HZ4 ----------------------------------------------------------------------------------------------------------------------------

1145 GGCAGGAGACGCTGTCACGTCTCGCTCGTGGCCGACCTTTGGCGGGAGACATAGTCATAG

B73 -----------------------------------------------------------------------------------------------------------------------------

HZ4 ------------------------------------------------------------------------------------------------------------------------------

1145 CTCGCCCGAGGCAACCCTCGGACGGGAGACGCAATCACGCCTCGCCAGAGGCCAACCTCG

B73 -------------------------------------------------------------------------------------------------------------------------------

HZ4 -------------------------------------------------------------------------------------------------------------------------------

1145 GGCGGGAGACGCAATCACGCCTTGTCCGAGGCCACACTTGGGCGAGAGACGTAGTCACAC

B73 ------------------------------------------------------------------------------------------------------------------------------

HZ4 ------------------------------------------------------------------------------------------------------------------------------

1145 TCGCCCGAGGCCATCCTCGCGCGGGAAACGCAGTCGCGACACGTTTGAGGCCACCCTCGG

B73 -------------------------------------------------------------------------------------------------------------------------------

HZ4 -------------------------------------------------------------------------------------------------------------------------------

1145 GCGGGAGACGCACTCACGCTCGCCCGAGGCCAACCTTGGGCGGGAGATGCAGTCACGGCT

B73 --------------------------------------------------------------------------------------------------------------------------------

HZ4 --------------------------------------------------------------------------------------------------------------------------------

1145 CGTTCGTGACCTCCCTCGGGTGGGAGACGCAAGTTCTTATGACTGATATTTATAAATGGC

B73 -------------------------------------------------------------------------------------------------------------------------

HZ4 -------------------------------------------------------------------------------------------------------------------------

1145 ACTTCTTTGCAAACTTAATGACTGATATTTTGCAGATTCTTATGCCGTGGGAATTCAGTA

B73 ----------------------------------------------------------------------------------------------------------------------

HZ4 ----------------------------------------------------------------------------------------------------------------------

1145 GTTTAAATGAGGTAACCAGTCTACCACGCCGCTTCAAGAACATAGATATGCACGGGTAAT

B73 ------------------------------------------------------------------------------------------------------------------------

HZ4 ------------------------------------------------------------------------------------------------------------------------

1145 AAACTTTTAACGAATAGTTTGTGTTCCGTTGCAACGCACGGGCACCATACTAGTATTTCA

B73 -------------------------------------------------------------------------------------------------------------ATTTCA

HZ4 -------------------------------------------------------------------------------------------------------------ATTTCA

******

1145 AATAGTTTTCATTTGATGAGTGCCTTAATTCAGTTTGTACTGCTTGGTTGTGTGTTTGTA

B73 AATAGTTTTCATTTGATGAGTGCCTTAATTCAGTTTGTACTGCTTGGTTGTGTGTTTGTA

HZ4 AATAGTTTTCATTTGATGAGTGCCTTAATTCAGTTTGTACTGCTTGGTTGTGTGTTTGTA

*********************************************************

1145 ACATGTATTCTTAAAATGTTTCTTAGGTATGTTTTCTGTTGCTCATATACACATAATGTT

B73 ACATGTATTCTTAAAATGTTTCTTAGGTATGTTTTCTGTTGCTCATATACACATAATGTT

HZ4 ACATGTATTCTTAAAATGTTTCTTAGGTATGTTTTCTGTTGCTCATATACACATAATGTT

********************************************************

1145 GCGCCAAAAGGGAAGTTCATTGCATTTGTGTCTGCGGAAGCTGAGACCGATAATCCACAG

B73 GCGCCAAAAGGGAAGTTCATTGCATTTGTGTCTGCGGAAGCTGAGACCGATAATCCACAG

HZ4 GCGCCAAAAGGGAAGTTCATTGCATTTGTGTCTGCGGAAGCTGAGACCGATAATCCACAG

************************************************************

1145 TCCGAACTAAAGCCTGGAATTGATCTACTTGGTCAAGTAGATGAACTGTTTTTTGATATG

B73 TCCGAACTAAAGCCTGGAATTGATCTACTTGGTCAAGTAGATGAACTGTTTTTTGATATG

HZ4 TCCGAACTAAAGCCTGGAATTGATCTACTTGGTCAAGTAGATGAACTGTTTTTTGATATG

**********************************************************

1145 TATGACAGATACAAACCTGTCAATGAACCATCTCTTGATAATTGCTTTGTTTCAATGGTA

B73 TATGACAGATACAAACCTGTCAATGAACCATCTCTTGATAATTGCTTTGTTTCAATGGTA

HZ4 TATGACAGATACAAACCTGTCAATGAACCATCTCTTGATAATTGCTTTGTTTCAATGGTA

*********************************************************

1145 AGTTTAGGATTATGTAATAACAAAGTGATTACAAGTCCCAGATATCTTAGATGTTTATGG

B73 AGTTTAGGATTATGTAATAACAAAGTGATTACAAGTCCCAGATATCTTAGATGTTTATGG

HZ4 AGTTTAGGATTATGTAATAACAAAGTGATTACAAGTCCCAGATATCTTAGATGTTTATGG

*********************************************************

1145 AAGTTATCTTCTTTGCAGAGTTATGATGCTACTACACACTTTGAGACAACTGTGACAGAT

B73 AAGTTATCTTCTTTGCAGAGTTATGATGCTACTACACACTTTGAGACAACTGTGACAGAT

HZ4 AAGTTATCTTCTTTGCAGAGTTATGATGCTACTACACACTTTGAGACAACTGTGACAGAT

**********************************************************

1145 GTTCTCAGTATGTACACAGCAATTACTGGAAAGGTAAGGTTCATGTCCCTTTTTTTTGTG

B73 GTTCTCAGTATGTACACAGCAATTACTGGAAAGGTAAGGTTCATGTCCCTTTTTTTTGTG

HZ4 GTTCTCAGTGTGTACACAGCAATTACTGGAAAGGTAAGGTTCATGTCCCTTTTTTTTGTG

********* *************************************************

1145 GTCATCAAAGCAGATGTATCAAATACTAATATCTACAATGCTGTATTGGGCCTAGAACAA

B73 GTCATCAAAGCAGATGTATCAAATACTAATATCTACAATGCTGTATTGGGCCTAGAACAA

HZ4 GTCATCAAAGCAGATGTATCAAATACTAATATCTACAATGCTGTATTGGGCCTAGAACAA

*********************************************************

1145 TAACAACAAGCAAGTTGGATAGGCTAGAGTTCAATTCAAATCCAACATGAACCACAAGTT

B73 TAACAACAAGCAAGTTGGATAGGCTAGAGTTCAATTCAAATCCAACATGAACCACAAGTT

HZ4 TAACAACAAGCAAGTTGGATAGGCTAGAGTTCAATTCAAATCCAACATGAACCACAAGTT

**********************************************************

1145 AAAGTTAAGGCATATGGATAGCTGTTTTTCATGCATTCCTATTCATGGCTAAATCTTTGG

B73 AAAGTTAAGGCATATGGATAGCTGTTTTTCATGCATTCCTATTCATGGCTAAATCTTTGG

HZ4 AAAGTTAAGGCATATGGATAGCTGTTTTTCATGCATTCCTATTCATGGCTAAATCTTTGG

*********************************************************

1145 GTATATACTATCTTTTCAAGTCTCCTAAATAAGGGGCTGTCGACCCATCTCTTCCCCATT

B73 GTATATACTATCTTTTCAAGTCTCCTAAATAAGGGGCTGTCGACCCATCTCTTCCCCATT

HZ4 GTATATACTATCTTTTCAAGTCTCCTAAATAAGGGGCTGTCGACCCATCTCTTCCCCATT

*********************************************************

1145 CAACACTGTACCTGGTTCCGATATGACACATCCATTAGAGGGTTACGCTCTAACGGACTA

B73 CAACACTGTACCTGGTTCCGATATGACACATCCATTAGAGGGTTACGCTCTAACGGACTA

HZ4 CAACACTGTACCTGGTTCCGATATGACACATCCATTAGAGGGTTACGCTCTAACGGACTA

***********************************************************

1145 CTTTGGGTTTCATCCTTATTAGAATGGTTAGGTTTAACATCGGATTGGGACTTGTTATGT

B73 CTTTGGGTTTCATCCTTATTAGAATGGTTAGGTTTAACATCGGATTGGGACTTGTTATGT

HZ4 CTTTGGGTTTCATCCTTATTAGAATGGTTAGGTTTAACATCGGATTGGGACTTGTTATGT

*********************************************************

1145 TGAGAAAACATAGGTGGATTTCAATACTGTATTGGGCCTGAGAGATCAAAACCTACTACT

B73 TGAGAAAACATAGGTGGATTTCAATACTGTATTGGGCCTGAGAGATCAAAACCTACTACT

HZ4 TGAGAAAACATAGGTGGATTTCAATACTGTATTGGGCCTGAGAGATCAAAACCTACTACT

**********************************************************

1145 CTACTAGTGAAATTTGCTAGCTTTACAGGGCATGGTCAATTGTTTGTTAACCTTCTGTCA

B73 CTACTAGTGAAATTTGCTAGCTTTACAGGGCATGGTCAATTGTTTGTTAACCTTCTGTCA

HZ4 CTACTAGTGAAATTTGCTAGCTTTACAGGGCATGGTCAATTGTTTGTTAACCTTCTGTCA

**********************************************************

1145 GCAATAGATGATTTGCCTCAAACAAAATTAGTCCCTTACTAGACATCTCTCTTTATTATT

B73 GCAATAGATGATTTGCCTCAAACAAAATTAGTCCCTTACTAGACATCTCTCTTTATTATT

HZ4 GCAATAGATGATTTGCCTCAAACAAAATTAGTCCCTTACTAGACATCTCTCTTTATTATT

*********************************************************

1145 GATACTCTGATGACATTACTATTTTATGTTTACTGACCCAAAGATGGATCGCACATCTTT

B73 GATACTGTGATGACATTACTATTTTATGTTTACTGACCCAAAGATGGATCGCACATCTTT

HZ4 GATACTGTGATGACATTACTATTTTATGTTTACTGACCCAAAGATGGATCGCACATCTTT

****** **************************************************

1145 AAATCTCTCCATGTCTGTGTCGCAGACCGTTGATCTCAGTGTGGACCTGAGCGCTGCCAG

B73 AAATCTCTCCATGTCTGTGTCGCAGACCGTTGATCTCAGTGTGGACCTGAGCGCTGCCAG

HZ4 AAATCTCTCCATGTCTGTGTCGCAGACCGTTGATCTCAGTGTGGACCTGAGCGCTGCCAG

************************************************************

*ZmGDIα* stop codon

1145 CGCAGCTGAAGAATACTAG

B73 CGCAGCTGAAGAATACTAG

HZ4 CGCAGCTGAAGAATACTAG

*******************
