# Supplementary material for: A helitron-induced RabGDIα variant causes quantitative recessive resistance to maize rough dwarf disease
Source: Nat Commun. 2020 Jan 24;11:495. doi: 10.1038/s41467-020-14372-3 (PMC6981192; doi:10.1038/s41467-020-14372-3)
Supplement: Supplementary file 7 — Supplementary Data 3 [file 41467_2020_14372_MOESM7_ESM.docx]

**Supplementary Data 3.** Clustal alignment of cDNAs of *ZmGDIα*and *ZmGDIα-hel.*

ZmGDIα-S ATGGACGAGGAGTACGACGTGATCGTTCTGGGCACGGGGCTCAAGGAGTGCATCCTCAGC

ZmGDIα-L ATGGACGAGGAGTACGACGTGATCGTTCTGGGCACGGGGCTCAAGGAGTGCATCCTCAGC

ZmGDIα-hel-S ATGGACGAGGAGTACGACGTGATCGTTCTGGGCACGGGGCTCAAGGAGTGCATCCTCAGC

ZmGDIα-hel-L ATGGACGAGGAGTACGACGTGATCGTTCTGGGCACGGGGCTCAAGGAGTGCATCCTCAGC

***********************************************************

ZmGDIα-S GGTCTCCTCTCTGTCGACGGCCTCAAGGTTCTACACATGGATAGAAATGATTACTACGGA

ZmGDIα-L GGTCTCCTCTCTGTCGACGGCCTCAAGGTTCTACACATGGATAGAAATGATTACTACGGA

ZmGDIα-hel-S GGTCTCCTCTCTGTCGACGGCCTCAAGGTTCTACACATGGATAGAAATGATTACTACGGA

ZmGDIα-hel-L GGTCTCCTCTCTGTCGACGGCCTCAAGGTTCTACACATGGATAGAAATGATTACTACGGA

************************************************************

ZmGDIα-S GGAGATTCCACCTCCCTAAACCTGAACCAGCTCTGGAAGAGGTTTAGGGGGGAAGACAAG

ZmGDIα-L GGAGATTCCACCTCCCTAAACCTGAACCAGCTCTGGAAGAGGTTTAGGGGGGAAGACAAG

ZmGDIα-hel-S GGAGATTCCACCTCCCTAAACCTGAACCAGCTCTGGAAGAGGTTTAGGGGGGAAGACAAG

ZmGDIα-hel-L GGAGATTCCACCTCCCTAAACCTGAACCAGCTCTGGAAGAGGTTTAGGGGGGAAGACAAG

************************************************************

Extra exon 4

ZmGDIα-S CCACCGGCACATCTAGGTGCAAGCAGAGATTACAATGTAGACATGGTTCCAAAG------

ZmGDIα-L CCACCGGCACATCTAGGTGCAAGCAGAGATTACAATGTAGACATGGTTCCAAAGTATCAT

ZmGDIα-hel-S CCACCGGCACATCTAGGTGCAAGCAGAGATTACAATGTAGACATGGTTCCAAAG------

ZmGDIα-hel-L CCACCGGCACATCTAGGTGCAAGCAGAGATTACAATGTAGACATGGTTCCAAAGTATCAT

******************************************************

ZmGDIα-S ------------------------------------------------------------

ZmGDIα-L GCAGACTTCGATATATGTCACGATTTGCATGGCCTCATTATTGGAGAGCACGTTTCACAC

ZmGDIα-hel-S ------------------------------------------------------------

ZmGDIα-hel-L GCAGACTTCGATATATGTCACGATTTGCATGGCCTCATTATTGGAGAGCACGTTTCACAC

ZmGDIα-S ------------------------------------------------------------

ZmGDIα-L ATACCGGAAGAAATTCGCTCGTACATCGTTAGTCATCGGACACGTACCACCTTACACCTT

ZmGDIα-hel-S ------------------------------------------------------------

ZmGDIα-hel-L ATACCGGAAGAAATTCGCTCGTACATCGTTAGTCATCGGACACGTACCACCTTACACCTT

ZmGDIα-S --------------TTTATGATGGCAAACGGGACTTTGGTTCGCACTCTCATTCACACT

ZmGDIα-L TCCTTAAACAAAAAGTTTATGATGGCAAACGGGACTTTGGTTCGCACTCTCATTCACACT

ZmGDIα-hel-S ---------------TTTATGATGGCAAACGGGACTTTGGTTCGCACTCTCATTCACACT

ZmGDIα-hel-L TCCTTAAACAAAAAGTTTATGATGGCAAACGGGACTTTGGTTCGCACTCTCATTCACACT

*********************************************

ZmGDIα-S GATGTGACAAAATATTTGTCATTCAAAGCTGTTGATGGAAGCTATGTCTTCAGCAAACGG

ZmGDIα-L GATGTGACAAAATATTTGTCATTCAAAGCTGTTGATGGAAGCTATGTCTTCAGCAAACGG

ZmGDIα-hel-S GATGTGACAAAATATTTGTCATTCAAAGCTGTTGATGGAAGCTATGTCTTCAGCAAACGG

ZmGDIα-hel-L GATGTGACAAAATATTTGTCATTCAAAGCTGTTGATGGAAGCTATGTCTTCAGCAAACGG

************************************************************

ZmGDIα-S AAGATTCACAAGGTTCCTGCCACCGATATGGAGGCTCTAAAATCTCCTTTGATGGGTCTA

ZmGDIα-L AAGATTCACAAGGTTCCTGCCACCGATATGGAGGCTCTAAAATCTCCTTTGATGGGTCTA

ZmGDIα-hel-S AAGATTCACAAGGTTCCTGCCACCGATATGGAGGCTCTAAAATCTCCTTTGATGGGTCTA

ZmGDIα-hel-L AAGATTCACAAGGTTCCTGCCACCGATATGGAGGCTCTAAAATCTCCTTTGATGGGTCTA

************************************************************

ZmGDIα-S TTTGAGAAACGTAGAGCAAGGAACTTTTTTGTTTACGTCCAAAATTACAATGAAGCTGAT

ZmGDIα-L TTTGAGAAACGTAGAGCAAGGAACTTTTTTGTTTACGTCCAAAATTACAATGAAGCTGAT

ZmGDIα-hel-S TTTGAGAAACGTAGAGCAAGGAACTTTTTTGTTTACGTCCAAAATTACAATGAAGCTGAT

ZmGDIα-hel-L TTTGAGAAACGTAGAGCAAGGAACTTTTTTGTTTACGTCCAAAATTACAATGAAGCTGAT

************************************************************

ZmGDIα-S CCAGTGACACATCAGGGGTTGGACCTCACAAGGATTACAACTAGAGAATTGATTTTGAAA

ZmGDIα-L CCAGTGACACATCAGGGGTTGGACCTCACAAGGATTACAACTAGAGAATTGATTTTGAAA

ZmGDIα-hel-S CCAGTGACACATCAGGGGTTGGACCTCACAAGGATTACAACTAGAGAATTGATTTTGAAA

ZmGDIα-hel-L CCAGTGACACATCAGGGGTTGGACCTCACAAGGATTACAACTAGAGAATTGATTTTGAAA

************************************************************

ZmGDIα-S CATGGATTGAGTGATGACACTGTGGATTTTATTGGCCATGCACTCGCTCTGCACAGGGAT

ZmGDIα-L CATGGATTGAGTGATGACACTGTGGATTTTATTGGCCATGCACTCGCTCTGCACAGGGAT

ZmGDIα-hel-S CATGGATTGAGTGATGACACTGTGGATTTTATTGGCCATGCACTCGCTCTGCACAGGGAT

ZmGDIα-hel-L CATGGATTGAGTGATGACACTGTGGATTTTATTGGCCATGCACTCGCTCTGCACAGGGAT

************************************************************

ZmGDIα-S GATCGTTACCTAAATGAACCTGCCCTTGATACTGTAAAAAGGATGAAGCTTTACGCTGAG

ZmGDIα-L GATCGTTACCTAAATGAACCTGCCCTTGATACTGTAAAAAGGATGAAGCTTTACGCTGAG

ZmGDIα-hel-S GATCGTTACCTAAATGAACCTGCCCTTGATACTGTAAAAAGGATGAAGCTTTACGCTGAG

ZmGDIα-hel-L GATCGTTACCTAAATGAACCTGCCCTTGATACTGTAAAAAGGATGAAGCTTTACGCTGAG

************************************************************

ZmGDIα-S TCTCTTGCACGTTTTCAAGGAGGCTCGCCTTATATTTATCCATTATATGGGTTGGGTGAG

ZmGDIα-L TCTCTTGCACGTTTTCAAGGAGGCTCGCCTTATATTTATCCATTATATGGGTTGGGTGAG

ZmGDIα-hel-S TCTCTTGCACGTTTTCAAGGAGGCTCGCCTTATATTTATCCATTATATGGGTTGGGTGAG

ZmGDIα-hel-L TCTCTTGCACGTTTTCAAGGAGGCTCGCCTTATATTTATCCATTATATGGGTTGGGTGAG

************************************************************

ZmGDIα-S CTGCCACAGGCTTTTGCACGTCTAAGTGCTGTTTATGGTGGTACATATATGTTAAATAAA

ZmGDIα-L CTGCCACAGGCTTTTGCACGTCTAAGTGCTGTTTATGGTGGTACATATATGTTAAATAAA

ZmGDIα-hel-S CTGCCACAGGCTTTTGCACGTCTAAGTGCTGTTTATGGTGGTACATATATGTTAAATAAA

ZmGDIα-hel-L CTGCCACAGGCTTTTGCACGTCTAAGTGCTGTTTATGGTGGTACATATATGTTAAATAAA

************************************************************

ZmGDIα-S CCAGAGTGCAAGGTTGAATTTGATATCGAAGGGAAAGTGTGTGGTGTTACTTCAGAAGGT

ZmGDIα-L CCAGAGTGCAAGGTTGAATTTGATATCGAAGGGAAAGTGTGTGGTGTTACTTCAGAAGGT

ZmGDIα-hel-S CCAGAGTGCAAGGTTGAATTTGATATCGAAGGGAAAGTGTGTGGTGTTACTTCAGAAGGT

ZmGDIα-hel-L CCAGAGTGCAAGGTTGAATTTGATATCGAAGGGAAAGTGTGTGGTGTTACTTCAGAAGGT

************************************************************

ZmGDIα-S GAAACGGCGAAATGCAAAAAGGTTGTCTGTGATCCTTCTTACTTGCCTAGCAAG*GTAAGG*

ZmGDIα-L GAAACGGCGAAATGCAAAAAGGTTGTCTGTGATCCTTCTTACTTGCCTAGCAAG*GTAAGG*

ZmGDIα-hel-S GAAACGGCGAAATGCAAAAAGGTTGTCTGTGATCCTTCTTACTTGCCTAGCAAG*ATTCTT*

ZmGDIα-hel-L GAAACGGCGAAATGCAAAAAGGTTGTCTGTGATCCTTCTTACTTGCCTAGCAAG*ATTCTT*

*******************************************************

Exon 10, Italics capital letters

ZmGDIα-S *AAGATTGGAAAAGTTGCACGTGCAATCGCTATTATGAGCCACCCAATTCCAAACACAAAT*

ZmGDIα-L *AAGATTGGAAAAGTTGCACGTGCAATCGCTATTATGAGCCACCCAATTCCAAACACAAAT*

ZmGDIα-hel-S  *ATGCCG*--------*TGGGAATTCAGTAGTTTAAATGAGGTAACCAGT*--*CTACCAC*----

ZmGDIα-hel-L *ATGCCG*--------*TGGGAATTCAGTAGTTTAAATGAGGTAACCAGT*--*CTACCAC*----

****** * * ** *** ** * *******

ZmGDIα-S *GAGTCCCACTCGATTCAGATTATTTTGCCGCAGAAGCAACTTGGGCGCAAGTCAGACAT*G

ZmGDIα-L *GAGTCCCACTCGATTCAGATTATTTTGCCGCAGAAGCAACTTGGGCGCAAGTCAGACAT*G

ZmGDIα-hel-S ----------------------------*GCCGCTTCAAGAACATAG-----ATATGCACGG*G

ZmGDIα-hel-L ----------------------------*GCCGCTTCAAGAACATAG-----ATATGCACGG*G

***** * *** * * * *

ZmGDIα-S TATGTTTTCTGTTGCTCATATACACATAATGTTGCGCCAAAAGGGAAGTTCATTGCATTT

ZmGDIα-L TATGTTTTCTGTTGCTCATATACACATAATGTTGCGCCAAAAGGGAAGTTCATTGCATTT

ZmGDIα-hel-S TATGTTTTCTGTTGCTCATATACACATAATGTTGCGCCAAAAGGGAAGTTCATTGCATTT

ZmGDIα-hel-L TATGTTTTCTGTTGCTCATATACACATAATGTTGCGCCAAAAGGGAAGTTCATTGCATTT

************************************************************

ZmGDIα-S GTGTCTGCGGAAGCTGAGACCGATAATCCACAGTCCGAACTAAAGCCTGGAATTGATCTA

ZmGDIα-L GTGTCTGCGGAAGCTGAGACCGATAATCCACAGTCCGAACTAAAGCCTGGAATTGATCTA

ZmGDIα-hel-S GTGTCTGCGGAAGCTGAGACCGATAATCCACAGTCCGAACTAAAGCCTGGAATTGATCTA

ZmGDIα-hel-L GTGTCTGCGGAAGCTGAGACCGATAATCCACAGTCCGAACTAAAGCCTGGAATTGATCTA

************************************************************

ZmGDIα-S CTTGGTCAAGTAGATGAACTGTTTTTTGATATGTATGACAGATACAAACCTGTCAATGAA

ZmGDIα-L CTTGGTCAAGTAGATGAACTGTTTTTTGATATGTATGACAGATACAAACCTGTCAATGAA

ZmGDIα-hel-S CTTGGTCAAGTAGATGAACTGTTTTTTGATATGTATGACAGATACAAACCTGTCAATGAA

ZmGDIα-hel-L CTTGGTCAAGTAGATGAACTGTTTTTTGATATGTATGACAGATACAAACCTGTCAATGAA

************************************************************

ZmGDIα-S CCATCTCTTGATAATTGCTTTGTTTCAATGAGTTATGATGCTACTACACACTTTGAGACA

ZmGDIα-L CCATCTCTTGATAATTGCTTTGTTTCAATGAGTTATGATGCTACTACACACTTTGAGACA

ZmGDIα-hel-S CCATCTCTTGATAATTGCTTTGTTTCAATGAGTTATGATGCTACTACACACTTTGAGACA

ZmGDIα-hel-L CCATCTCTTGATAATTGCTTTGTTTCAATGAGTTATGATGCTACTACACACTTTGAGACA

************************************************************

ZmGDIα-S ACTGTGACAGATGTTCTCAGTATGTACACAGCAATTACTGGAAAGACCGTTGATCTCAGT

ZmGDIα-L ACTGTGACAGATGTTCTCAGTATGTACACAGCAATTACTGGAAAGACCGTTGATCTCAGT

ZmGDIα-hel-S ACTGTGACAGATGTTCTCAGTATGTACACAGCAATTACTGGAAAGACCGTTGATCTCAGT

ZmGDIα-hel-L ACTGTGACAGATGTTCTCAGTATGTACACAGCAATTACTGGAAAGACCGTTGATCTCAGT

************************************************************

ZmGDIα-S GTGGACCTGAGCGCTGCCAGCGCAGCTGAAGAATACTAG

ZmGDIα-L GTGGACCTGAGCGCTGCCAGCGCAGCTGAAGAATACTAG

ZmGDIα-hel-S GTGGACCTGAGCGCTGCCAGCGCAGCTGAAGAATACTAG

ZmGDIα-hel-L GTGGACCTGAGCGCTGCCAGCGCAGCTGAAGAATACTAG

***************************************
