# Supplementary material for: A helitron-induced RabGDIα variant causes quantitative recessive resistance to maize rough dwarf disease
Source: Nat Commun. 2020 Jan 24;11:495. doi: 10.1038/s41467-020-14372-3 (PMC6981192; doi:10.1038/s41467-020-14372-3)
Supplement: Supplementary file 13 — Source Data [file 41467_2020_14372_MOESM13_ESM.zip › Figure 5a-d.pptx]

## Slide 1
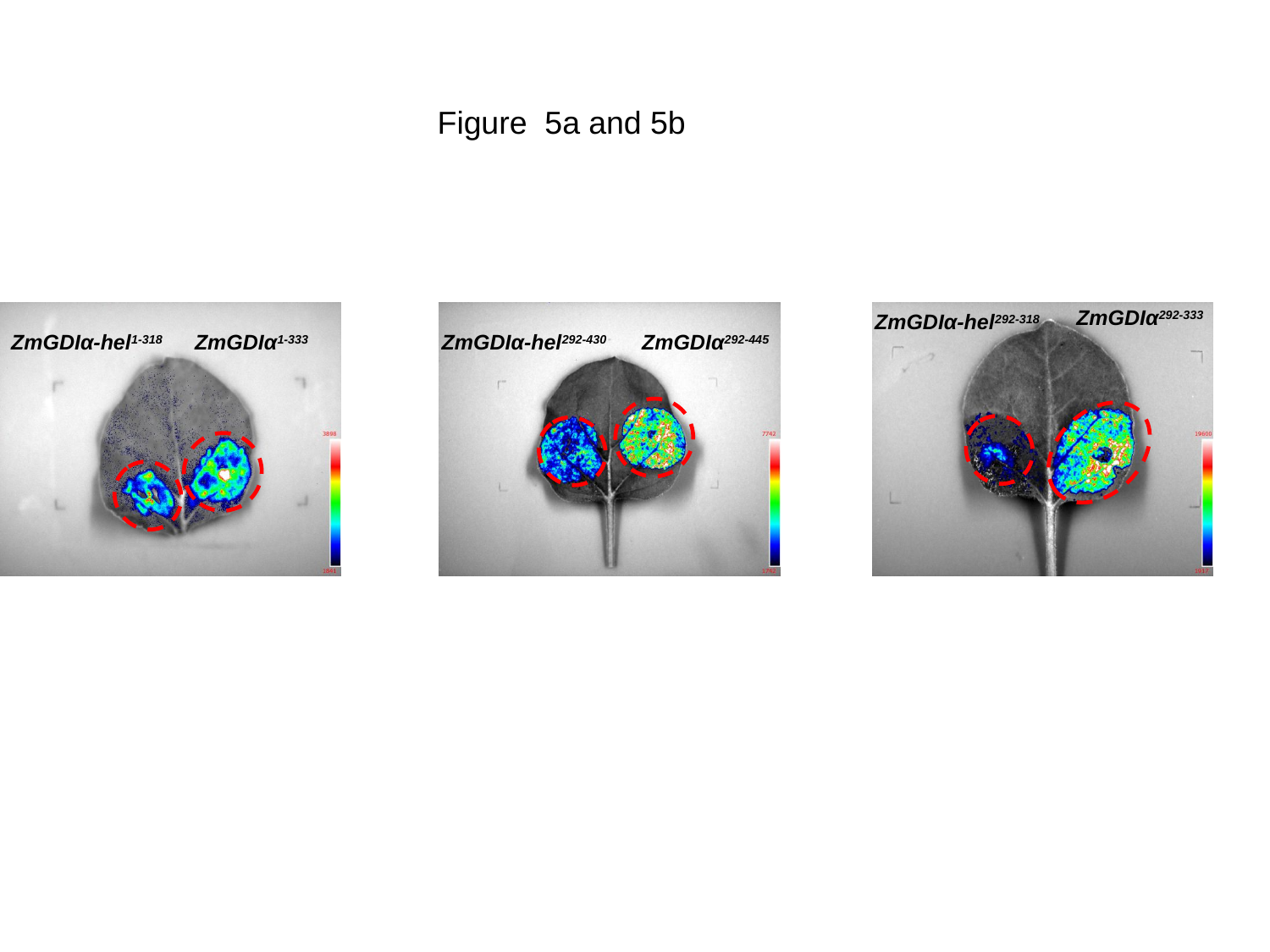

Figure 5a and 5b
ZmGDIα292-333
ZmGDIα-hel292-318
ZmGDIα-hel1-318
ZmGDIα1-333
ZmGDIα292-445
ZmGDIα-hel292-430

## Slide 2
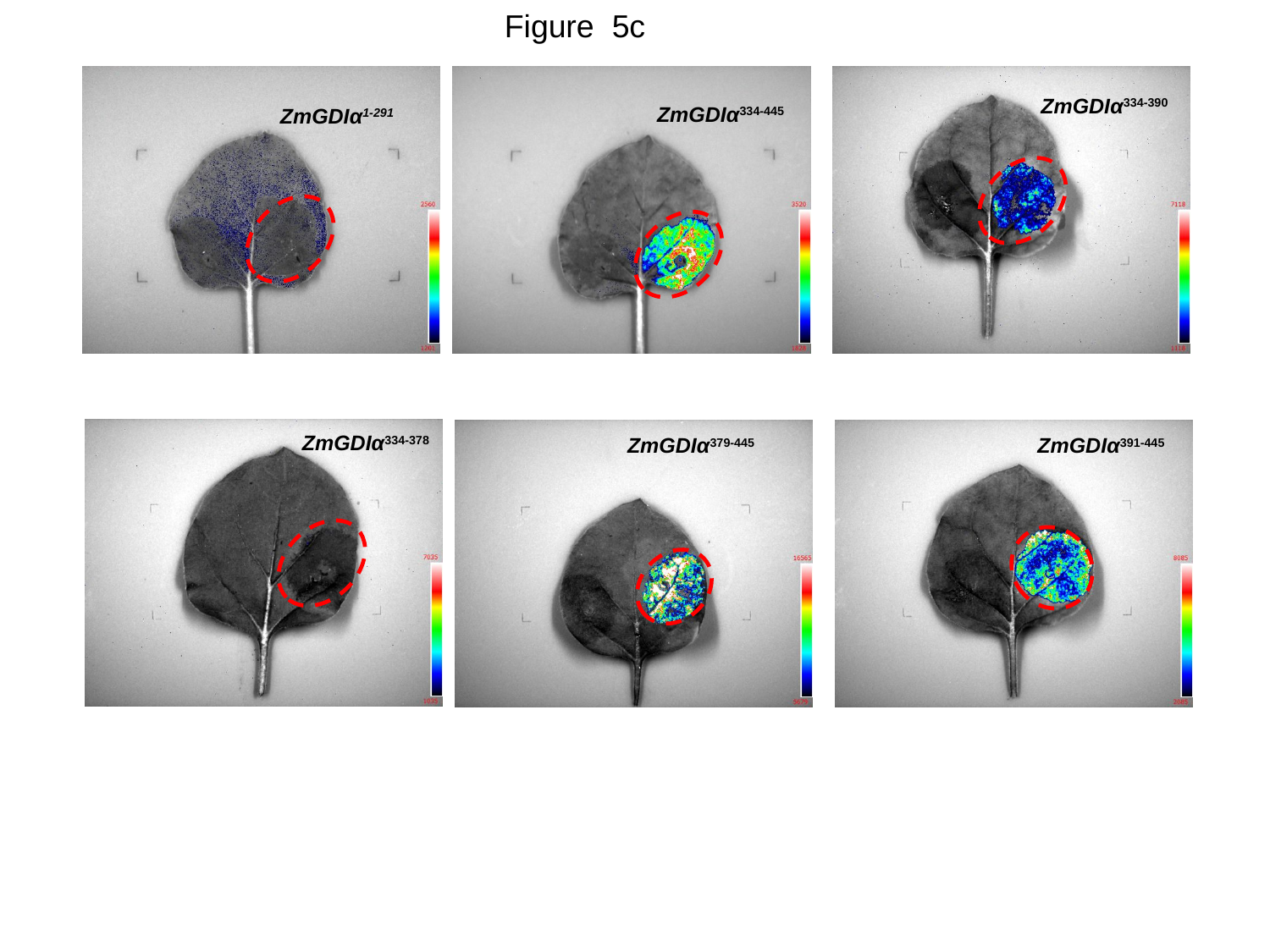

Figure 5c
ZmGDIα1-291
ZmGDIα334-445
ZmGDIα334-390
ZmGDIα334-378
ZmGDIα379-445
ZmGDIα391-445

## Slide 3
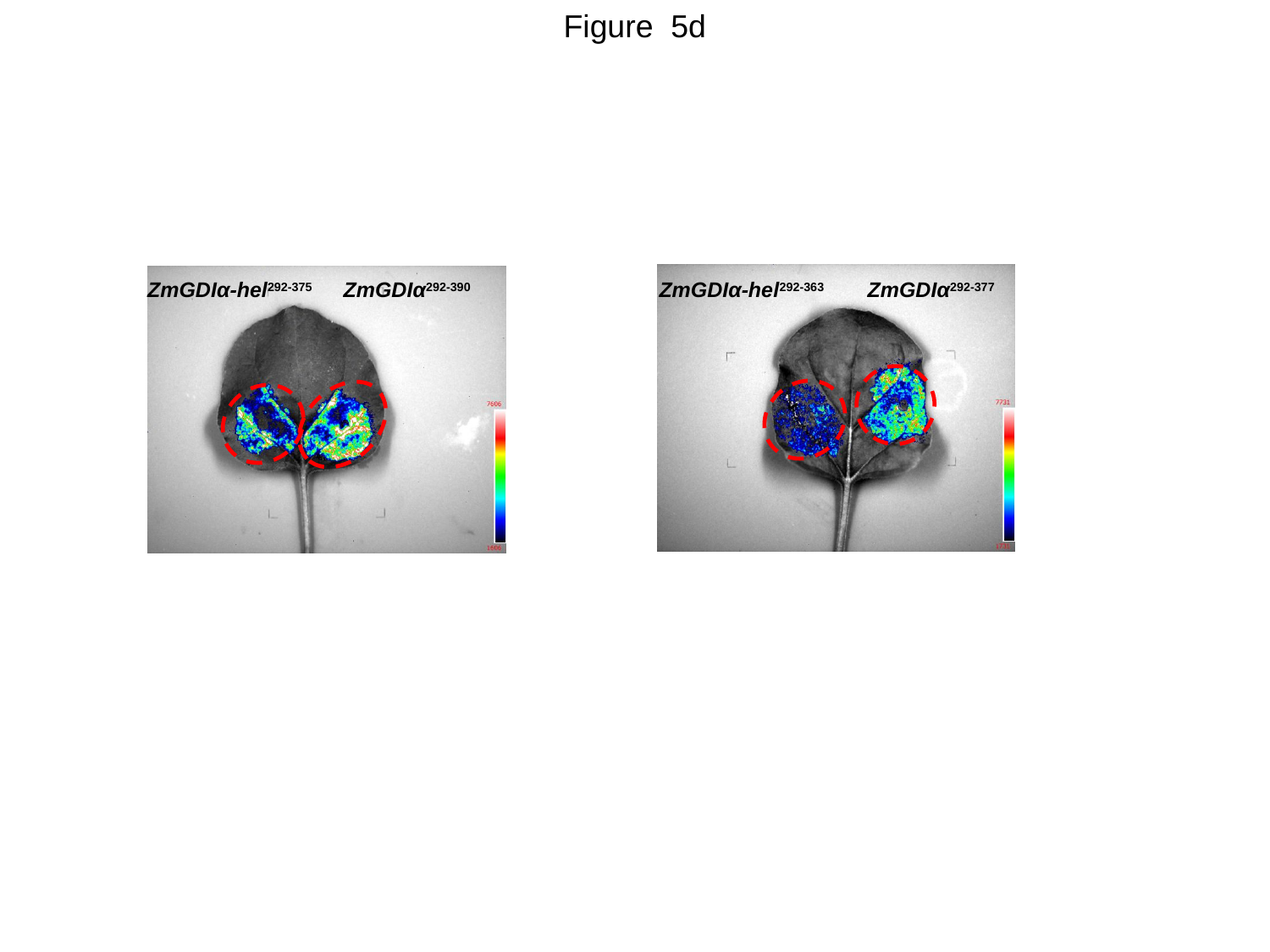

Figure 5d
ZmGDIα-hel292-375
ZmGDIα292-390
ZmGDIα-hel292-363
ZmGDIα292-377
