# Supplementary material for: A helitron-induced RabGDIα variant causes quantitative recessive resistance to maize rough dwarf disease
Source: Nat Commun. 2020 Jan 24;11:495. doi: 10.1038/s41467-020-14372-3 (PMC6981192; doi:10.1038/s41467-020-14372-3)
Supplement: Supplementary file 13 — Source Data [file 41467_2020_14372_MOESM13_ESM.zip › Supplementary Figure 7f.pptx]

## Slide 1
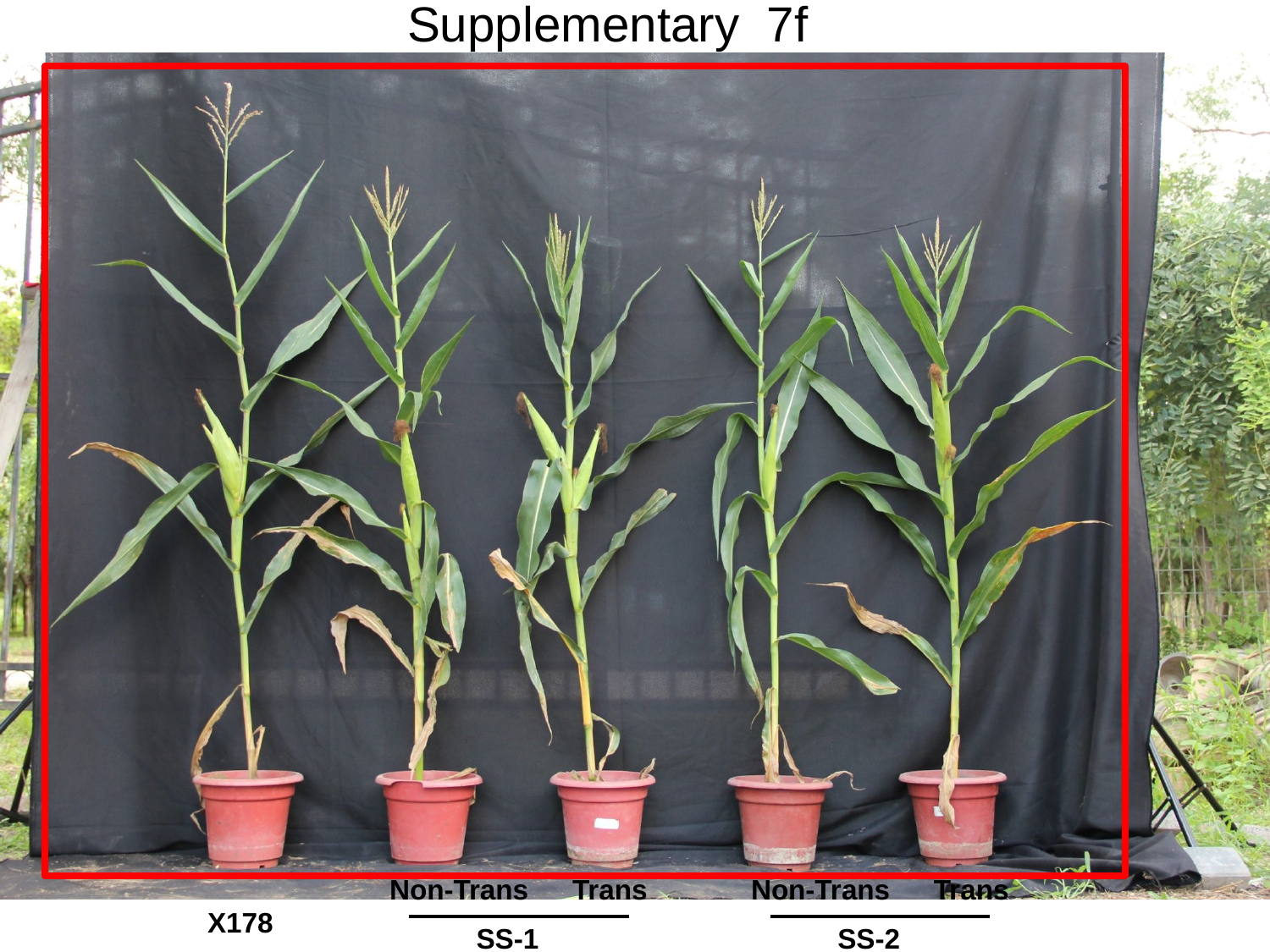

Supplementary 7f
 Non-Trans
Trans
SS-1
 Non-Trans
Trans
SS-2
X178

## Slide 2
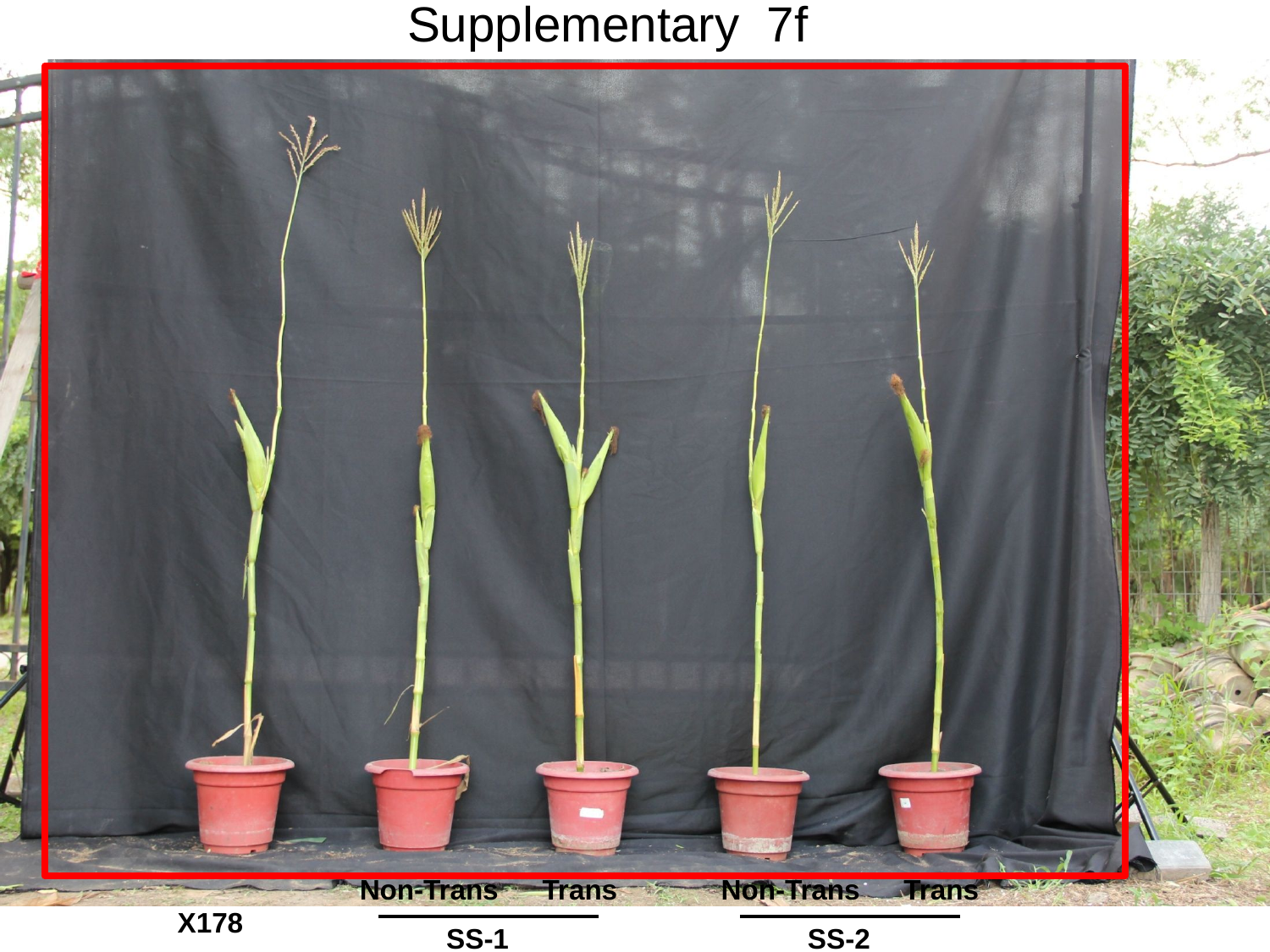

Supplementary 7f
 Non-Trans
Trans
SS-1
 Non-Trans
Trans
SS-2
X178
