# Supplementary material for: A helitron-induced RabGDIα variant causes quantitative recessive resistance to maize rough dwarf disease
Source: Nat Commun. 2020 Jan 24;11:495. doi: 10.1038/s41467-020-14372-3 (PMC6981192; doi:10.1038/s41467-020-14372-3)
Supplement: Supplementary file 13 — Source Data [file 41467_2020_14372_MOESM13_ESM.zip › supplementary Figure 8e.pptx]

## Slide 1
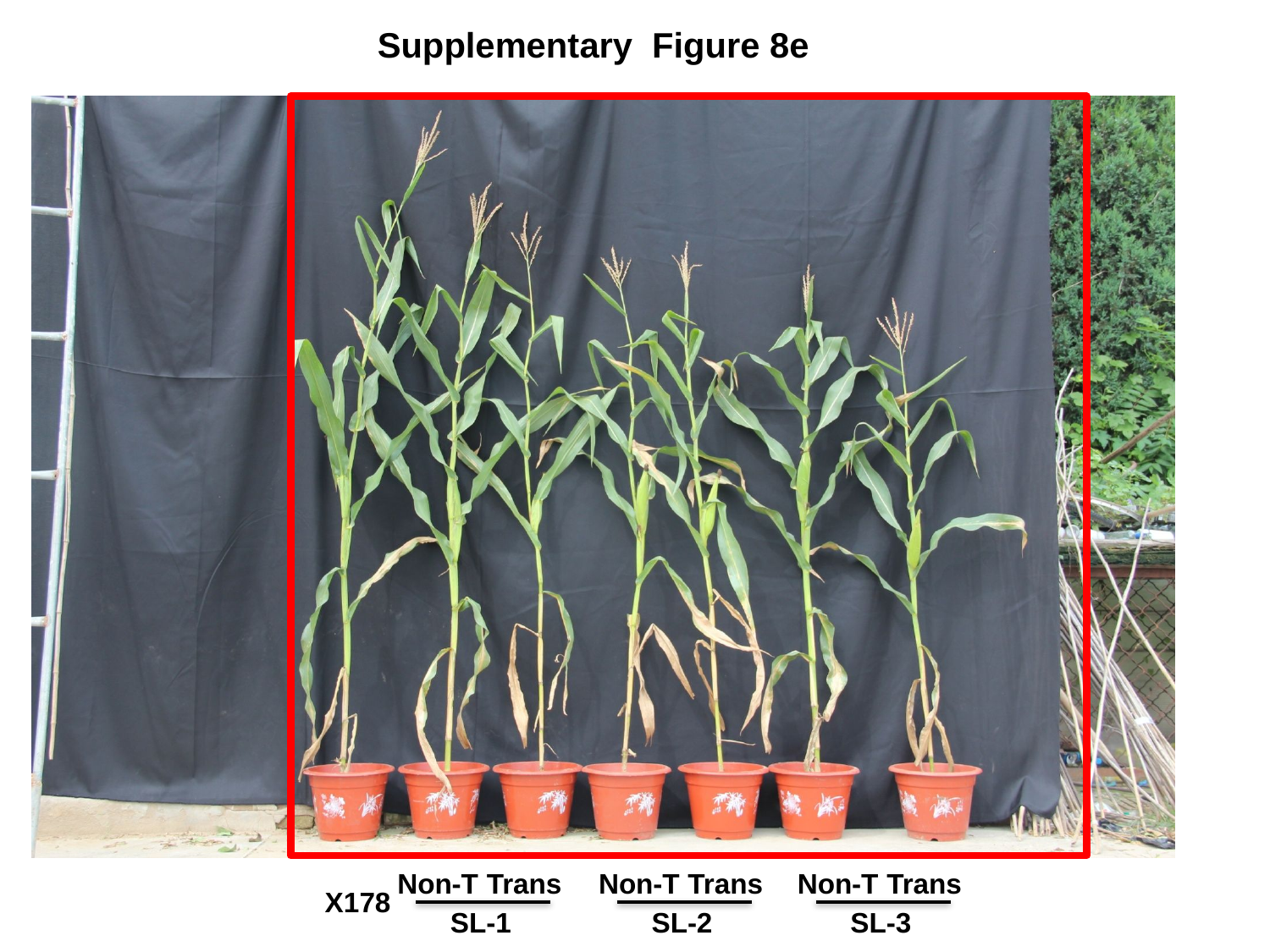

Supplementary Figure 8e
Non-T
Trans
SL-1
Non-T
Trans
SL-2
Non-T
Trans
SL-3
X178

## Slide 2
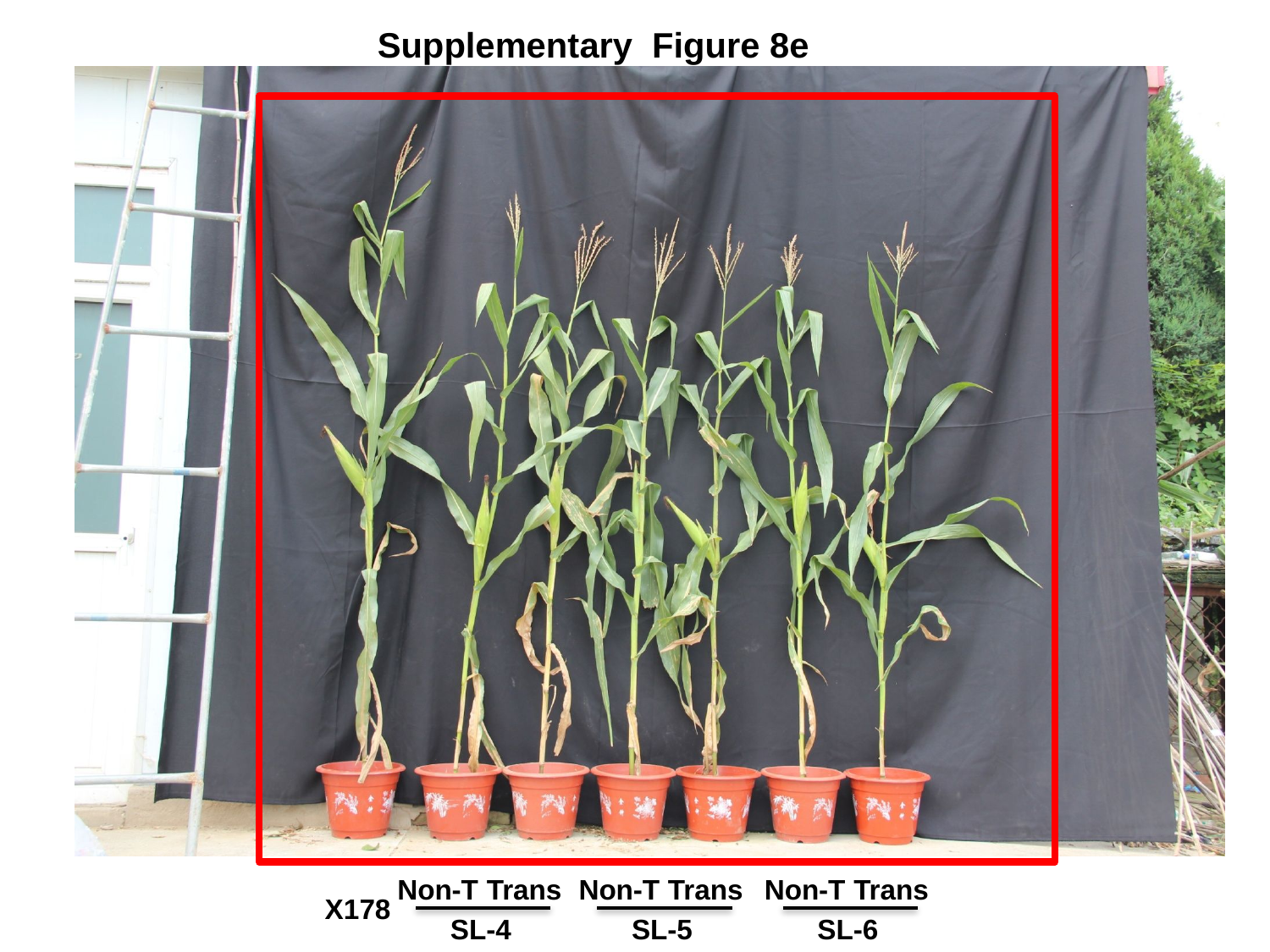

Supplementary Figure 8e
Non-T
Trans
SL-4
Non-T
Trans
SL-5
Non-T
Trans
SL-6
X178

## Slide 3
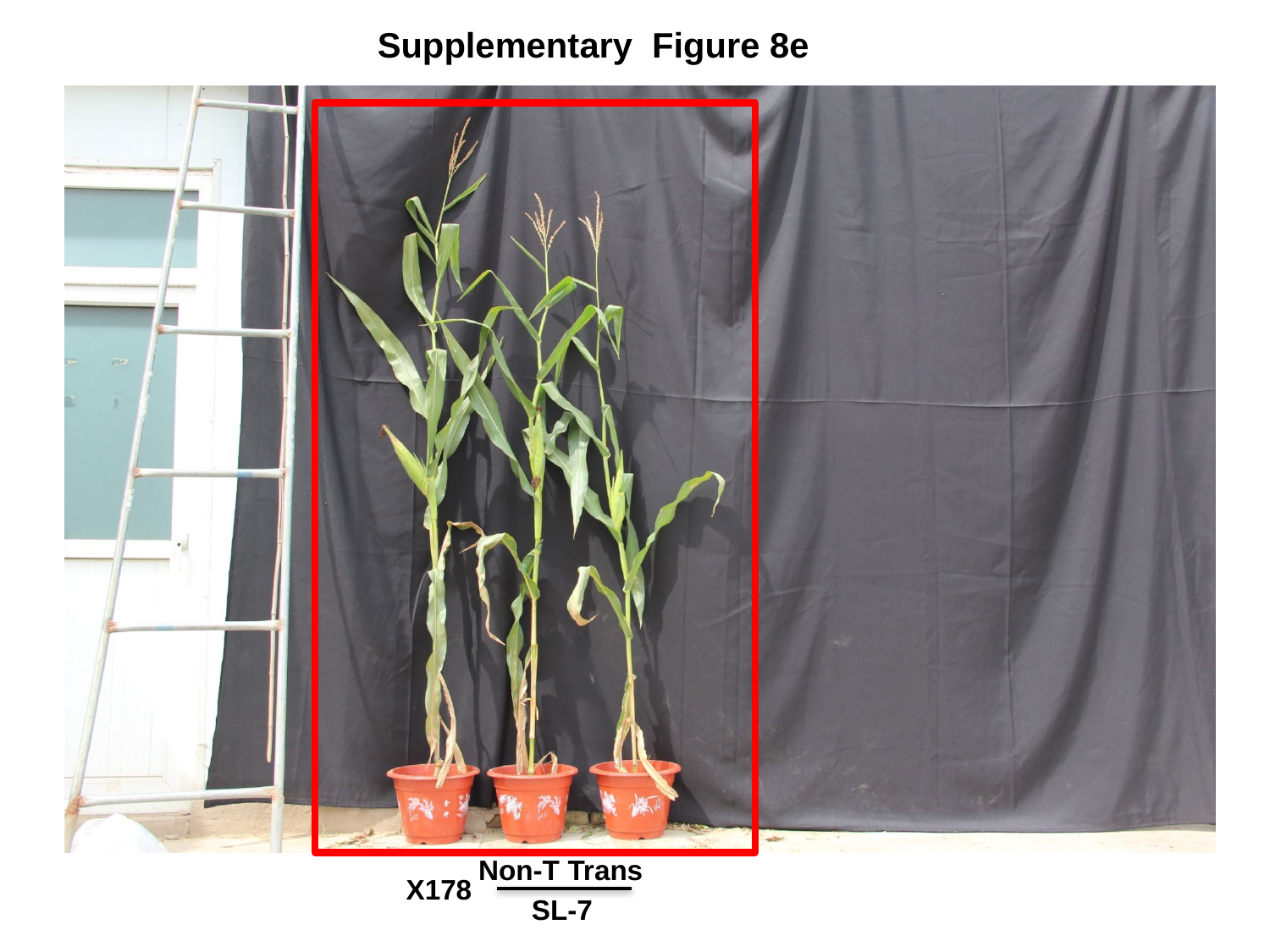

Supplementary Figure 8e
Non-T
Trans
SL-7
X178
