# Supplementary material for: A helitron-induced RabGDIα variant causes quantitative recessive resistance to maize rough dwarf disease
Source: Nat Commun. 2020 Jan 24;11:495. doi: 10.1038/s41467-020-14372-3 (PMC6981192; doi:10.1038/s41467-020-14372-3)
Supplement: Supplementary file 13 — Source Data [file 41467_2020_14372_MOESM13_ESM.zip › Supplementary Figure 11b.pptx]

## Slide 1
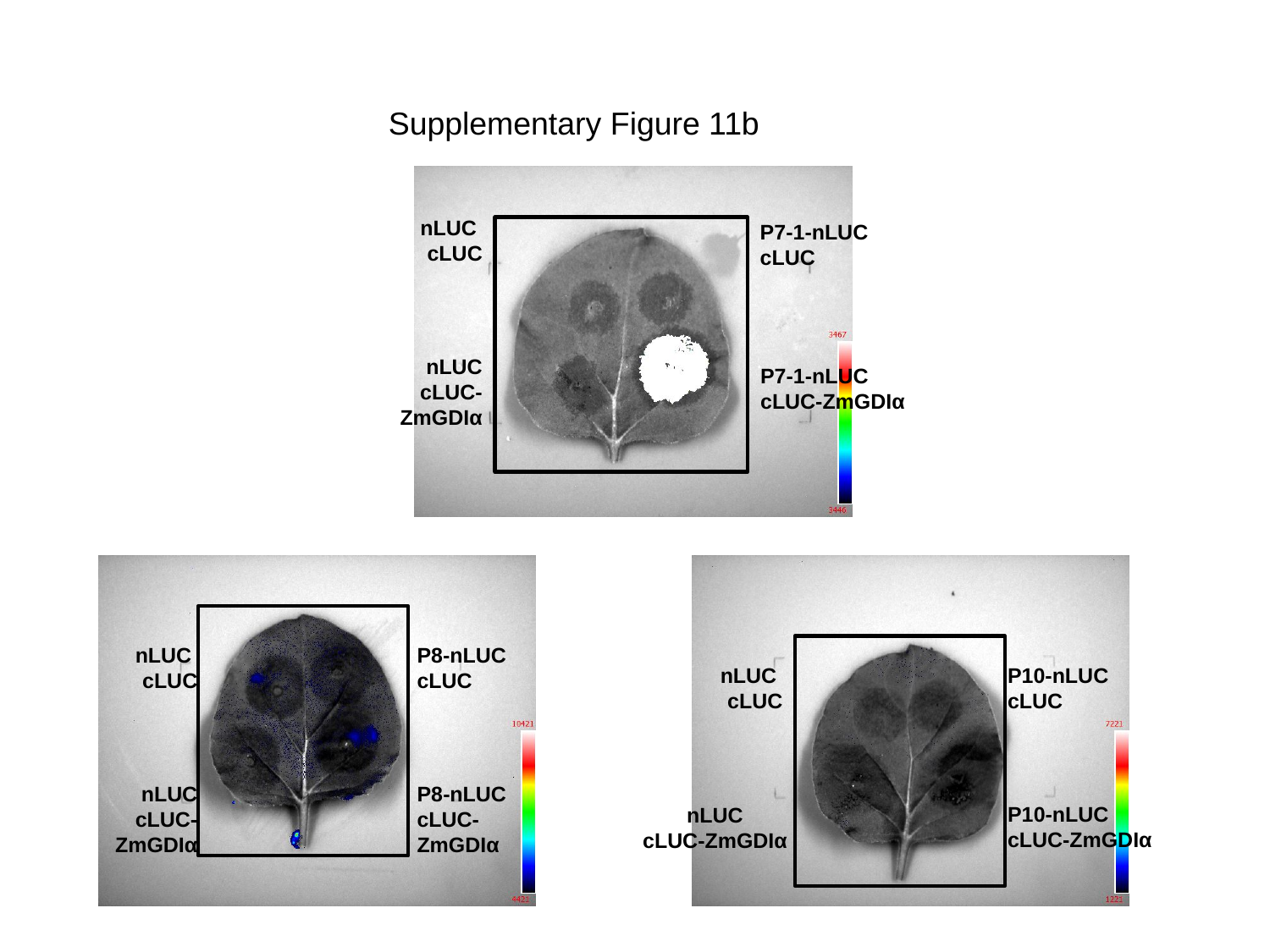

Supplementary Figure 11b
nLUC
cLUC
P7-1-nLUC
cLUC
nLUC
cLUC-
ZmGDIα
P7-1-nLUC
cLUC-ZmGDIα
nLUC
cLUC
P8-nLUC
cLUC
nLUC
cLUC-
ZmGDIα
P8-nLUC
cLUC-
ZmGDIα
nLUC
cLUC
P10-nLUC
cLUC
P10-nLUC
cLUC-ZmGDIα
nLUC
cLUC-ZmGDIα
