# Supplementary material for: A helitron-induced RabGDIα variant causes quantitative recessive resistance to maize rough dwarf disease
Source: Nat Commun. 2020 Jan 24;11:495. doi: 10.1038/s41467-020-14372-3 (PMC6981192; doi:10.1038/s41467-020-14372-3)

Figure 2b


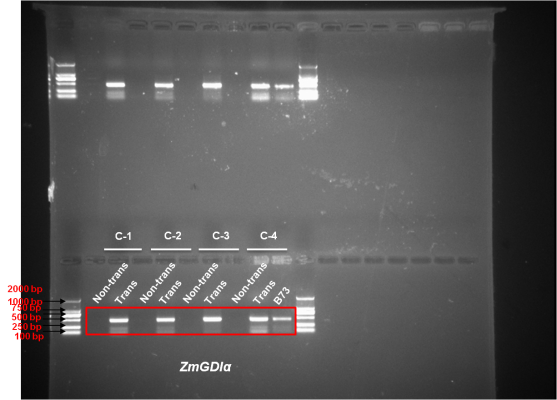

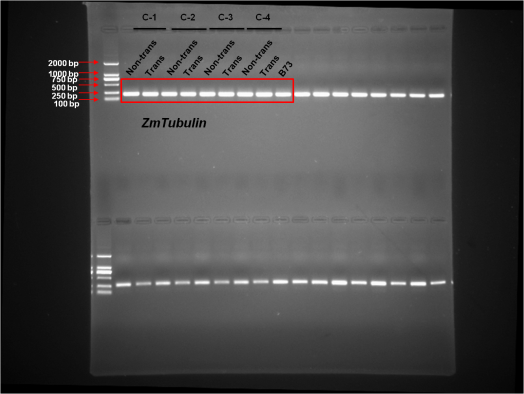


Figure 3a

**
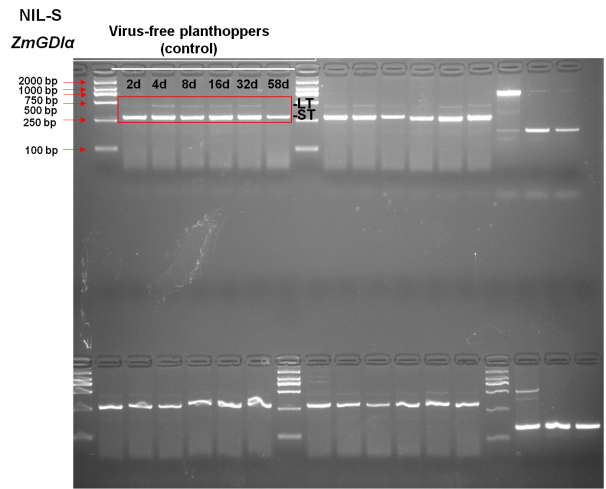

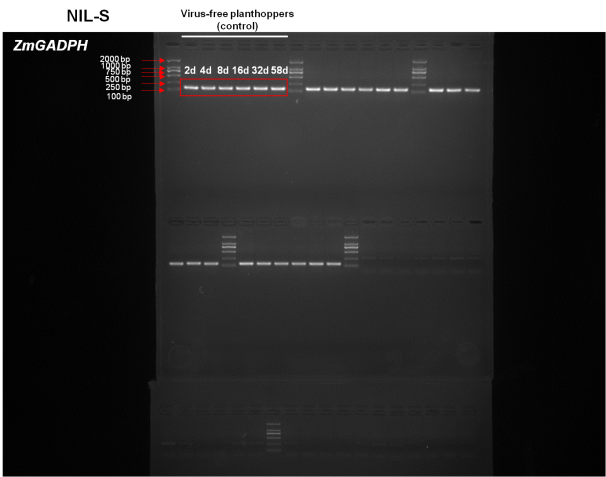
**

**
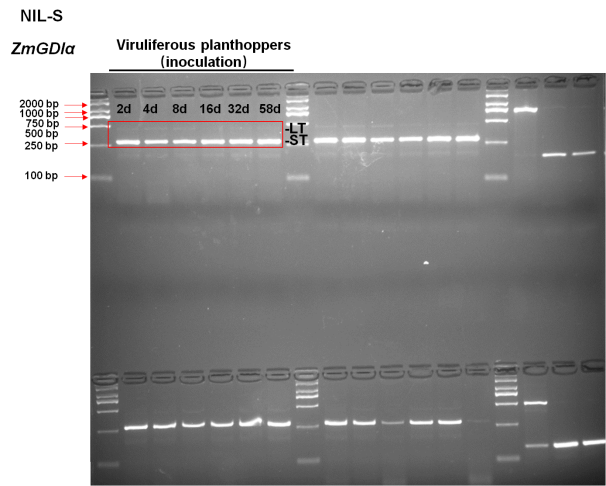

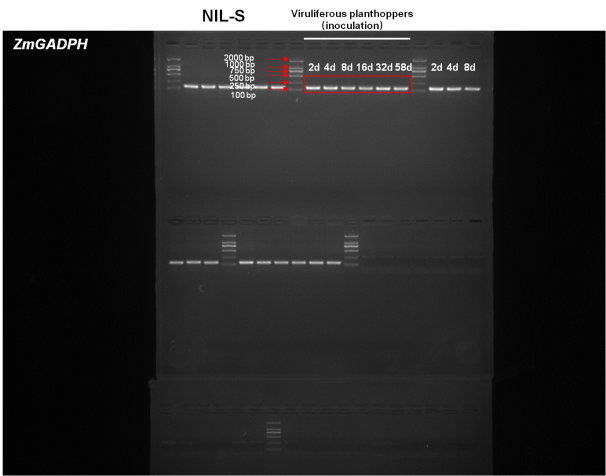
**

**
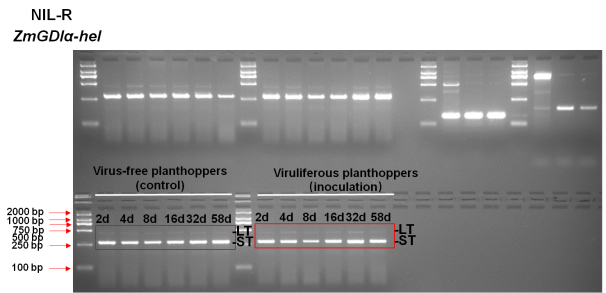

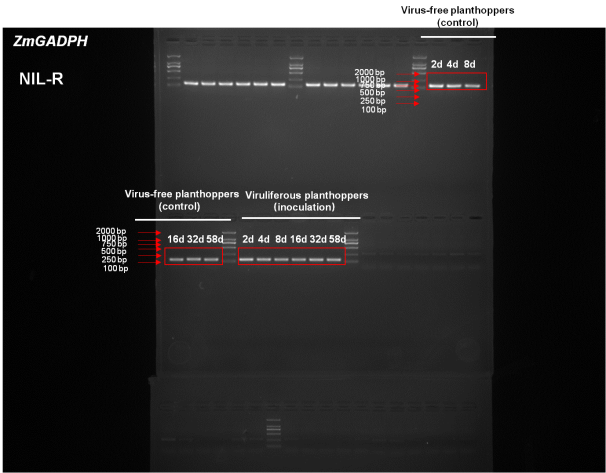
**

Figure 4b

**
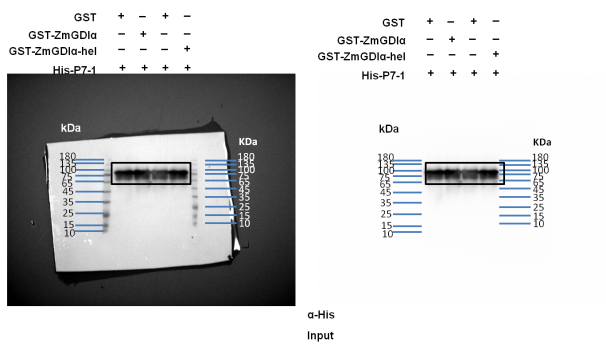

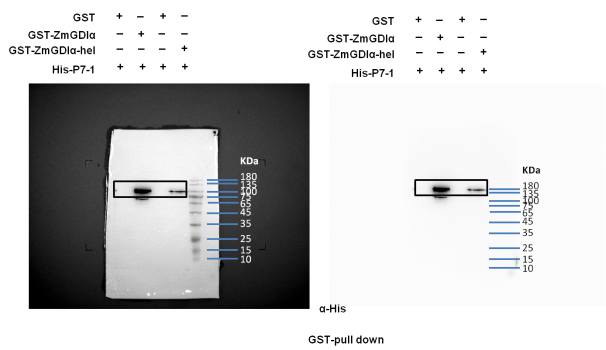
**

**
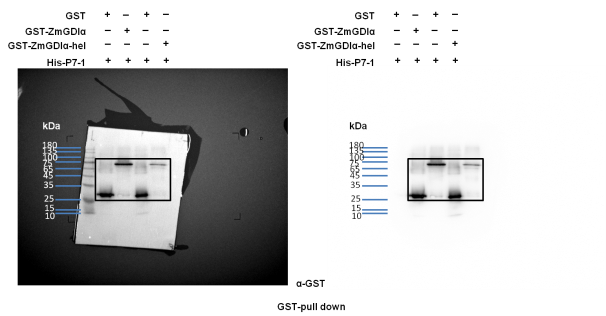
**

Figure 4c

**
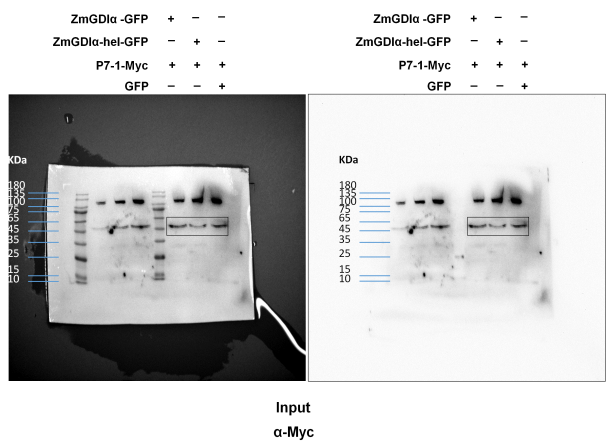

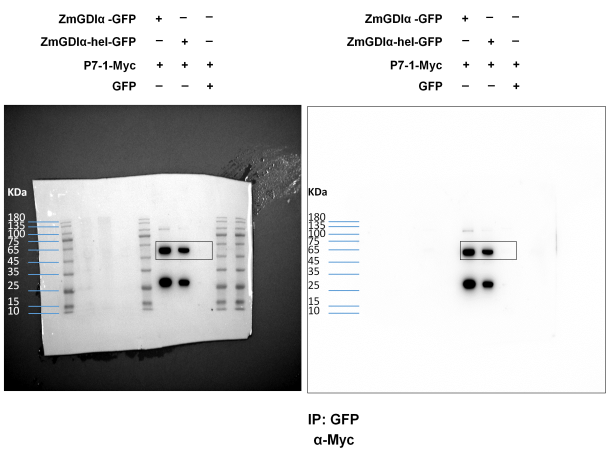
**

**
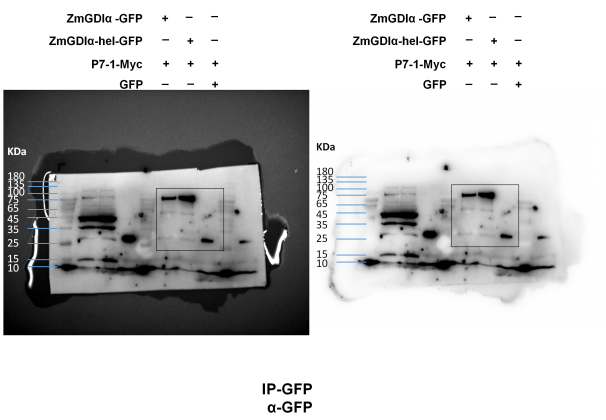

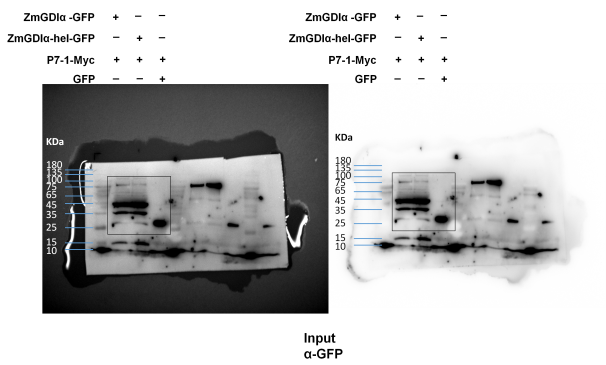
**

Figure 4d

**
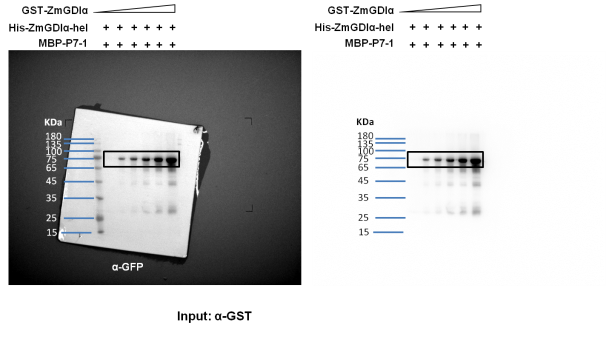

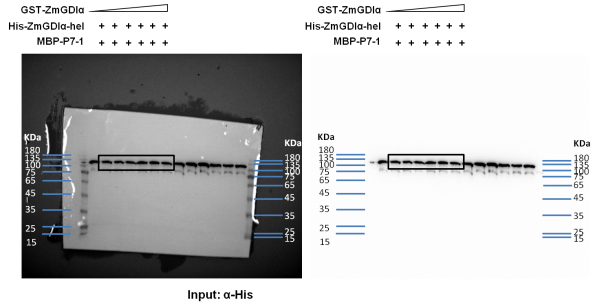
**

**
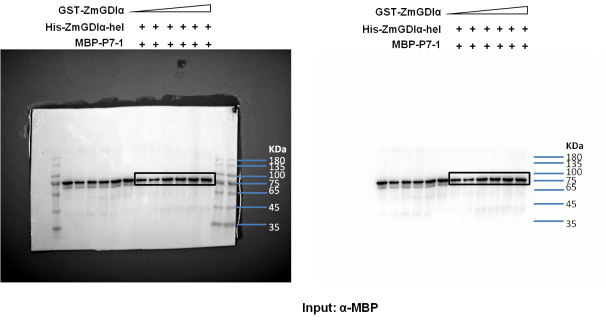

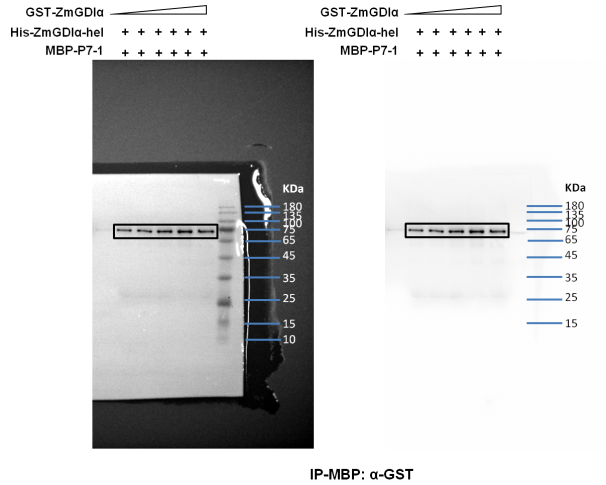
**

**
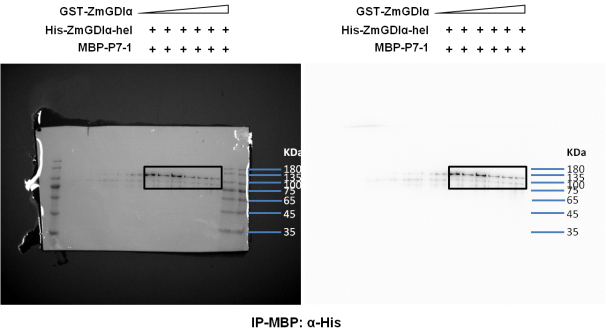
**

Figure 4e

**
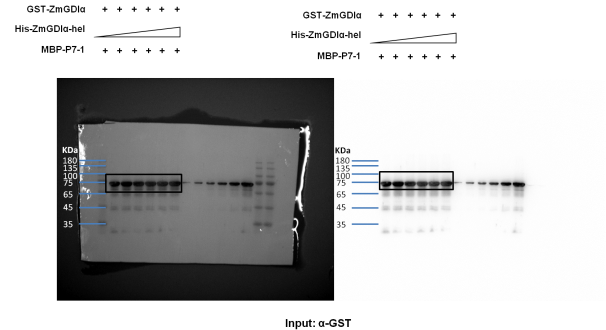

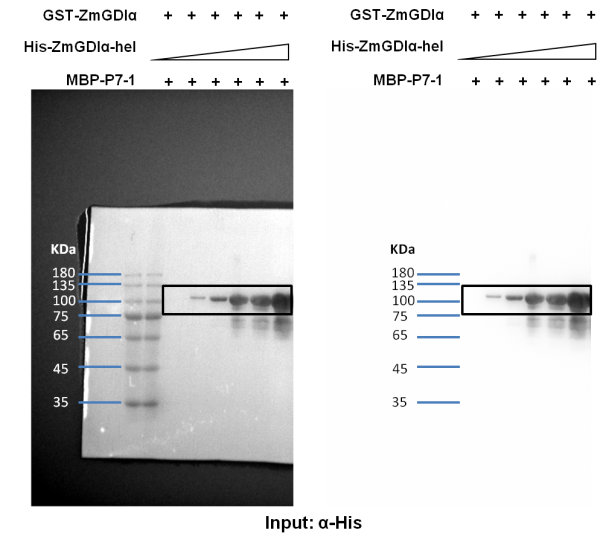
**

**
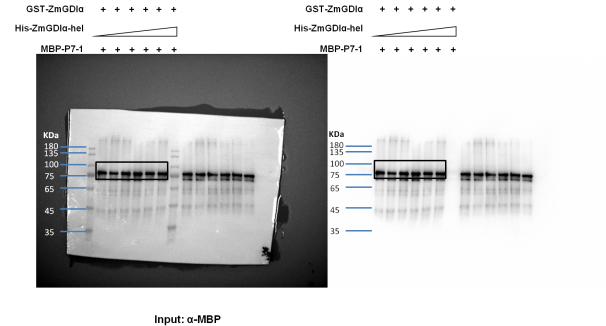

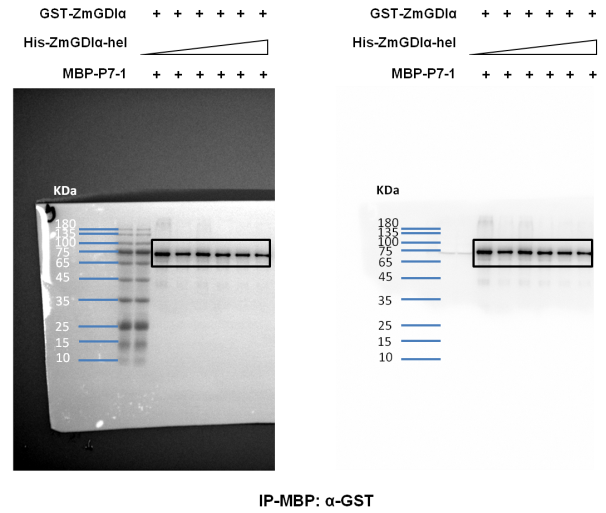
**

**
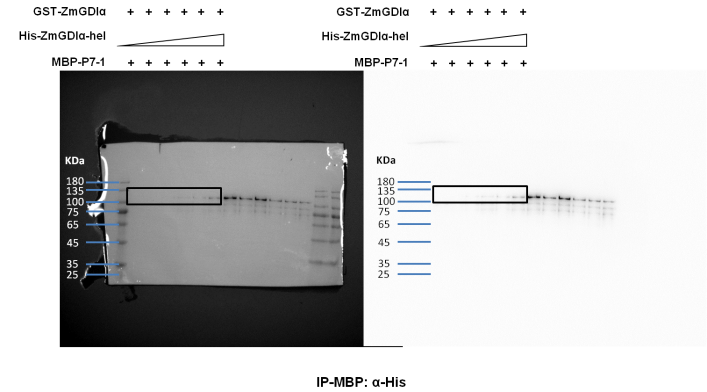
**

Figure 5e


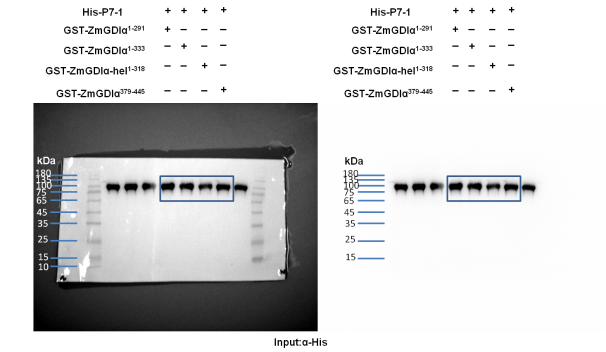

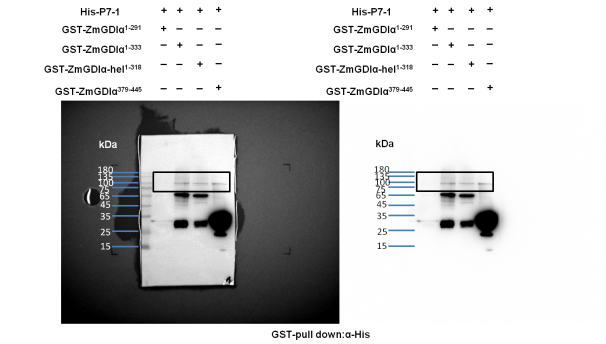


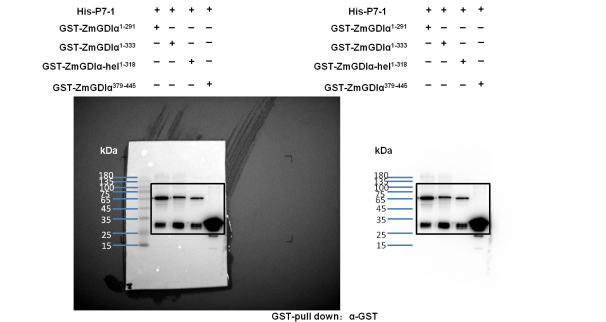


Figure 5f


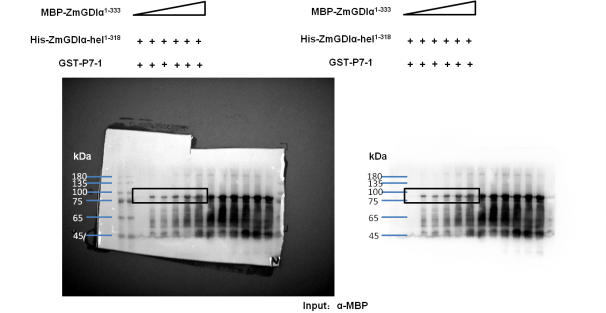

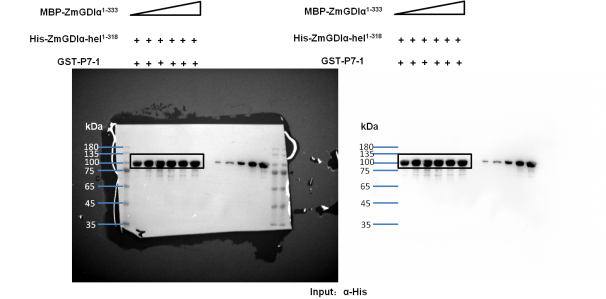


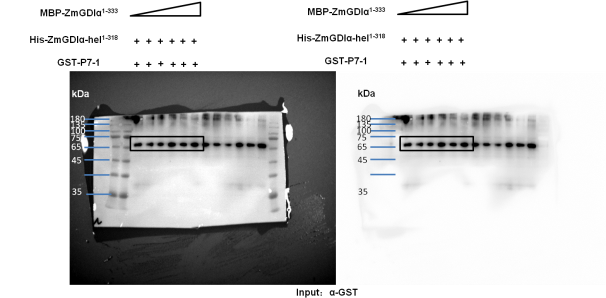

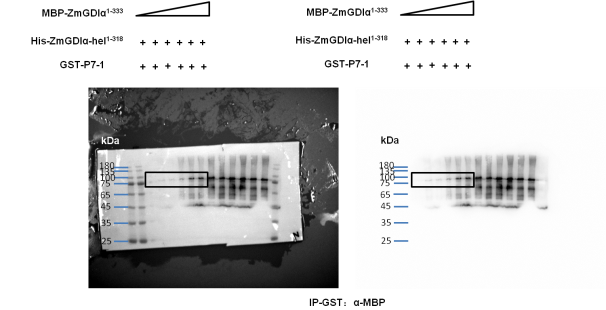


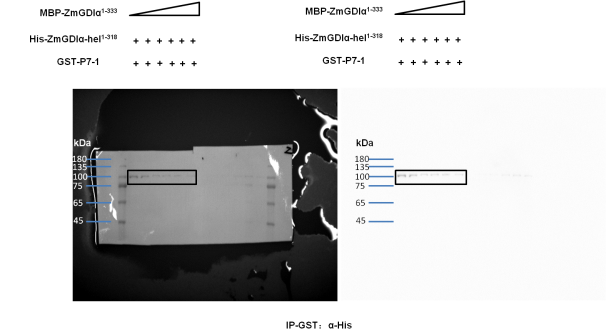


Figure 5g


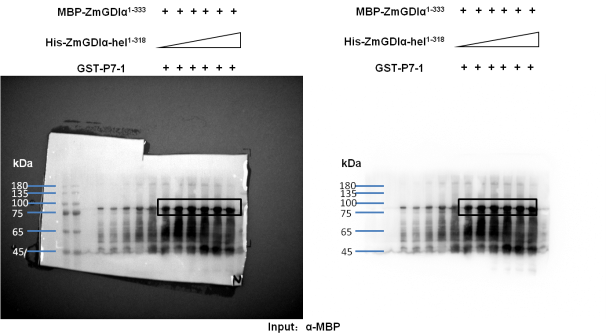

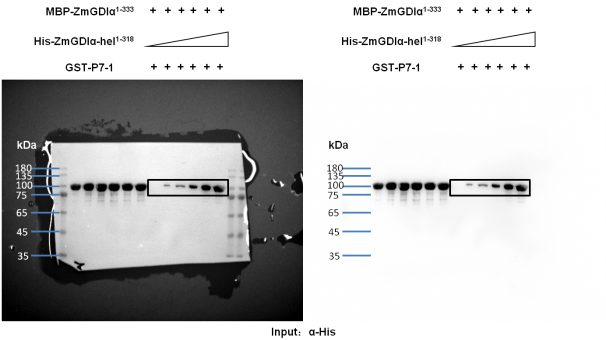


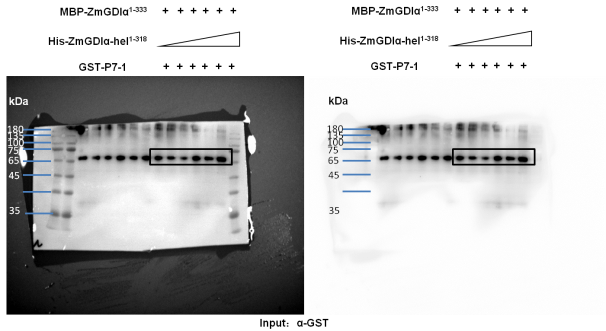

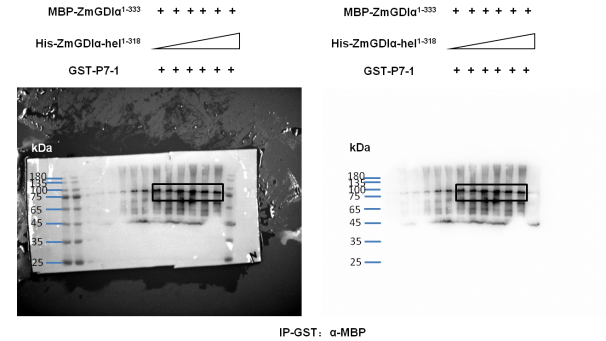


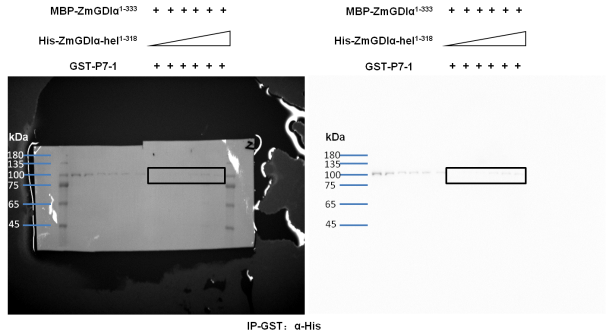


Supplementary Figure 4b


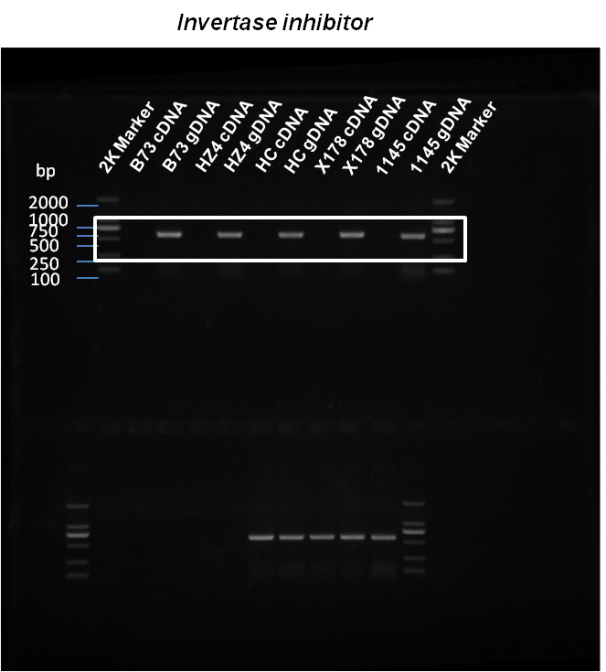

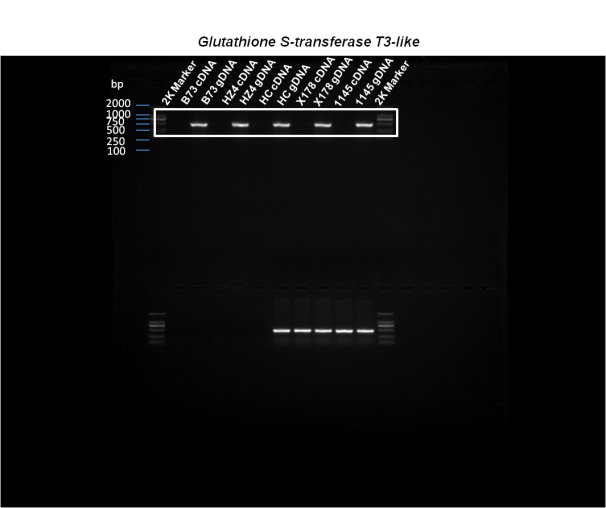


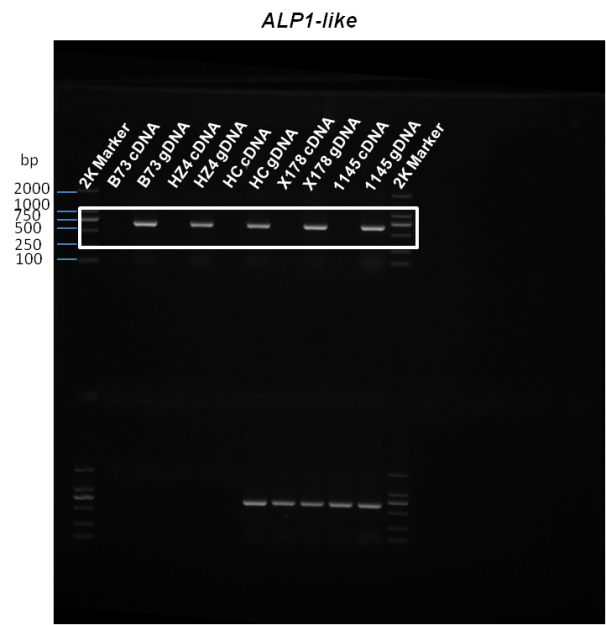

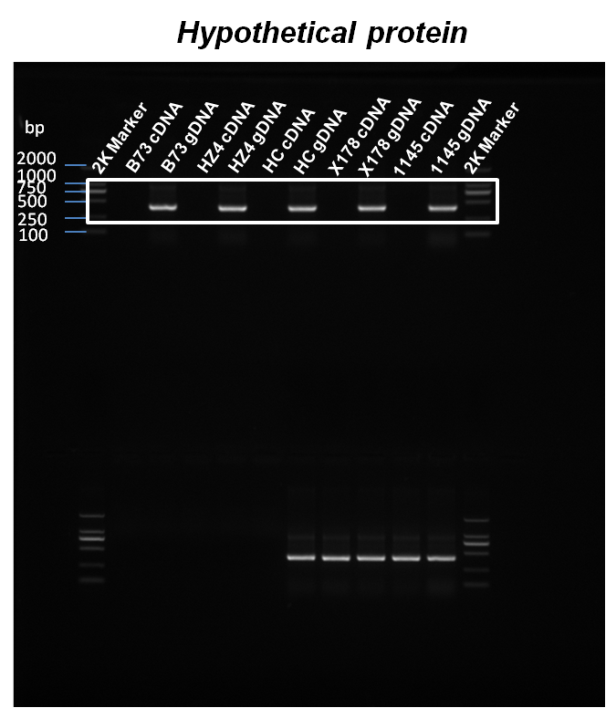


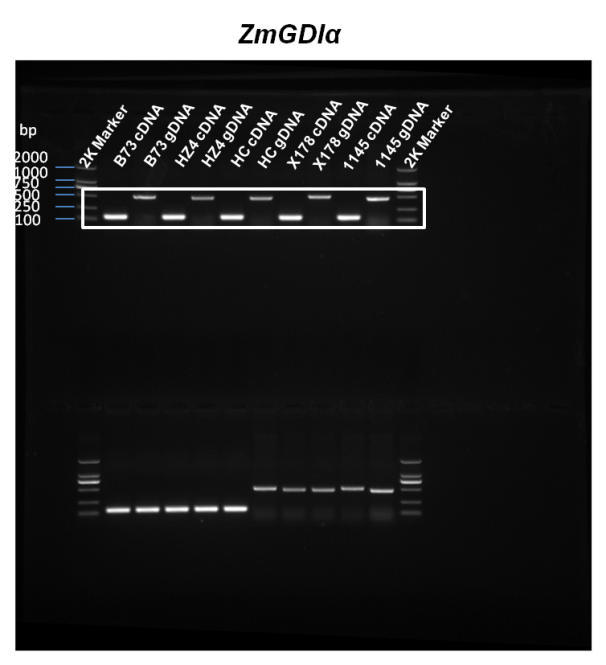

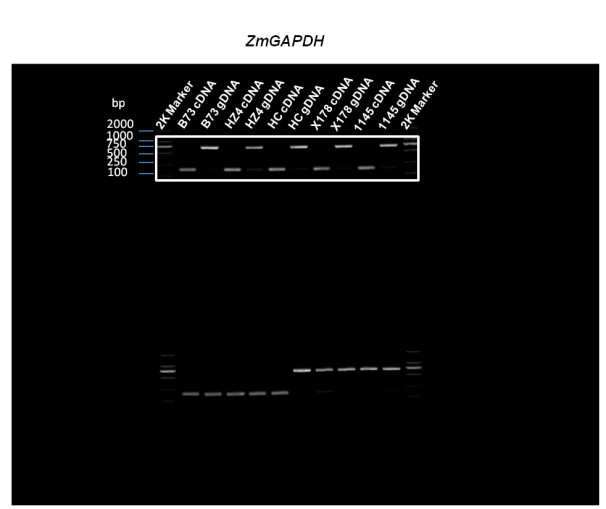


.

Supplementary Figure 7b

**
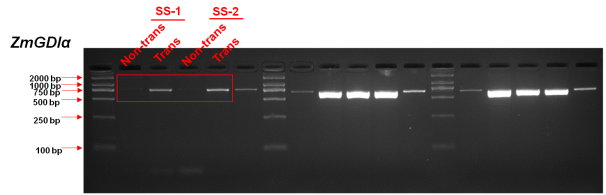

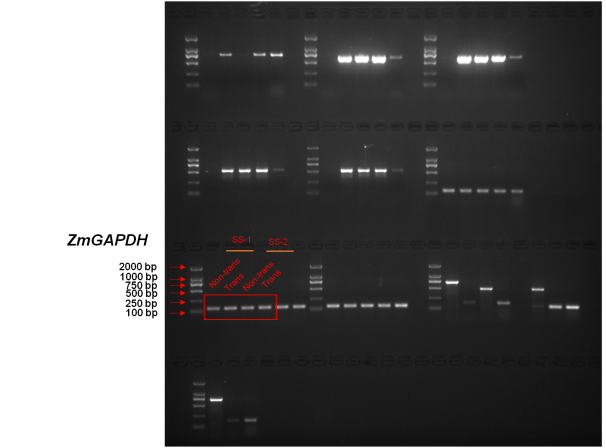
**

Supplementary Figure 7c

**
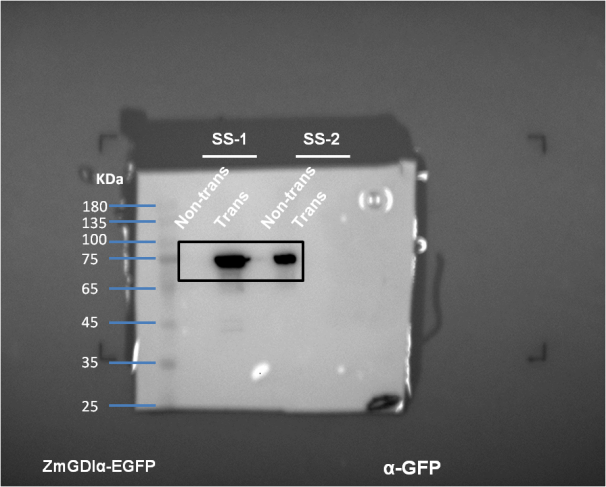

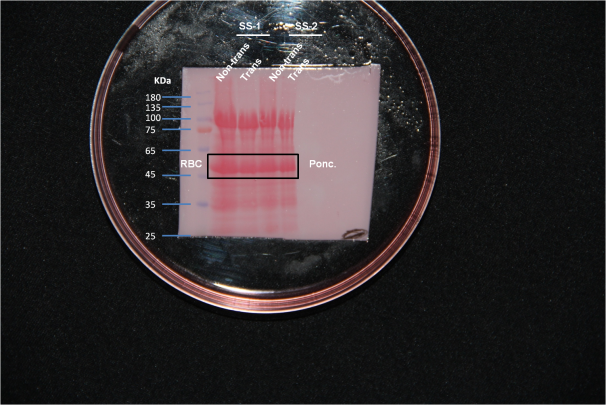
**

Supplementary Figure 8b


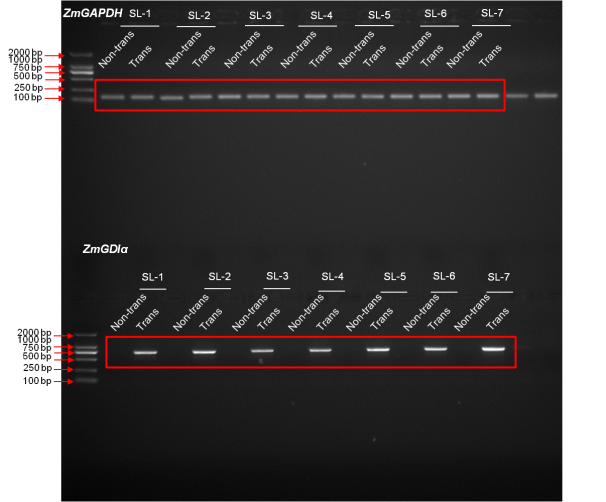


Supplementary Figure 8c


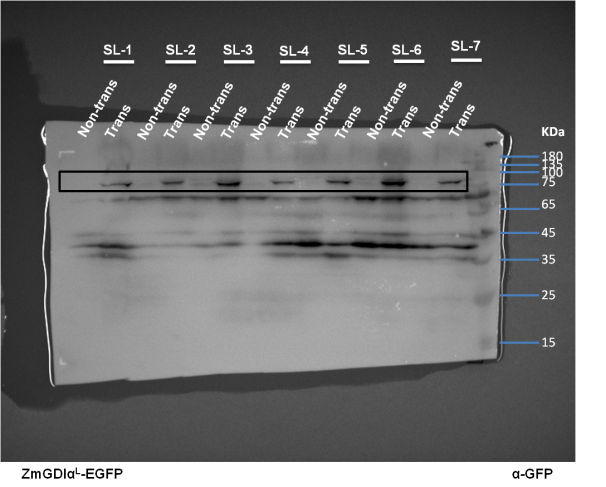


Supplementary Figure 9b


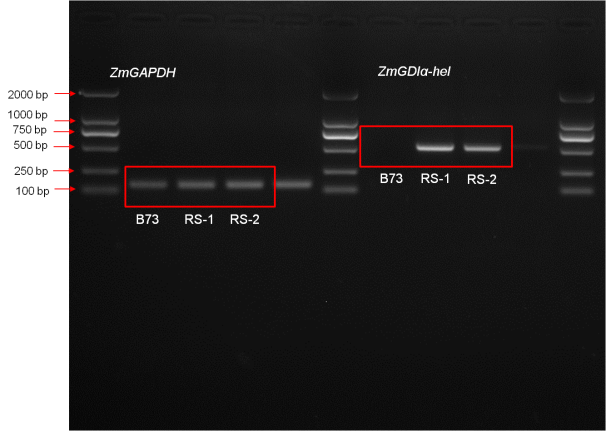


Supplementary Figure 9c


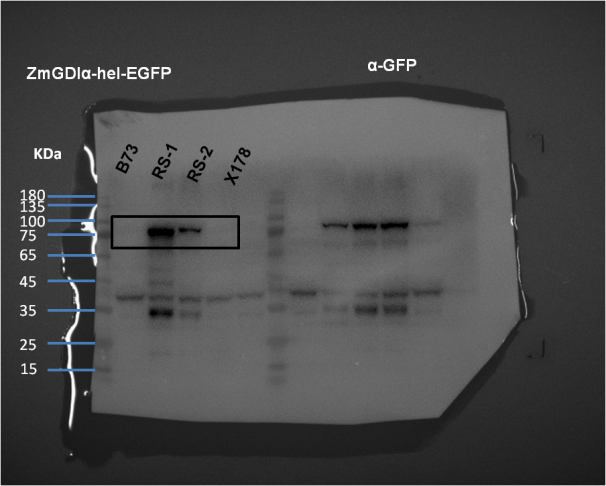

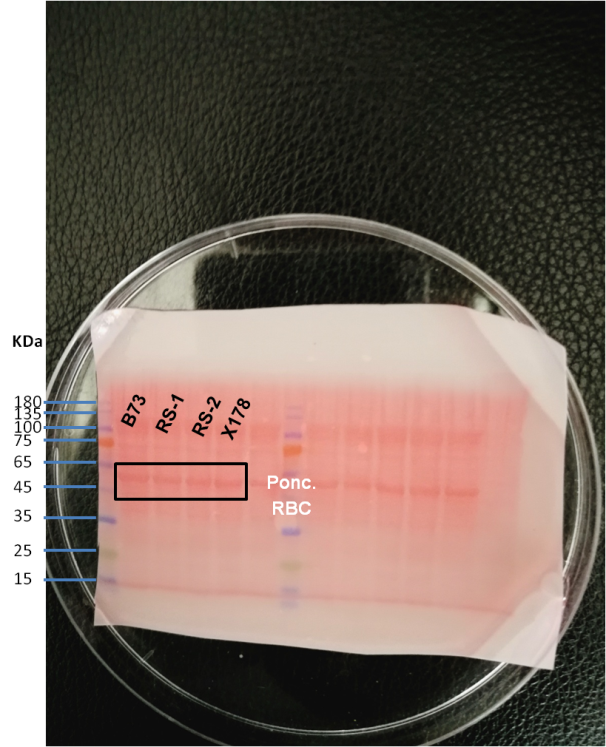


Supplementary Figure 9g


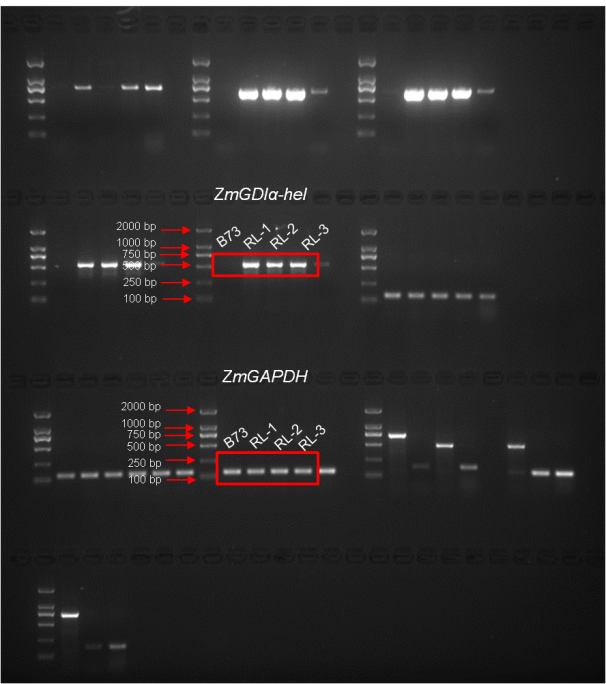


Supplementary Figure 9h


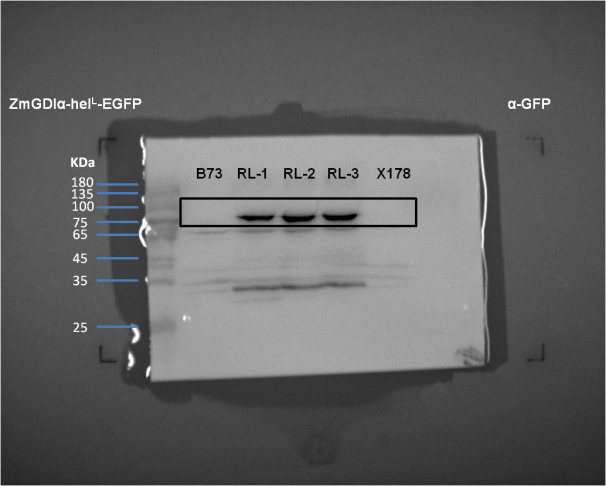

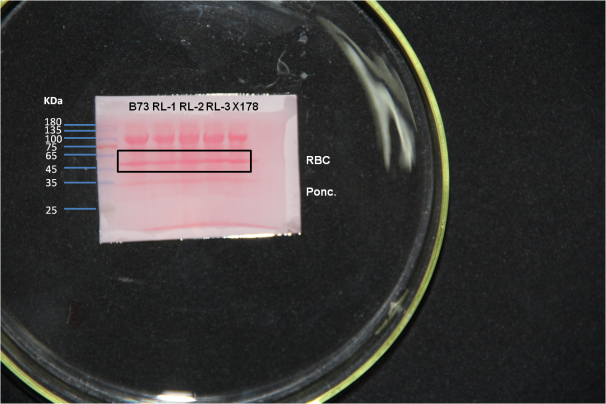


Supplementary Figure 11a


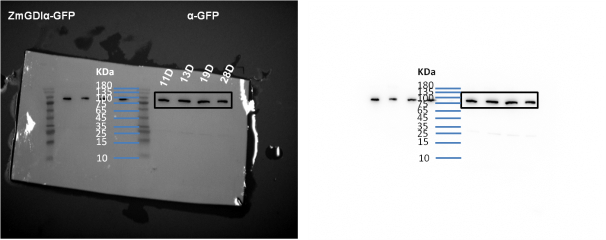


Supplementary Figure 11c


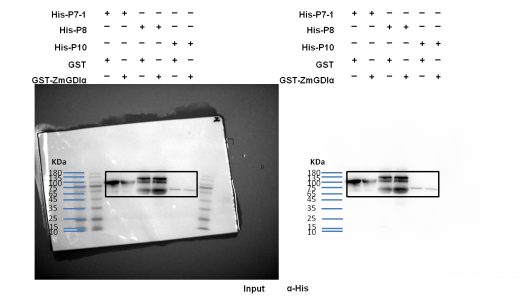

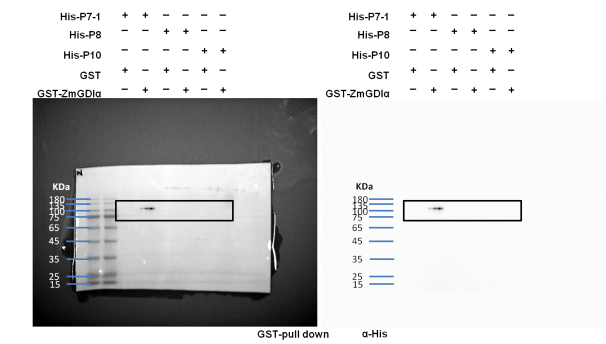


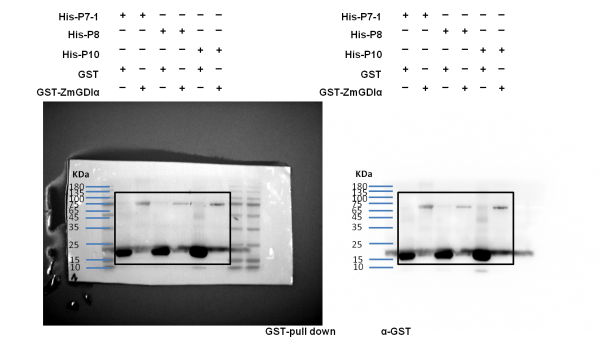


Supplementary Figure 16b


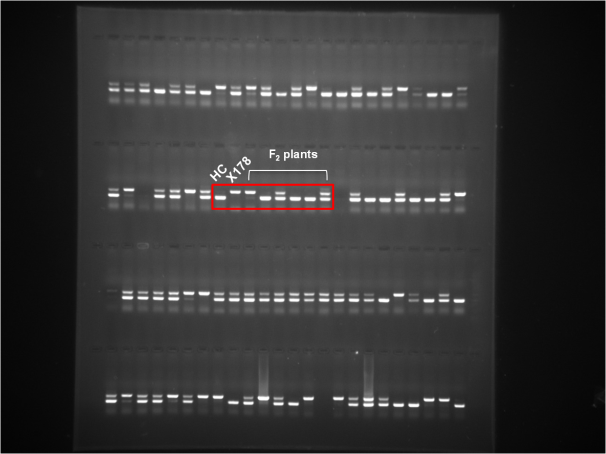

Supplement: Supplementary file 13 — Source Data [file 41467_2020_14372_MOESM13_ESM.zip › Figure 2b, 3a, 4b-e, 5e-g, Supplementary Figure 4b, 7b-c, 8b-c, 9b-c, 9g-h, 11a, 11c,16b.docx]
