# Supplementary figures and images for: A helitron-induced RabGDIα variant causes quantitative recessive resistance to maize rough dwarf disease
Source: Nat Commun. 2020 Jan 24;11:495. doi: 10.1038/s41467-020-14372-3 (PMC6981192; doi:10.1038/s41467-020-14372-3)

## Slide 1
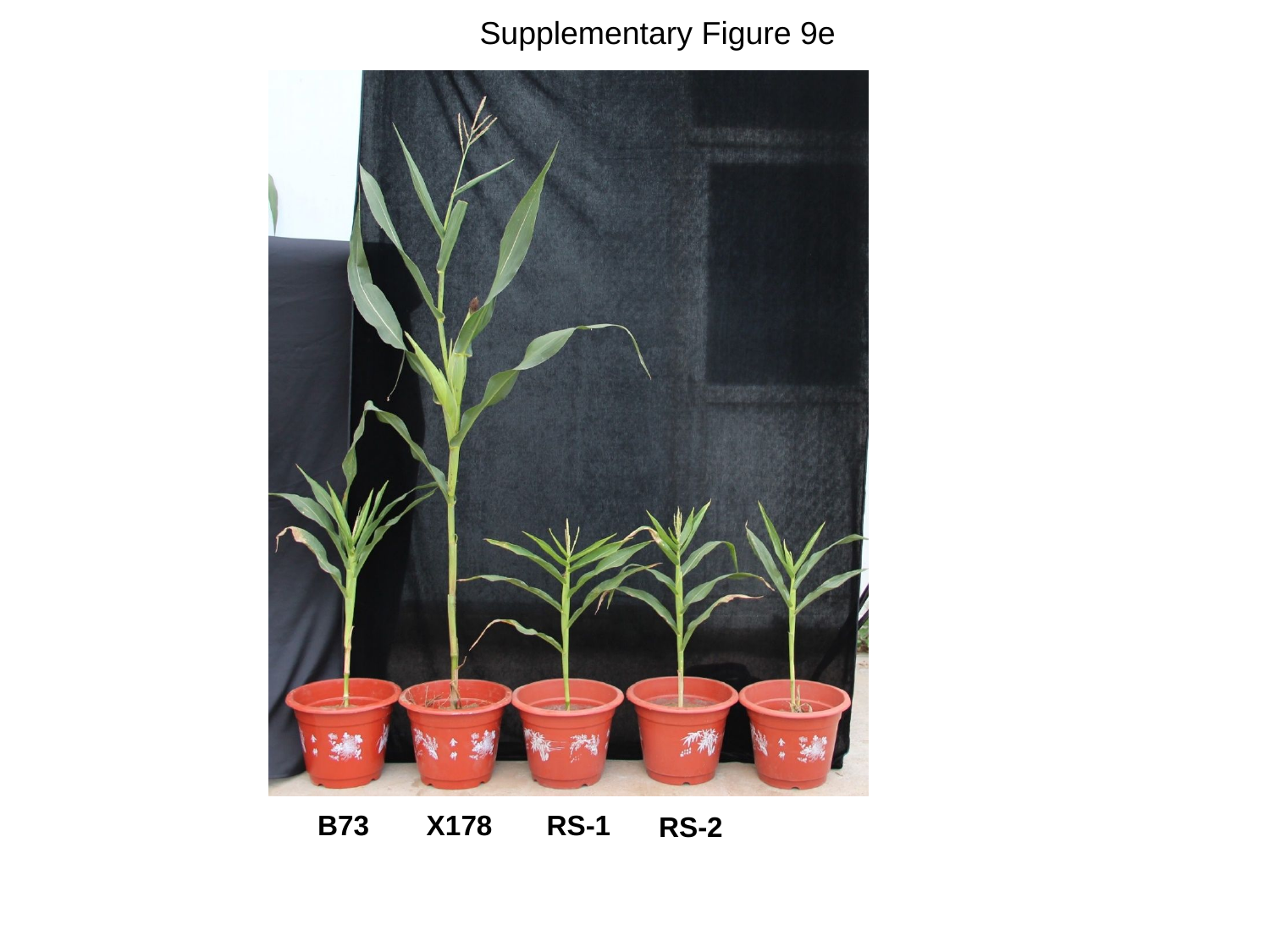

Supplementary Figure 9e
B73
X178
RS-1
RS-2

Supplement: Supplementary file 13 — Source Data [file 41467_2020_14372_MOESM13_ESM.zip › Supplementary Figure 9e.pptx]

## Slide 1
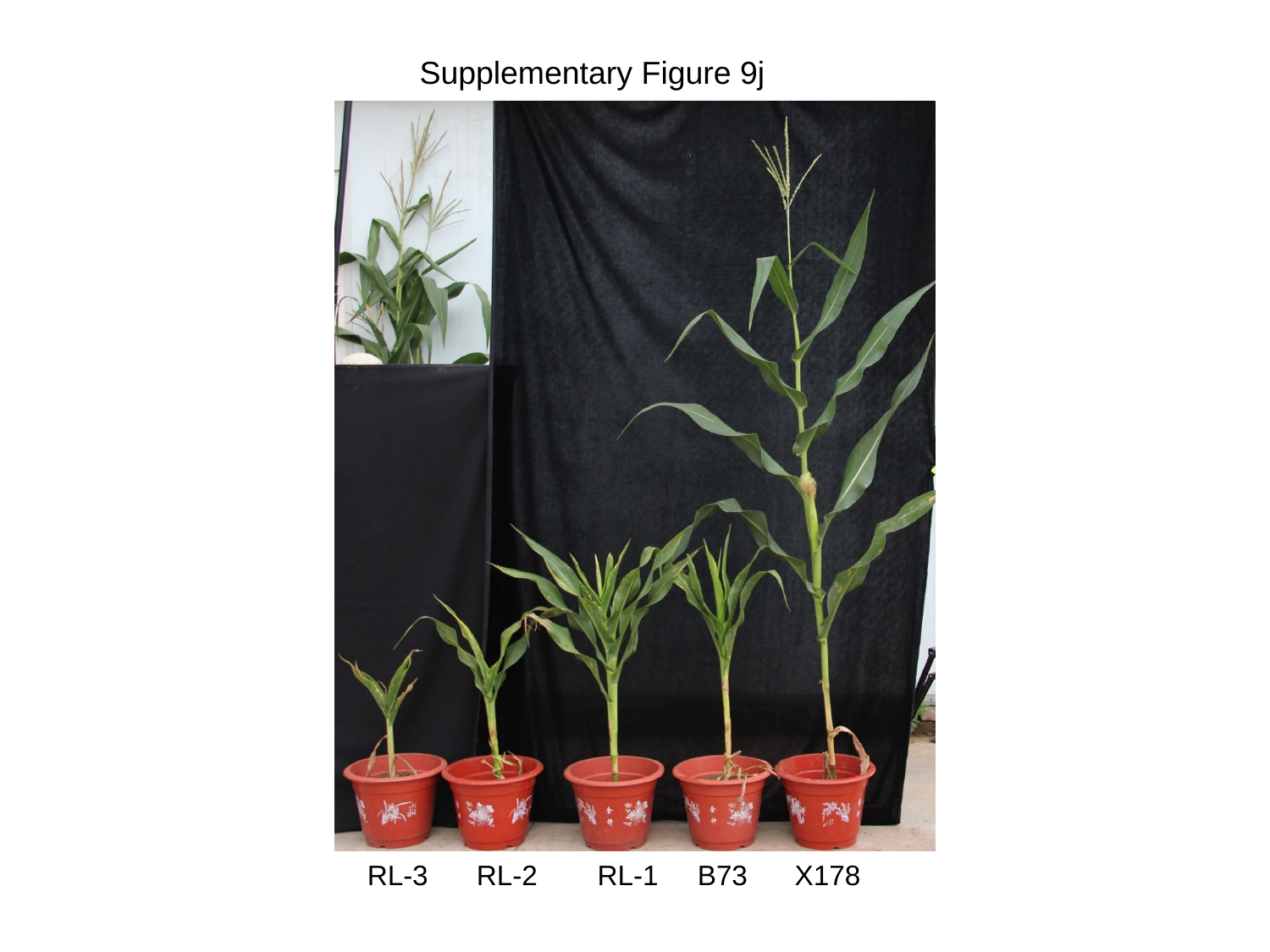

Supplementary Figure 9j
RL-3
RL-2
RL-1
B73
X178

Supplement: Supplementary file 13 — Source Data [file 41467_2020_14372_MOESM13_ESM.zip › Supplementary Figure 9j.pptx]
