# Supplementary material for: A helitron-induced RabGDIα variant causes quantitative recessive resistance to maize rough dwarf disease
Source: Nat Commun. 2020 Jan 24;11:495. doi: 10.1038/s41467-020-14372-3 (PMC6981192; doi:10.1038/s41467-020-14372-3)
Supplement: Supplementary file 2 — Peer Review [file 41467_2020_14372_MOESM2_ESM.pdf]

Reviewers' comments:

Reviewer #1 (Remarks to the Author):

This manuscript describes the molecular isolation and characterization of the gene underlying the qMrdd1 quantitative locus conferring partial, recessive resistance to the maize rough dwarf virus in the maize crop plant. The gene was identified by a classical map-based cloning approach as the Rab GDP dissociation inhibitor alpha protein which is the susceptibility factor for viral reproduction. A helitron transposon insertion into intron 10 results in alternative splice sites and different protein forms in the resistant and susceptible maize lines. This gene modification also explains the recessive genetic nature of resistance. P7-1 was identified to be the viral protein interacting with RabGDIalpha by split luciferase interaction assays as well as co-immuno precipitation experiments. There was a quantitative difference in binding of the viral P7-1 proteins by the two protein isoforms derived from alternative splicing in agreement with the quantitative resistance observed in the maize plant.

There are a number of recessive virus resistance genes described in plants. qMrdd1 represents a novel gene and, thus, a novel susceptibility factor for viral reproduction. Therefore, the presented work is of high novelty and describes a possibly generally present «Achilles heel » of the Fijiviruses. This knowledge has a large potential to identify resistance genes active against other plant Fijiviruses and for the molecular design of novel RabGDIalpha protein forms that lack the function needed for viral replication.

The finding that the critical insertion into the RabGDIalpa coding gene must have occurred after domestication adds to the small but growing list (mostly described in small grain cereals) of resistance genes that have evolved only after domestication, further corroborating that evolutionary events that occurred in agro-ecosystems have contributed important traits for crop plants.

The study is well described and the experimental data are convincing.

I have a number of minor concerns that must be addressed:

L117: High density molecular markers. This needs to be rephrased.

L152 and Figure 1 : It is not clear from the description if the two BAC clones from the resistant genotype contain the two closest flanking markers and if the contig is complete or contains gaps. This is relevant to determine if the list of identified candidate genes can be considered complete and to evaluate if there are additional, unknown genes in the region of interest.

I wonder if the authors tested tilling mutants or transposon insertion mutants in the RabGDIalpha gene and what was the outcome of such mutations on the viral interactions.

The discussion is a bit long and could be condensed.

Need for clearer description :
L57 : hm2: It is unclear what this means : hectares ?
L78 : replace ecological pollution by environmental pollution
L288 : rephrase « …showed no any interaction… »
L360 : conserved, not conservative
L375 : replace patch with domain.

Reviewer #2 (Remarks to the Author):

This is an interesting paper that describes characterization of a recessive resistance gene that confers resistance to RBSDV. The manuscript contains large amount of data and most of the results presented points to RabGDIa as a host factor required for RBSDV infection.

Specific comments:
Genetic analyses results clearly shows possible region that contains RBSDV resistance gene (Figure 1b). However, it is unclear how authors came to the conclusion that lack of expression of five putative genes in this region is an indication that these genes are not candidates for RBSDV resistance (Suppl Fig. 4b). Lack of expression could be due to several factors including expression level or primer issue. Also, authors state that there was no difference in expression of RabGDIa between resistant and susceptible line even though there is insertion of helitron TE. Did authors use primers that span exon 10 (where TE inserts)?

Functional validation:
Authors have generated number transgenic lines to demonstrate that RabGDIa is indeed the host factor that is required for RBSDV infection. However, some issues need to be resolved. Overexpression of GDIa even in the presence of GDIa-hel is sufficient to restore RBSDV infection but overexpression of GDIa-hel in the background of GDIa has no effect on the infection. If this is due to titration effect, in the latter case titration does not work. Authors should provide some explanation. Confounded by these results is that RNAi of GDIa has no effect on susceptibility. A conclusive experiment would have been to delete GDIa-hel in resistance line or truncate GDIa to delet exon 10 in B73 susceptible line using CRISPR. This reviewer is aware that this is a major undertaking in maize.

Molecular characterization:
Based on multiple assays presented in Fig. 4, it seems both GDIa and GDIa-hel interact with viral protein P7-1. Furthermore, GDIa can compete for interaction with P7-1 in the presence of GDIa-hel. How does this result translate in plants? In resistant plants only protein present is GDIa-hel. Since this can also interact with P7-1, how does this affect viral infection?

In vivo co-IP experiment should include a negative control (Fig. 4c). Also band in a-Myc probed IP blot is barely visible. Why there is excess amount of GDIa-GFP in this blot?

Similar issue also observed when GDIa(1-333) and GDIa-hel(1-318) are tested in luciferase complementation and in vitro pull down assays (Fig. 5). GDIa-hel(1-318) can be out competed when GDIa(1-333) is at higher concentration. Therefore, these results are not sufficient to make the conclusion "The results showed that P7-1 bound more tightly to ZmGDIα1–333 than to ZmGDIα-hel1–318 (Fig. 5f,g)" - lines 305-306. Furthermore, in resistant lines only protein present is ZmGDIa-hel; this can bind at similar strength to P7-1 as GDIa (see Fig. 4 and 5 in vitro pull-down data). Therefore, is binding alone is responsible for imparting resistance or susceptibility?

Authors could use transgenic lines expressing GDIa-EGFP and GDIa-hel-EGFP to see if there is different strength of P7-1 binding by MS analyses (similar to that they did to identify viral proteins that interact with GDIa - Suppl Fig. 12a and Suppl Table 3).

Reviewer #3 (Remarks to the Author):

Liu et al. report a great deal of work fine-mapping and showing the single gene that seems to be the sole contributor to recessive quantitative resistance to maize rough dwarf disease in maize. The authors also show that the resistant allele at this locus has an insertion that interferes with interaction with the RBSDV P7-1 protein, and propose a model for why this interaction may be important during virus infection. The conclusions and extensive screening of maize germplasm for alleles are of great

interest to maize virologists and breeders, as MRDD is an important viral disease. Despite the volume of work shown here, much work remains to be done to elucidate the mechanism by which this interaction confers resistance. I also question how impactful this work will be in the end, since the identified resistance is both recessive (and most commercial maize is hybrid, requiring both inbred parents to have the recessive allele), and quantitative and therefore does not eliminate infection or inoculum source from plants. Thus while it represents a major advance in cloning the gene and identifying a virus interaction that appears to be critical (and I fully appreciate that doing reciprocal mutagenesis on the virus side is not within reach for this virus), I am on the fence about how impactful this discovery his for the intended audience here. (In my opinion, cloning of broadly used potyvirus resistance in maize and beginning to understand its mechanism of action is higher impact as resistance is dominant and nearly complete).

The manuscript shows carefully conducted work that appears to be very thorough in presentation and meticulous in completion, so I did not uncover any errors creating scientific qualms about the reported findings. Consistency in nomenclature (including defining term usage early) and streamlining the manuscript would greatly improve its accessibility. For example, the authors seem undecided about how to refer to the virus and disease, with "maize rough dwarf virus disease" in the title, "Maize rough dwarf viral disease" in the first line, and variations on this throughout. The correct nomenclature is "maize rough dwarf disease", MRDD. Terms such as passive resistance should be explained at the beginning rather than at the end, along with crucial background information about the nature of resistance and the extent of quantitative contribution that are described in detail only at the end in the current manuscript. Wording errors such as L43 "threatening world food safety" should be corrected (security?), and the writing can certainly be streamlined.

Finally, the number of supplementary figures and tables is staggering, even for a Nature contribution, and some streamlining and/or consolidation would assist in orienting readers. Some detailed suggestions on Figures and Table consolidation and incorporation of key data into the main figures rather than supplemental and vice versa are outlined below:

Fig. 1. Fine-mapping the MRDD resistance QTL qMrdd1. Unclear from legend. Are stunted plants infected with RBSDV?

Should show in fig. 1 the susceptible and resistant parents if they exist. What population/plants are a. and b. figures from?

Fig. 2. Define DSI in legend. Transgenic plants for ZmGDIalpha-EGFP have increased DSI percent? E. RBSDV copies: is there an absolute quantification of copy number?

Fig. 3. Expression profiles of ZmGDIa and ZmGDAa-hel. Shows no change in transcript levels between the two with or without inoculation.
*This could be supplemental data

Fig. 4. P7-1 binding to GDI and GDIa-hel. In a., why is first photo showing bleaching out? Meaning of white is not shown in high-low reference bar.

Fig. 5. Targeting regions or interacting regions?

Fig. 6. Model

Supplemental figures and tables:

Even for a Nature article, the number of supplemental figures and tables in addition to the main text figures is excessive. Needs focus on the most salient information.
Fig S1. Inaccurate title. Not really cytogenetics: this is cellular ultrastructure. I think that showing the

difference between NILs should be in the main manuscript, not supplemental data.
Fig. S2. Fine mapping qMrdd1
Fig. S3. More fine mapping: can it be combined with Fig. S2?
Fig. S4. Should probably be in main text rather than the current Fig. 3.
Fig. S5. Alignment of maize alleles
Fig. S6. Alignment of cDNAs
Fig. S7. Alignment of protein orthologs from various plants
Fig. S8. Phylogenetic analysis. Can this be included in a panel in a consolidated figure (e.g. with gene structure from Fig. S4 and summarized expression data)? Show branch lengths as well as branch support.
Fig. S9. Transgenic validation. Panel d. does not add much.
Fig. S10. Transgenic functional analysis of ZmGDIa-hel. The DSI panels do not add much—could be summarized more simply.
Fig. S11. RNA9-mediated functional assay. Again, DSI panels do not add much.
Fig. S12. Testing P7-1, P8, P10 for interaction with plant protein
Fig. S13. Alignment and predicted structures of alleles differing in susceptibility
Fig. S14. haplotypes
Fig. S15. Evolution prediction for resistant allele
Fig. S16. Tests of other natural alleles
Fig. S17. Breeding comparison. Could include in main text, perhaps combined with model figure (Fig. 6).
Table S1. Could be included with DSI description
Table S2. Primers
Table S3. Can data shown in Fig. S12 be combined into table?
Table S4. Again, duplicates some information show in Fig. S14. Suggest choosing one. “Unkown” typo throughout.
Table S5. Landraces. Can be consolidated with Table S4?
Table S6. Tseosinte panel tested for insertion.
Table S7. KHA test inputs.
Table S8. MRDD resistance inbreds.

Authors’ Response to Review Comments

Dear reviewers,

Thank you very much for the time that you spent on this manuscript. We were very happy to see that the comments were largely positive. Our detailed responses to all comments are below. We also checked the entire manuscript and made other modifications in the revised manuscript. Hopefully, you will be satisfied with them. We have uploaded the revised manuscript with tracked changes and other necessary documents. Any further comments will be greatly appreciated.

Sincerely Yours!

Mingliang Xu and co-authors

Reviewers' comments:

Reviewer #1 (Remarks to the Author):

This manuscript describes the molecular isolation and characterization of the gene underlying the *qMrdd1* quantitative locus conferring partial, recessive resistance to the maize rough dwarf virus in the maize crop plant. The gene was identified by a classical map-based cloning approach as the Rab GDP dissociation inhibitor alpha protein which is the susceptibility factor for viral reproduction. A *helitron* transposon insertion into intron 10 results in alternative splice sites and different protein forms in the resistant and susceptible maize lines. This gene modification also explains the recessive genetic nature of resistance. P7-1 was identified to be the viral protein interacting with RabGDIalpha by split luciferase interaction assays as well as co-immunoprecipitation experiments. There was a quantitative difference in binding of the viral P7-1 proteins by the two protein isoforms derived from alternative splicing in agreement with the quantitative resistance observed in the maize plant.

There are a number of recessive virus resistance genes described in plants. qMrdd1 represents a novel gene and, thus, a novel susceptibility factor for viral reproduction. Therefore, the presented work is of high novelty and describes a possibly generally present «Achilles heel » of the Fijiviruses. This knowledge has a large potential to identify resistance genes active against other plant Fijiviruses and for the molecular design of novel RabGDIα protein forms that lack the function needed for viral replication.

The finding that the critical insertion into the RabGDIα coding gene must have occurred after domestication adds to the small but growing list (mostly described in small grain cereals) of resistance genes that have evolved only after domestication, further corroborating that evolutionary events that occurred in agro-ecosystems have contributed important traits for crop plants.

The study is well described and the experimental data are convincing.

Response: We greatly appreciate the very positive comments of the reviewer and would like to take this opportunity to thank the reviewer for the constructive suggestions on our MS.

I have a number of minor concerns that must be addressed:

Q1: L117: High density molecular markers. This needs to be rephrased.

A1: Thank you very much for the comment. We replace “high-density molecular markers” with “a set of molecular markers”, and this should be more accurate in description of those newly-developed markers. Please refers to Line 125 in the revised MS.

Q2: L152 and Figure 1: It is not clear from the description if the two BAC clones from the resistant genotype contain the two closest flanking markers and if the contig is complete or contains gaps. This is relevant to determine if the list of identified candidate genes can be considered complete and to evaluate if there are additional, unknown genes in the region of interest.

A2: Thank you very much for the remainder. It is our carelessness to miss some important points in description of the BAC clones. Two overlapping BAC clones, 301B2-1 and 53A2-9, screened from the resistant 1145 BAC library contain the left and right flanking markers S18 and K7-1, respectively. They form a BAC contig that completely covers the mapped 105-kb *qMrdd1* region without any gaps (Supplementary Figure 4a). Thus, the list of the candidate genes is complete and no any candidate gene is missing. We modified the sentence to give an unambiguous description of the BAC contig. Please refers to Line 160 in the revised MS.

Q3: I wonder if the authors tested tilling mutants or transposon insertion mutants in the RabGDIα gene and what was the outcome of such mutations on the viral interactions.

A3: Yes! When searching for the MEMD (http://www.elabcaas.cn/memd/index.php), we identified three EMS mutants (EMS4-0a962f; EMS4-0a9630 and EMS4-0a963b) with nonsynonymous base-pair substitutions in the *RabGDIα* gene (Table R1**,** attached below). These mutants were propagated in Hainan Winter Nursery, and inoculated with RBSDV in Beijing in 2019. Consequently, we found all mutant plants were severely infected by MRDD (Figure R1, attached below). Of the three mutants, the first two are located on the P7-1-targeting C-terminal region (exon 11), it is speculated that these two amino-acid changes would not impair P7-1 interacting with C-terminal region. The third one resides on exon 1, outside of two P7-1-targeting regions (exon 10 and C-terminus). Thus, these amino-acid changes are not expected to weaken P7-1 in recruitment of RabGDIα to alleviate viral infection.

Table R1. The detail information of EMS mutants

Mutant ID	Location in chromosome	Position in gene	Effect	Codon change	Amino acid Change	Location of amino acid changes
EMS4-0a962f	8:106234656	**Exon11**	NON_SYNONYMOUS_CODING	Cca/Tca	P/S	**ZmGDIα**^{394}
EMS4-0a9630	8:106234665	**Exon11**	NON_SYNONYMOUS_CODING	Gtc/Atc	V/I	**ZmGDIα**^{391}
EMS4-0a963b	8:106242155	**Exon1**	NON_SYNONYMOUS_CODING	Gag/Aag	E/K	**ZmGDIα**^{15}

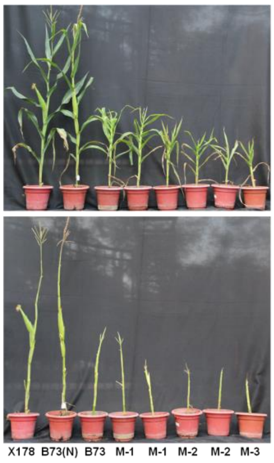

Figure R1. The resistance performance of mutant plants. All mutant plants were inoculated at the two-leaf stage with RBSDV and investigated their resistance performance at 90 dpi. X178: resistant control; B73(N): non-inoculated B73 line; B73: inoculated B73 line; M-1: inoculated EMS4-0a962f; M-2: inoculated EMS4-0a9630; M-3: inoculated EMS4-0a963b.

Q4: The discussion is a bit long and could be condensed.

A4: Thank you for the suggestion. We revised the discussion section and deleted those

unnecessary or redundant descriptions. Please see the revised discussion.

Need for clearer description:

Q5: L57: hm2: It is unclear what this means: hectares?

A5: Here Hm^2 means hectares, we have replaced hm^2 with hectares in the revised version. Please refer to Lines 63-64.

Q6: L78: replace ecological pollution by environmental pollution

A6: Thank you for the suggestion. We have replaced “ecological” with “environmental”, Please refer to Line 84 in the revised MS.

Q7: L288: rephrase « …showed no any interaction… »

A7: Thanks! We have rewritten the sentence as “The N-terminus upstream of exon 10 (ZmGDIα$^{1–291}$) didn’t interact with P7-1, but the C-terminus downstream of exon 10 (ZmGDIα$^{334–445}$) did show an interaction with P7-1 (Fig. 5c)”. Please refer to Line 312 in the revised MS.

Q8: L360: conserved, not conservative

A8: Accepted and revised! Please refer to Lines 387-388 in the revised MS.

Q9: L375: replace patch with domain.

A9: Thank you! We just delete the term “a patch of”, and this makes the sentence more concise. Please refer to Line 402 in the revised MS.

Reviewer #2 (Remarks to the Author):

This is an interesting paper that describes characterization of a recessive resistance gene that confers resistance to RBSDV. The manuscript contains large amount of data and most of the results presented points to RabGDIa as a host factor required for RBSDV infection.

Response: We greatly appreciate the reviewer for the constructive comments and are willing to address all questions concerned.

Specific comments:

Q1: Genetic analyses results clearly shows possible region that contains RBSDV resistance gene (Figure 1b). However, it is unclear how authors came to the conclusion that lack of expression of five putative genes in this region is an indication that these genes are not candidates for RBSDV resistance (Suppl Fig. 4b). Lack of expression could be due to several factors including expression level or primer issue. Also, authors state that there was no difference in expression of RabGDIa between resistant and susceptible line even though there is insertion of helitron TE. Did authors use primers that span exon 10 (where TE inserts)?

A1: Thank you for your comments. We fully understand the reviewer's concerns on the candidate genes for RBSDV resistance. We initiated this work more than 10 years ago and spend most of the time to refine the *qMrdd1* region. We've been trying to narrow *qMrdd1* down to a single candidate gene, but failed in the end. Within the final mapped 105-kb region, five no-transposon-related genes were predicted [notice: The fifth gene gag-pal precursor is associated with transposon LTR-retrotransposons (Dixit A, et al. 2006, Plant Cell Reports), and thus excluded in the revised version.

We agree that lack of expression could be due to several factors including expression level or primer issue. In the revised MS, we repeated the RT-PCR assay and found that all primers designed on exons of five candidate genes are capable to amplify genomic DNAs, but failed to detect gene expressions except *RabGDIa* (Supplementary Figure 4b, also attached below). We also tried to obtain full-size cDNA to construct overexpression vector. Again, we failed to obtain cDNAs of the candidate genes except

RabGDIa. It seems unlikely that a predicted gene having no gene expression and sequence variation is the candidate for *qMrdd1*. The next validation experiments indicated it is the *RabGDIa* gene that is associated with maize resistance to MRDD. Anyway, we delete the word 'only' in the sentence to avoid any misunderstanding. Please refer to line175 in the revised MS.

In RT-PCR, we designed primers flanking the extra exon 4 to differentiate the short and long transcripts for both *RabGDIa* and *RabGDIa-hel* alleles. In real-time RT-PCR, we once tried to use the primers flanking the exon 10, but they did not work well, then we switched to other primers suitable for real-time PCR to quantify total transcripts.

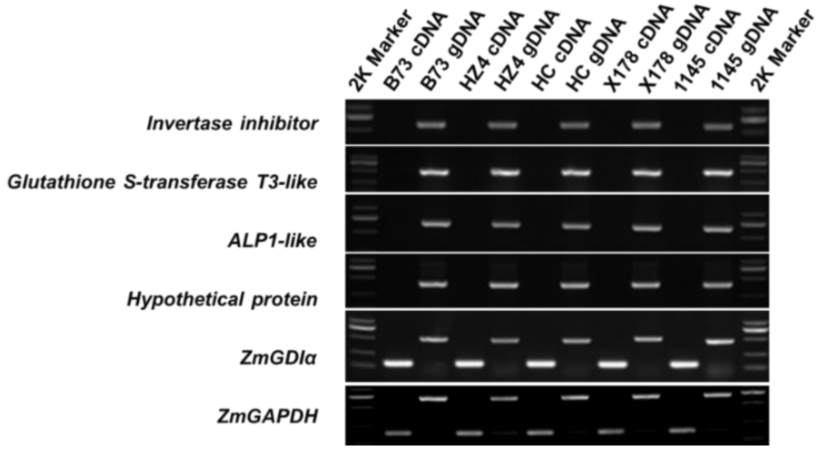

Supplementary Figure 4b. Gene expression assay for five predicted genes. Seedlings at the three-leaf stage were collected from resistant inbred lines 1145 and X178 as well as susceptible inbred lines B73, HZ4, and HC. The cDNAs and genomic DNAs were used as templates for RT-PCR assay. For each of the first four predicted genes, no gene expression was detected in any of the five lines tested. By contrast, *RabGDIα* did show gene expression, but had no difference in expression levels between resistant and susceptible lines. *ZmGAPDH* was used as an internal control.

Reference: Dixit A , Ma K H , Yu J W , et al. Reverse transcriptase domain sequences from Mungbean (*Vigna radiata*) LTR retrotransposons: Sequence characterization and phylogenetic analysis[J]. Plant Cell Reports, 2006, 25(2):100-111

Functional validation:

Q2: Authors have generated number transgenic lines to demonstrate that RabGDIa is indeed the host factor that is required for RBSDV infection. However, some issues need to be resolved. Overexpression of GDIa even in the presence of GDIa-hel is sufficient to restore RBSDV infection but overexpression of GDIa-hel in the background of GDIa

has no effect on the infection. If this is due to titration effect, in the latter case titration does not work. Authors should provide some explanation. Confounded by these results is that RNAi of GDIa has no effect on susceptibility. A conclusive experiment would have been to delete GDIa-hel in resistance line or truncate GDIa to delete exon 10 in B73 susceptible line using CRISPR. This reviewer is aware that this is a major undertaking in maize.

A2: Thank you for your comments. In the transgenic functional validation, we made a total of six constructs for *ZmGDIα* and *ZmGDIα-hel* alleles, and demonstrated that the susceptible *ZmGDIα* allele is dominant over the resistant *ZmGDIα-hel* allele (Supplementary Figure 9-12). In the revised MS, we added the latest validation data conducted in this summer by using the complementation construct containing the native *ZmGDIα* gene. Of the $T_2F_1BC_2$ plants with the endogenous *ZmGDIα-hel* gene fixed, transgenic plants expressing the exogenous native gene *ZmGDIα* had significantly higher DSI values and RBSDV copy numbers than did non-transgenic plants (Fig. 2, also attached below). All these assessments clearly demonstrated that the wild-type *ZmGDIα* is a dominant susceptibility allele for RBSDV infection, while the natural *ZmGDIα-hel* variant is a recessive resistance allele.

All observed resistance performance is rooted in an interaction between the viral P7-1 effector and two ZmGDIα isoforms. P7-1 needs to recruit the host susceptibility factor ZmGDIα to initiate viral infection. Therefore, transgenic plants expressing ZmGDIα would finally lead to maize susceptibility to MRDD. In the RNAi transgenic plants, the presence of ZmGDIα, even at low level, is also sufficiently for viral infection. By contrast, P7-1 binds weakly to ZmGDIα-hel due to the alternative exon 10, leading to quantitative recessive resistance to MRDD. Since *ZmGDIα-hel* is recessive to *ZmGDIα* in maize resistance to MRDD, transgenic plants (with the endogenous *ZmGDIα* gene fixed) expressing *ZmGDIα-hel* could not enhance maize resistance to MRDD.

Since P7-1 binds both the wild-type exon 10 and C-terminal region, deletion of the exon 10 by CRISPR is not expected to completely block P7-1 binding to ZmGDIα. Such

mutant ZmGDIα is presumably to be another version of partial resistance gene, rather than a complete resistance gene. The other option may lie in the modification of some critical amino acids in the C-terminal region of ZmGDIα-hel to further weaken the interaction between P7-1 and ZmGDIα-hel. Only in this way would the modified *ZmGDIα-hel* gene show strong resistance to MRDD. Just as suggested, we did conduct experiments to disrupt *ZmGDIα* using CRISPR. We just got T_0 transgenic plants with a deletion of 1-46 bp in exon 10 (Table R2. attached below). However, it will take two more years to confirm their performance in resistance to MRDD.

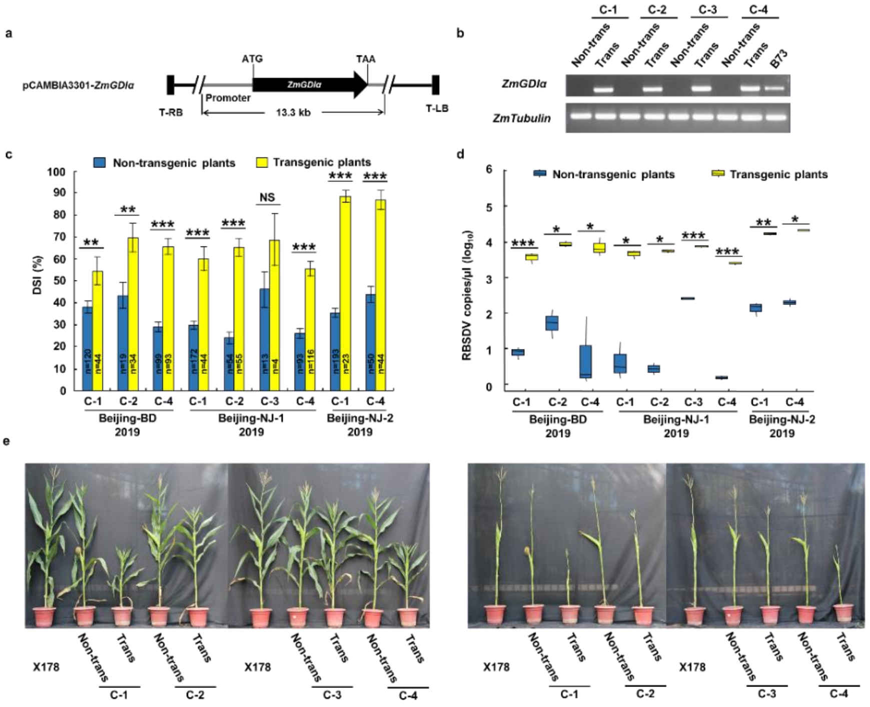

Figure 2 | Functional validation of *ZmGDIα* via a transgenic approach. Notably, transgenic plants expressing the exogenous native gene *ZmGDIα* had significantly higher DSI values (except for one transgenic event C-3) and RBSDV copy numbers than did non-transgenic plants. It is obvious that complementation of *ZmGDIα-hel* with the native *ZmGDIα* gene could largely restore the ability of RBSDV to systemically infect maize plants.

Table R2. The list of CRISPR T_0 transgenic plants

Code	No. of the deleted base pairs	The edited region	Position of thedeleted base pairs
1	10bp	exon10	*ZmGDIα*$^{7183\text{-}7192}$
2	7bp	exon10	*ZmGDIα*$^{7182\text{-}7194}$
3	1bp	exon10	*ZmGDIα*^{7183}
4	4bp	exon10	*ZmGDIα*$^{7013\text{-}7186}$
5	7/26bp	exon10	*ZmGDIα*$^{7174\text{-}7207/7178\text{-}7188}$
6	4bp	exon10	*ZmGDIα*$^{7182\text{-}7185}$
7	41/46bp	exon10	*ZmGDIα*$^{7178\text{-}7232/7178\text{-}7243}$

Molecular characterization:

Q3: Based on multiple assays presented in Fig. 4, it seems both GDIα and GDIα-hel interact with viral protein P7-1. Furthermore, GDIα can compete for interaction with P7-1 in the presence of GDIα-hel. How does this result translate in plants? In resistant plants only protein present is GDIα-hel. Since this can also interact with P7-1, how does this affect viral infection?

A3: That's a very good question. Plant viruses need to hijack host factors to finish their life cycles and cause viral diseases. Here, RBSDV initiates its infection of maize plants once the viral P7-1 effector binds tightly to the host susceptibility factor ZmGDIα. As a consequence, maize plants expressing *ZmGDIα* were all susceptible to MRDD, as observed in the homozygotes *ZmGDIα/ZmGDIα*, heterozygotes *ZmGDIα/ZmGDIα-hel*, transgenic plants expressing *ZmGDIα*, and RNAi transgenic plants. In resistant plants, however, P7-1 can only weakly bind to ZmGDIα-hel for inefficient infection, leading to quantitative recessive resistance to MRDD. Thus, the quantitative difference in binding of P7-1 by two protein isoforms finally leads to difference in maize resistance to MRDD. As suggested by the reviewer, we set up two experiments in vivo to investigate the competition of P7-1 interacting with two isoforms ZmGDIα/ZmGDIα-hel and try to address how these interactions translate in plants (please see the following A5).

Q4: In vivo co-IP experiment should include a negative control (Fig. 4c). Also band in a-Myc probed IP blot is barely visible. Why there is excess amount of GDIα-GFP in this blot?

A4: Thank you for your suggestion. We repeated the co-IP experiment by adding negative controls, and regulate the balance between ZmGDIα-GFP and ZmGDIα-hel-GFP. Consequently, the α-Myc probed IP bands could be clearly observed. Please refer to Fig. 4c that is also attached below. In our previous experiment, considering ZmGDIα-hel weakly binds to P7-1, the ZmGDIα-hel-EGFP and P7-1-Myc mixture co-immunoprecipitated with anti-GFP antibody was over loaded in order to make the α-Myc band visible.

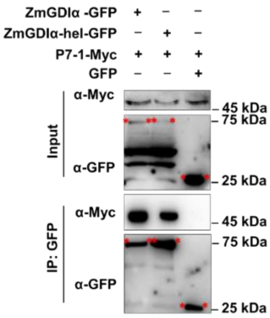

Figure 4c. Co-immunoprecipitation assay showing the interaction of the viral P7-1 with ZmGDIα or ZmGDIα-hel in vivo. P7-1-Myc and ZmGDIα-GFP (or ZmGDIα-hel-GFP) fusion proteins were co-expressed in *N. benthamiana* leaves. Total proteins were immunoprecipitated with anti-GFP magnetic beads. P7-1-Myc and ZmGDIα-GFP (or ZmGDIα-hel-GFP) were detected by immunoblotting with anti-Myc or anti-GFP antibody, respectively.

Q5: Similar issue also observed when GDIα(1-333) and GDIα-hel(1-318) are tested in luciferase complementation and in vitro pull down assays (Fig. 5). GDIα-hel(1-318) can be out competed when GDIα(1-333) is at higher concentration. Therefore, these results are not sufficient to make the conclusion "The results showed that P7-1 bound more tightly to ZmGDIα1–333 than to ZmGDIα-hel1–318 (Fig. 5f,g)" - lines 305-306. Furthermore, in resistant lines only protein present is ZmGDIα-hel; this can bind at similar strength to P7-1 as GDIα (see Fig. 4 and 5 in vitro pull-down data). Therefore, is binding alone is responsible for imparting resistance or susceptibility?

Authors could use transgenic lines expressing GDIα-EGFP and GDIα-hel-EGFP to see if there is different strength of P7-1 binding by MS analyses (similar to that they did to identify viral proteins that interact with GDIα - Suppl Fig. 12a and Suppl Table 3).

A5: This is a very interesting question. From the split luciferase complementation assay, we have noticed the difference in strength when P7-1 binds to ZmGDIα or ZmGDIα-hel (Fig. 4a). Similar result was also observed when P7-1 binds to ZmGDIα$^{1–333}$ or ZmGDIα-hel$^{1–318}$ (Fig. 5a,b). Then we used pull-down and in vivo co-IP to confirm interactions between P7-1 and ZmGDIα or ZmGDIα-hel (Fig. 4b,c), as well as between P7-1 and ZmGDIα$^{1–333}$ or ZmGDIα-hel$^{1–318}$ (Fig. 5e). As shown, we also found some differences did exist between two ZmGDIα isoforms in interacting with P7-1. Anyway, these assays are not enough to reveal the quantitative difference in binding strength. Because of this, we conducted the competition assays to measure the binding strength of P7-1 to ZmGDIα (or ZmGDIα$^{1–333}$) and ZmGDIα-hel (or ZmGDIα-hel$^{1–318}$). Taken ZmGDIα$^{1–333}$/ZmGDIα-hel$^{1–318}$ for example (Fig. 5f,g), ZmGDIα$^{1–333}$ could gradually replace ZmGDIα-hel$^{1–318}$ in interacting with P7-1 as ZmGDIα$^{1–333}$ content increased; however, ZmGDIα-hel$^{1–318}$ could hardly replace ZmGDIα$^{1–333}$ in the inverse assay. When both ZmGDIα$^{1–333}$ and ZmGDIα-hel$^{1–318}$ were set at the same high concentration of 200μg (the last column), the MBP-ZmGDIα$^{1-333}$ band is much strong than His-ZmGDIα-hel$^{1-318}$ (Fig. 5f,g). This is also true for ZmGDIα/ZmGDIα-hel (Fig. 4d,e;). Since ZmGDIα (ZmGDIα$^{1–333}$) and ZmGDIα-hel (ZmGDIα-hel$^{1–318}$) solely differ in exon 10, it is obvious that the different exon 10 variants underlie the quantitative difference in binding to the viral P7-1 protein.

In order to know exactly the difference in binding strength of P7-1 to two ZmGDIα isoforms in vivo, we here conducted another experiment. We mixed P7-1-nLUC with cLUC-ZmGDIα and cLUC-ZmGDIα-hel, separately, and the mixtures was individually filtrated into *N. benthamiana* leaves (Figure R 2a, attached below). The fluorescence signals were analyzed using ImageJ Launcher (National Institutes of Health) software to determine their intensities, so-called mean gray value (Figure R 2c, attached below). This assay was immediately followed by protein extraction and immunoblotting with

anti-LUC antibody, and again the immunoblot bands were measured by ImageJ Launcher (Figure R 2b, attached below). When comparing ZmGDIα with ZmGDIα-hel, the ratios of mean gray value for immunoblot bands are about twofold; however, the ratios of mean gray value for fluorescence signal intensity are over 12 times (Figure R 2d. attached below). This indicates that a higher proportion of ZmGDIα could bind to P7-1 as compared to ZmGDIα-hel in vivo, or in other words, the viral P7-1 protein binds more tightly to ZmGDIα than to ZmGDIα-hel in vivo.

We greatly appreciate the reviewer for the very constructive suggestion to use transgenic lines over-expressing ZmGDIα-EGFP and ZmGDIα-hel-EGFP to see if there is different strength of P7-1 binding to two ZmGDIα isoforms. As suggested, we inoculated the transgenic plants with RBSDV at the two-leaf stage and collected leaf tissues at 20 and 33dpi. Since both the *ZmGDIα-EGFP* and *ZmGDIα-hel-EGFP* exogenous genes were highly expressed as detected with qRT-PCR assay (Figure R3 a. attached below), then we used the anti-GFP antibody to immunoprecipitate both ZmGDIα-EGFP and ZmGDIα-hel-EGFP, followed by mass spectrometry analysis (Figure R3 b. attached below). No P7-1 protein could be immunoprecipitated with ZmGDIα-hel-EGFP at high stringency, and only tracible P7-1 peptides appeared when lowing stringency from high to medium. By contrast, abundant P7-1 proteins were immunoprecipitated with ZmGDIα-EGFP (Figure R3 b, attached follow). Since the transgenic plants have endogenous *ZmGDIα* genes and are susceptible to RBSDV infection, they must have sufficient P7-1 proteins in the infected cells. This IP-MS assay clearly demonstrates that P7-1 is more likely to bind to ZmGDIα than ZmGDIα-hel in vivo. Moreover, the tight binding of P7-1 to ZmGDIα is a prerequisite for the successful viral infection of maize plants. Thus, the difference in binding strength of P7-1 to two ZmGDIα isoforms is assumed to be the basis for imparting resistance or susceptibility to MRDD. Anyway, more works are required to illustrate the molecular mechanism, which are underway.

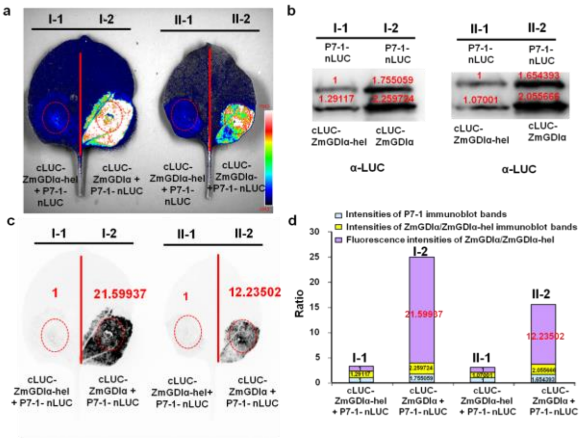

Figure R2: Detection of binding strength of P7-1 to two ZmGDIα isoforms in vivo. The split-luciferase complementation assay was conducted in duplicates. Here, I and II means replicate I and II; while 1 and 2 correspond to ZmGDIα-hel and ZmGDIα, respectively. **a.** Split-luciferase complementation assay to detect the interactions of viral P7-1 with ZmGDIα-hel and ZmGDIα. When co-expressed with P7-1-nLUC in the same *N. benthamiana* leaf, cLUC-ZmGDIα showed much strong fluorescence signals than cLUC-ZmGDIα-hel. **b.** Quantitative detection of transiently expressed proteins. The protein abundance of P7-1, ZmGDIα and ZmGDIα-hel were detected using immunoblotting with anti-Luciferase, and the mean gray values were recorded and calculated by ImageJ. **c.** Quantitative detection of fluorescence signal intensity. The mean gray values of fluorescence intensities were recorded and calculated by ImageJ. **d.** The ratios of mean gray values of fluorescence signal intensity, protein abundance of P7-1, ZmGDIα/ZmGDIα-hel. The abundance ratios of ZmGDIα and ZmGDIα-hel are less than twofold; while the fluorescence intensity ratios of ZmGDIα and ZmGDIα-hel are more than 12 times.

a

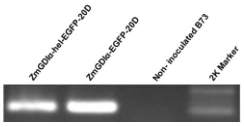

b

Material	Sampling times of inoculation with RBSDV (dpi)	The ZmGDIα-hel-EGFP / ZmGDIα-EGFP ratio of score and area	Viral protein of P7-1	Peptide confidence	Sequence	PSMs	Score
Over-exppressiing the *ZmGDIα-hel-EGFP* fusion gene transgenic plants	20	1	none	High	none	none	none
			Yes	Medium	NLVYGGNYqLVYqALFYYYIVTnGR	2	0
					MVTSLSLGnAFISEGRYHYAIDTTEqTSAE DADALRFLAR	1	
					TDLVAAQQTLIYYYFGNSYqGIHLNWDSK	3	
	33	1.659233349	none	High	none	none	none
Over-exppressiing the *ZmGDIα-EGFP* fusion gene transgenic plants	20	0.696667322	Yes	High	QLEVENPLNNTcLITDNGLRSEVPINANSS STAAPTELPVFSSLS	1	0
				Medium	ALcVNILR	1	1.65
					ANTKnEIR	2	
					FSKANTKnEIR	1	
					FSKANTKNEIREMR	1	
					GLRSKnLVYGGNYQLVYQALFYYYIVTNG RFSSGFNVR	2	
					IAKIVIKNDVEKTDLVAAqqTLIYYYFGNSY QGIHLnWDSK	1	
					IPDVPLKMYGDLHFHEqFTNDVDLDLLcW qLLSSNqDSRALcVNILRmVTSLSLGNAFI SEGR	1	
					IVIKNDVEK	1	
					mVTSLSLGnAFISEGR	1	
					NEIREmRIYK	2	
					YLRqLEVENPLnnTcLITDnGLR	4	
					YLRQLEVENPLnnTcLITDnGLR	2	
	33	0.825363083	Yes	High	QLEVENPLNnTcLITDnGLRSEVPInANSSS TAAPTELPVFSSPSA	5	8.8755
					QLEVENPLNNTcLITDNGLRSEVPINAnSS STAAPTELPVFSSPSA	7	

Figure R3. IP-MS assay to detect binding abilities of P7-1 to ZmGDIα-EGFP and ZmGDIα-hel-EGFP. a. qRT-PCR assay of the RBSDV capsid protein RS-10. The bright qRT-PCR bands indicate the successful viral infection of transgenic lines over-expressing *ZmGDIα-EGFP* and *ZmGDIα-hel-EGFP*. b. Detection of P7-1 protein which were immunoprecipitated with ZmGDIα-EGFP or ZmGDIα-hel-EGFP at 22 and 33dpi.

Reviewer #3 (Remarks to the Author):

Q1: Liu et al. report a great deal of work fine-mapping and showing the single gene that seems to be the sole contributor to recessive quantitative resistance to maize rough dwarf disease in maize. The authors also show that the resistant allele at this locus has an insertion that interferes with interaction with the RBSDV P7-1 protein, and propose a model for why this interaction may be important during virus infection. The conclusions and extensive screening of maize germplasm for alleles are of great interest to maize virologists and breeders, as MRDD is an important viral disease. Despite the volume of work shown here, much work remains to be done to elucidate the mechanism by which this interaction confers resistance. I also question how impactful this work will be in the end, since the identified resistance is both recessive (and most commercial maize is hybrid, requiring both inbred parents to have the recessive allele), and quantitative and therefore does not eliminate infection or inoculum source from plants. Thus while it represents a major advance in cloning the gene and identifying a virus interaction that appears to be critical (and I fully appreciate that doing reciprocal mutagenesis on the virus side is not within reach for this virus), I am on the fence about how impactful this discovery his for the intended audience here. (In my opinion, cloning of broadly used potyvirus resistance in maize and beginning to understand its mechanism of action is higher impact as resistance is dominant and nearly complete).

A1: We greatly appreciate the reviewer's positive comments on our work. We fully agree that much work remains to be done to elucidate the molecular mechanism underlying viral infection and maize resistance. Dr. Suining Deng, a co-first author of the article, continues this work, trying to address the following questions. How an interaction between the viral P7-1 effector and host susceptibility ZmGDIα initiates viral infection and causes MRDD syndrome, like stunted plant, shortened internodes, malformed tassels/ears, and among others? How a weak interaction between P7-1 and ZmGDIα-hel interferes with viral propagation and dissemination to alleviate MRDD symptoms?

We are very understandable the reviewer's concerns on the application of the resistance gene *ZmGDIα-hel*. As a recessive resistance gene, *ZmGDIα-hel* should be introduced into both parental lines to breed high-resistant maize hybrids. Since the naturally-occurring resistance alleles have the same origin and show no any sequence variation, we need to carefully pick up different sources of donors to separately introduce into parental lines via marker-assisted selection. Moreover, we need to minimize the overlapped *ZmGDIα-hel* region to reduce the potential negative impacts of homozygous chromosomal segments on heterosis. In addition, we can create an artificial high resistance gene by editing some critical amino acids in the C-terminus of ZmGDIα-hel using CRISPR, and this modified *ZmGDIα-hel* gene may completely block the binding of P7-1 to ZmGDIα-hel to confer full resistance to MRDD.

We agree that a major dominant resistance gene, like potyvirus resistance gene, may have higher impacts on disease control. However, we did not find such a dominant and complete resistance gene against *Fijivirus* in maize germplasm. *ZmGDIα-hel* at the major QTL *qMrdd1* was proved to be the causal gene in resistance to MRDD and has been repeatedly detected by several groups. The findings suggest the great value of natural *ZmGDIα-hel* in development of resistant varieties. For instance, the hybrid 'Shannong 206' developed by the co-first author, Prof. Baosheng Liu is currently the strongest MRDD-resistant corn hybrid in China.

Q2: The manuscript shows carefully conducted work that appears to be very thorough in presentation and meticulous in completion, so I did not uncover any errors creating scientific qualms about the reported findings. Consistency in nomenclature (including defining term usage early) and streamlining the manuscript would greatly improve its accessibility. For example, the authors seem undecided about how to refer to the virus and disease, with "maize rough dwarf virus disease" in the title, "Maize rough dwarf viral disease" in the first line, and variations on this throughout. The correct nomenclature is "maize rough dwarf disease", MRDD. Terms such as passive resistance should be explained at the beginning rather than at the end, along with

crucial background information about the nature of resistance and the extent of quantitative contribution that are described in detail only at the end in the current manuscript. Wording errors such as L43 “threatening world food safety” should be corrected (security?), and the writing can certainly be streamlined.

A2: We greatly appreciate the reviewer’s comments on the manuscript. In the revised MS, we consistently use the correct nomenclature for “maize rough dwarf disease” and others. As suggested, we move some parts, like passive resistance, from ‘discussion’ to ‘introduction’ sections. Please refer to the revised MS. Additionally, we accepted the reviewer’s suggestion to substitute ‘safety’ with ‘security’. Please refer to Line 45.

Q3: Finally, the number of supplementary figures and tables is staggering, even for a Nature contribution, and some streamlining and/or consolidation would assist in orienting readers. Some detailed suggestions on Figures and Table consolidation and incorporation of key data into the main figures rather than supplemental and vice versa are outlined below:

A3: Thank you! We agree that large amounts of data are included in the supplementary data. The project has been carried out for more than 10 years to create lots of data which are interconnected to unveil a clear picture how maize plants show resistance to MRDD. Thus, we prefer to keep them. Meanwhile, we revise the manuscript/figure legends and make the them more readable. We’ll address all that you are concerned in follow.

Q4: Fig. 1. Fine-mapping the MRDD resistance QTL qMrdd1. Unclear from legend. Are stunted plants infected with RBSDV?

A4: Thank you very much for the remainder. Yes, all plants are inoculated with RBSDV. We rewritten the legend to give a clear description of Fig. 1a, b. Please refer to the legend of Fig. 1 (Lines 883-884 in the revised MS).

Q5: Should show in fig. 1 the susceptible and resistant parents if they exist. What population/plants are a. and b. figures from?

A5: We thank the reviewer's suggestion. Based on our previous mapped region of 1.2Mb, we continued to fine-map *qMrdd1* with two mapping populations. Given that maize resistance to MRDD is a typical quantitative trait, we adopted a scale of five grades to measure disease severities for plants infected with MRDD. So, we don't think it is very necessary to show four parental lines in Fig. 1. In the transgenic validation, we did use the infected susceptible (B73) and resistant (X178) lines as controls. The susceptible (HuangC) and resistant (X178) lines shown in the bottom of Fig. 6. Since the comments is just suggestive, we have kept it in though we would like to add parents if the reviewer or editor felt strongly otherwise. In addition, we added one sentence to briefly described the origins of P1 and P2 populations. Please refer to figure legend, Lines 889-891.

Q6: Fig. 2. Define DSI in legend. Transgenic plants for ZmGDIαlpha-EGFP have increased DSI percent? E. RBSDV copies: is there an absolute quantification of copy number?

A6: In the revised MS, we added the latest validation data with the native *ZmGDIα* gene as Fig. 2, and move the original Fig. 2 to supplementary data as Supplementary Figure 9. As suggested, we added the definition of DSI in the legends for both figures, please refer to Fig. 2 lines 906-907 and Supplementary Figure 9. Yes! Transgenic plants overexpressing *ZmGDIα-EGFP* could increase DSI value in the *ZmGDIα-hel* genetic backgrounds, indicating that complementation of *ZmGDIα-hel* with *ZmGDIα* could largely restore the ability of RBSDV to infect maize plants.

The copy number of RBSDV is an absolute quantification. We established standard curve of copy numbers of *RBSDV-S10* and concentration of the cDNA of the maize *tubulin* gene *ZmTubulin* to directly quantify copy numbers of RBSDV. Please refer to the supplementary methods "Quantitative measurement of RBSDV".

Q7: Fig. 3. Expression profiles of ZmGDIα and ZmGDAIα-hel. Shows no change in transcript levels between the two with or without inoculation.

*This could be supplemental data

A7: Thank you for the suggestion. So far, we wouldn't like to put Fig. 3 in the supplemental data. Based on the facts that both *ZmGDIα* and *ZmGDIα-hel* showed no difference in transcript levels between control and inoculation and under different developmental stages, we infer it is the protein structure, but not the gene expression level, that differs resistance from susceptible alleles. This is a key point of this study. If the reviewer or editor feel strongly it should be moved to supplementary data we would like to.

Q8: Fig. 4. P7-1 binding to GDI and GDIα-hel. In a., why is first photo showing bleaching out? Meaning of white is not shown in high-low reference bar.

A8: I'm sorry the picture confused you. The white with red colour in the first image indicated the strong interaction between P7-1-nLUC and cLUC-ZmGDIα, as shown on the top of the high-low reference bar. We marked the borders to the reference bar to make it clear for readers. Please refer to the revised Fig. 4.

Q9: Fig. 5. Targeting regions or interacting regions?

A9: Thank you for your mention. These two terms look similar, but still have a subtle difference in the meanings. We carefully check the whole manuscript to use the correct term where it is suitable for.

Q10: Fig. 6. Model

A10: We modified the title. Please refer to modified figure legend, line 983 in the revised version.

Supplemental figures and tables:

Q11: Even for a Nature article, the number of supplemental figures and tables in addition to the main text figures is excessive. Needs focus on the most salient information.

Fig S1. Inaccurate title. Not really cytogenetics: this is cellular ultrastructure. I think that showing the difference between NILs should be in the main manuscript, not supplemental data.

A11: Since the manuscript covers a lot of works, from fine-mapping to application of resistance gene *ZmGDIα-hel*, it looks a bit excessive. We just want to tell the readers a complete story of *ZmGDIα-hel*-mediated quantitative resistance to MRDD and prefer to keep all these figures and tables. If the reviewer or editor think the data is excessive, we may chop some down. As suggested, we give a new title of Supplementary Figure 1 as “Cellular ultrastructure of MRDD symptoms” and modify the corresponding part in the text. Because the main text already includes 6 figures, we think maybe it is too much to include Supplementary Figure 1. Surely, if the reviewer or editor strongly think it is necessary, we would like to do so.

Q12: Fig. S2. Fine mapping qMrdd1

A12: Yes! It takes a long time to narrow *qMrdd1* down to a few candidate genes. In order to accelerate the pace of fine-mapping, we simultaneously used two mapping populations. Supplementary Figure 2 describes the sequential fine-mapping with the population 1.

Q13: Fig. S3. More fine mapping: can it be combined with Fig. S2?

A13: Supplementary Figure 3 showed the fine-mapping process with the population 2. Since two populations did not show the exact same fine-mapping progress, fine-mapped region, and molecular markers, it is very difficult to combine them into one figure.

Q14: Fig. S4. Should probably be in main text rather than the current Fig. 3.

A14: In Fig. 1, we summarized the data from Supplementary Figure 2, 3, and 4. Supplementary Figure 4 is critical to reveal candidate genes in the mapped region, but we don’t think all data need to be presented in the main text. By contrast, we think Fig. 3 is more informative to characterize both *ZmGDIα* and *ZmGDIα-hel* and their relationship with viral propagation. Since the comments is just suggestive, we have kept it in though we would like to do so if the reviewer or editor felt strongly otherwise.

Q15: Fig. S5. Alignment of maize alleles

A15: As suggested, we modify the title. We just want to give readers the sequence info as easy as possible. The sequence dada has been deposited in the GenBank website (MK412542-MK412566, MK420471- MK420493).

Q16: Fig. S7. Alignment of protein orthologs from various plants

A16: Accepted and modified!

Q17: Fig. S8. Phylogenetic analysis. Can this be included in a panel in a consolidated figure (e.g. with gene structure from Fig. S4 and summarized expression data)? Show branch lengths as well as branch support.

A17: Thank you for your suggestion. We have modified Supplementary Figure 8 and added branch lengths and branch support. In Supplementary Figure 4, we summarize the discovery of candidate genes, which is apparently different from phylogenetic analysis in Supplementary Figure 8, so we would like to keep Supplementary Figure 8 as it stands.

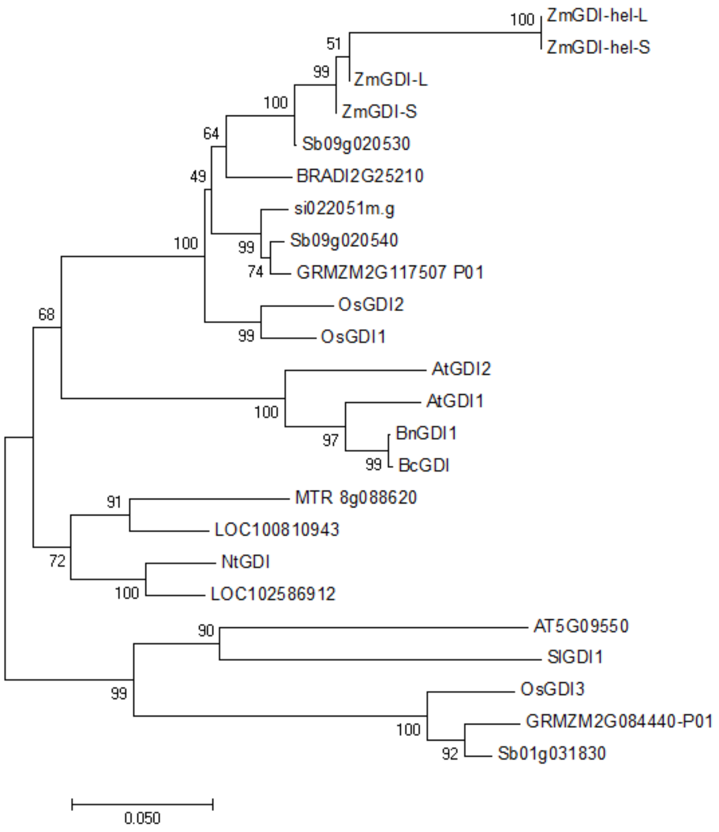

Supplementary Figure 8. Phylogenetic analysis of GDI proteins from various plant species. GDI proteins were retrieved from *Arabidopsis thaliana* (At), *Zea mays* (Zm), *Oryza sativa* (Os), and *Sorghum bicolor* (Sb), and others. A phylogenetic tree was constructed using the maximum-likelihood method based on the JTT+G matrix-based model in MEGA 6.0. ZmGDIα-L and ZmGDIα-hel-L proteins are deduced from the long transcripts of inbred lines HuangC (susceptible) and X178 (resistant), respectively, while ZmGDIα-S and ZmGDIα-hel-S proteins are deduced from the short transcripts of inbred lines HuangC (susceptible) and X178 (resistant), respectively.

Q18: Fig. S9. Transgenic validation. Panel d. does not add much.

A18: When compared full-cDNA with genomic DNA, we found two transcripts for both *ZmGDIα* and *ZmGDIα-hel* alleles. So, it is necessary to investigate both transcripts in maize resistance to MRDD. In the revised MS, the short *ZmGDIα* transcript was proved to complement *ZmGDIα-hel* to restore maize susceptibility to MRDD (Supplementary Figure 9). The long *ZmGDIα* transcript is obviously unable to restore the ability of RBSDV to infect maize plants (Supplementary Figure 10d). This result serves as additional information to corroborate the true function of the short transcript of *ZmGDIα.*

Q19: Fig. S10. Transgenic functional analysis of ZmGDIα-hel. The DSI panels do not add much—could be summarized more simply.

A19: Over-expression of both short and long transcripts of ZmGDIα-hel could not enhance maize resistance to MRDD in a susceptible B73 backgrounds. This confirms that *ZmGDIα-hel* acts as a recessive resistance gene. We agree that DSI panels may not add much, but we want to keep it, as this also demonstrates that ZmGDIα could complement ZmGDIα-hel in the host susceptibility to MRDD.

Q20: Fig. S11. RNA9-mediated functional assay. Again, DSI panels do not add much.

A20: RNAi-mediated reduction of ZmGDIα has no impact on disease resistance in transgenic plants, indicating the presence of ZmGDIα is indispensable for RBSDV to invade maize. So, no difference in DSI between transgenic and non-transgenic plants also pointed to ZmGDIα as a critical factor to viral infection.

Q21: Fig. S12. Testing P7-1, P8, P10 for interaction with plant protein

A21: Yes! we modify Supplementary Figure 12 by adding the split-luciferase complementation assay of P7-1. Please refer to the modified Supplementary Figure 13 in the revised MS.

Q22: Fig. S13. Alignment and predicted structures of alleles differing in susceptibility

A22: Accepted and modified!

Q23: Fig. S14. Haplotypes

A23: Modified!

Q24: Fig. S15. Evolution prediction for resistant allele

A24: We modify the title as ‘The nucleotide diversities and minimum-spanning tree at the *ZmGDIα* locus’.

Q25: Fig. S16. Tests of other natural alleles

A25: Yes! We prefer to keep it!

Q26: Fig. S17. Breeding comparison. Could include in main text, perhaps combined with model figure (Fig. 6).

A26: We would like to keep it as supplementary data since application of *ZmGDIα-hel* in breeding of resistant lines is not the main issue in the manuscript. If the reviewer or editor felt strongly to combine it with Fig. 6, we would like to do so.

Q27: Table S1. Could be included with DSI description

A27: Thank you for your comment. We delete the Table S1, and meanwhile give a detailed description of DSI in the M&M section, please refer to the Assessment of MRDD symptoms in the field, lines 506-514 in the revised MS.

Q28: Table S2. Primers

A28: We modified the title as ‘The list of primers used in the research’, please refer to Supplementary Data 1.

Q29: Table S3. Can data shown in Fig. S12 be combined into table?

A29: Thank you for your comment. The viral proteins which were immunoprecipitated were

summarized in Supplementary Data 2. In Supplementary Figure 13, we adopted the pull-down and split-luciferase complementation assays to test which one of three viral proteins interacted with ZmGDIα. We try to put table and figure together, however, it looks ugly. Since the comments is just suggestive, we have kept it in though we would like to combine them if the reviewer or editor felt strongly otherwise.

Q30: Table S4. Again, duplicates some information show in Fig. S14. Suggest choosing one. “Unkown” typo throughout.

A30: Thank you for your suggestion. We listed all 620 maize inbred lines in Supplementary Data 3 and these data may be helpful for corn breeders or readers. We correct ‘unkown’ typo throughout the Supplementary Data 3. In comparison of various *ZmGDIα* alleles, we found 20 haplotypes at the *ZmGDIα* loci as listed in Supplementary Figure 15. Considering from the perspective of application, we think these data may be valuable to corn breeders.

Q31: Table S5. Landraces. Can be consolidated with Table S4?

A31: Thank you for your suggestion. We have combined them into Supplementary Data 3.

Q32: Table S6. Tseosinte panel tested for insertion.

A32: We have combined Tables S4, S5, and S6 into one Supplementary Data 3.

Q33: Table S7. KHA test inputs.

A33: The first six inputs in Supplementary Data 4 (AY104395, AY106816, AY107192, AY107248, AY111546 and AY111711) were used for neutral loci control (Studer A, et al. 2011, Nature Genetics), *ZmGDIα/ZmGDIα-hel* sequences of 49 individuals were performed using DnaSP v5.1 to obtain inputs of *ZmGDIα.*

Q34: Table S8. MRDD resistance inbreds.

A34: Sorry! The title may cause you confused. A panel of 186 inbred lines were evaluated for their resistance to MRDD in the field tests for three years. The results are summarized in the Supplementary Data 5.

REVIEWERS' COMMENTS:

Reviewer #1 (Remarks to the Author):

The authors have addressed all my concerns on the original submission.
Beat Keller

Reviewer #2 (Remarks to the Author):

Authors have addressed most of the issues raised during last review and the manuscript has significantly improved.

Reviewer #3 (Remarks to the Author):

The manuscript is greatly improved by the first set of revisions. Some residual issues in the writing remain.

L32-33: clarify sentence
L36: change "difficulty to be recruited" to "difficulty being recruited"
L41: engineer RBSDV resistance?
L46: to complete their infection cycles
L47: delete 'against'
L48-49: use consistent tense (this is past tense, previous sentence is present)
L62: major maize-growing areas in China
L71: fijivirus should not be capitalized
L75-76: P7-1 is a tubule-forming protein in both infected plants an insect vectors
L78: movement function demonstrated or putative? No infectious clones.
L89: and a number of (give number) quantitative trait loci. . .
L99-100: P7-1 protein is the pathogenicity determinant that targets ZmGDIα. . .

Fig. 1, Fig. S1. Indicate the virus (not just disease) in the legend. Also indicate plant cultivar/genotype(s) used in legend and dpi where missing.

L108, Fig. S1: Did any of the other internodes show differences in cell elongation, or were they looked at?

L132-133: use past tense for consistency

Fig. S4c: Is this semi-quantitative PCR? State also in the text. How much difference would you have been able to detect with the methodology used?

L203: four independent transgenic events were obtained, with gene expression observed. . .
L224-226: fix sentence grammar
L236-237: ZmGDIα-hel resistance is recessive.

L256: RT-qPCR
L264: amino acid
L269: recruits RabGDIα during viral infection (whether this is at infection initiation or later is not demonstrable from these data)
L280: delete "consequently"
L284: assays
L287: using an in vivo co-immunoprecipitation assay.

L288: protein binding competition assay

L266: “binds” is much more clear than “targets” as used in the revised section headings. Please make the shift from “target” to “bind” consistently throughout manuscript.
L290: ZmGDIα-hel was much less effective at replacing (OR displacing) ZmGDIα in the inverse assay
L291: binds
L312: did not show any interaction with P7-1 in assays, . . .
L356: indicate more information about field tests here. Were plants inoculated or naturally infected? What is meant by “severity”? Do you mean incidence of infection in susceptible controls, or actual disease symptom severity with comparable incidences?

L387: Are there any ZmGDIα loss of function mutants? Are there known detrimental effects in maize if the encoding gene is lost or disrupted?

L392: RabGDIα inhibits host defense
L392-396: indicate host species in the example cases
L397-398: It seemed conceivable that . . .
L402: and the C-terminal 67 aa of RabGDIα in maize.
L403: shows (present tense for previously well-established facts). . . S7-1 shows high (is it highest? One of the top x highest—be precise) expression. . .
L412: Movement is not initiation of viral infection—it is movement of infection. The mechanism proposed with thus be an inhibition of virus cell-to-cell and/or systemic movement, limiting virus to subliminal or local infections. This is at least a partially testable model: if RBSDV still replicates in cells (protoplasts homozygous for ZmGDIα-hel), it is not an infection incompatibility in those cells, it may just be a movement restriction.

L432: yes, but it could also be cell-to-cell (not just systemic) movement that is slowed.
L433: delete ‘massive’
Do you have any evidence to suggest that systemic movement is disrupted or slowed for ZmGDIα-hel plants?

L436: There are other examples of potyvirus movement restriction by dominant genes, including RTM resistance to TEV in Arabidopsis and maize potyvirus restriction by Scm1/Wsm1
L476: Some previous wording was better. “Since maize diseases are prevalent in tropical areas, tropical maize may be more likely to gain and retain. . .”

L556: complementation

L616: equal amounts of OD600-normalized cultures?

RSPONSE TO REVIEWERS' COMMENTS

Reviewer #1 (Remarks to the Author):

The authors have addressed all my concerns on the original submission.

Beat Keller

Response: We greatly appreciate Prof. Beat Keller's valuable comments and contributions to our manuscript.

Reviewer #2 (Remarks to the Author):

Authors have addressed most of the issues raised during last review and the manuscript has significantly improved.

Response: We greatly appreciate your valuable comments and contributions to our manuscript.

Reviewer #3 (Remarks to the Author):

The manuscript is greatly improved by the first set of revisions. Some residual issues in the writing remain.

Response: We greatly appreciate your valuable comments and contributions to our manuscript. We will give point-by-point response to your comments in the followings.

Q1: L32-33: clarify sentence

A1: The sentence has been modified as 'The viral P7-1 protein binds tightly to the exon-10 and C-terminal regions of RabGDIα to recruit it for viral infection'. Please refer to lines 31-32.

Q2: L36: change "difficulty to be recruited" to "difficulty being recruited"

A2: Accepted and changed! Please refer to lines 35.

Q3: L41: engineer RBSDV resistance?

A3: Thank you for your suggestion. Yes! the discovery of resistance gene here could be used to engineer resistance to RBSDV in other crops. As suggested, we added 'resistance' after RBSDV. Please refer to lines 39.

Q4: L46: to complete their infection cycles

A4: Accepted and changed! Please refer to lines 44.

Q5: L47: delete 'against'

A5: Accepted and deleted! Please refer to lines 45.

Q6: L48-49: use consistent tense (this is past tense, previous sentence is present)

A6: Accepted and modified! Please refer to lines 46-47.

Q7: L62: major maize-growing areas in China

A7: Thank you for the reminder, we delete 's' for 'valleys'. Please refer to lines 58.

Q8: L71: fijivirus should not be capitalized

A8: Accepted and changed throughout the manuscript! Please refer to lines 27, 61, 67, 70, and 466.

Q9: L75-76: P7-1 is a tubule-forming protein in both infected plants an insect vector.

A9: Accepted and changed! Please refer to lines 72.

Q10: L78: movement function demonstrated or putative? No infectious clones.

A10: Since no concrete evidence has been demonstrated concerning the viral P7-1 protein in movement function, we here add 'presumably' before 'to assist'. Please refer to lines 74.

Q11: L89: and a number of (give number) quantitative trait loci.

A11: Accepted and changed! Please refer to lines 85.

Q12: L99-100: P7-1 protein is the pathogenicity determinant that targets ZmGDI□. . .

A12: Accepted and changed! We also replace ‘targets’ with ‘binds’. Please refer to lines 97.

Q13: Fig. 1, Fig. S1. Indicate the virus (not just disease) in the legend. Also indicate plant cultivar/genotype(s) used in legend and dpi where missing.

A13: Accepted and modified! Please refer to the legends of Fig. 1 and Supplementary Figure 1.

Q14: L108, Fig. S1: Did any of the other internodes show differences in cell elongation, or were they looked at?

A14: Yes, other internodes also display differences in cell elongation. Normally, the upper internodes have stronger suppression of cell elongation than the lower internodes in the diseased maize plant. We here just present the ninth internode as an example to illustrate the difference of cell elongation between resistant and susceptible NILs.

Q15: L132-133: use past tense for consistency

A15: Accepted and changed! Please refer to lines 130.

Q16: Fig. S4c: Is this semi-quantitative PCR? State also in the text. How much difference would you have been able to detect with the methodology used?

A16: Supplementary Figure 4c shows the PCR products of RACE (Rapid Amplification of cDNA ends). The method was described in the M&M, ‘Construction of full-length cDNAs of ZmGDIα-hel and ZmGDIα’ section.

Q17: L203: four independent transgenic events were obtained, with gene expression observed. . .

A17: Accepted and modified! We modified the sentence as ‘Four independent transgenic events were obtained, expressing the exogenous ZmGDIα gene’. Please refer to lines 199-201.

Q18: L224-226: fix sentence grammar

A18: Accepted and changed! Please refer to lines 215-216.

Q19: L236-237: ZmGDI□-hel resistance is recessive.

A19: Accepted and changed! Please refer to lines 226-227.

Q20: L256: RT-qPCR

A20: Accepted and changed! Please refer to lines 246.

Q21: L264: amino acid

A21: Accepted and changed! Please refer to lines 254.

Q22: L269: recruits RabGDI□ during viral infection (whether this is at infection initiation or later is not demonstrable from these data)

A22: Accepted! P7-1 could recruit RabGDIα during viral infection. Please refer to lines 258.

Q23: L280: delete “consequently”

A23: Accepted and deleted! Please refer to lines 269.

Q24: L284: assays

A24: Accepted and changed! Please refer to lines 272.

Q25: L287: using an in vivo co-immunoprecipitation assay.

A25: Accepted and changed! Please refer to lines 275.

Q26: L288: protein binding competition assay

A26: Accepted and modified as competitive protein-binding assay! Please refer to lines 277-278.

Q27: L266: “binds” is much more clear than “targets” as used in the revised section headings. Please make the shift from “target” to “bind” consistently throughout manuscript.

A27: Accepted! We made the shift from “target” to “bind” throughout manuscript!

Q28: L290: ZmGDI□-hel was much less effective at replacing (OR displacing) ZmGDI□ in the inverse assay

A28: Accepted and changed! Please refer to lines 280.

Q29: L291: binds

A29: Accepted and changed! Please refer to lines 281.

Q30: L312: did not show any interaction with P7-1 in assays, . . .

A30: Accepted and changed! Please refer to lines 304.

Q31: L356: indicate more information about field tests here. Were plants inoculated or naturally infected? What is meant by “severity”? Do you mean incidence of infection in susceptible controls, or actual disease symptom severity with comparable incidences?

A31: Plants were naturally infected. The “severity” means incidence rate of MRDD across different field trials. We modified the sentence for clarity. Please refer to lines 347-349.

Q32: L387: Are there any ZmGDI□ loss of function mutants? Are there known detrimental effects in maize if the encoding gene is lost or disrupted?

A32: So far, we have not found the loss-of-function mutants and thus have no idea what kinds of detrimental effects will be in the case of the lost or disrupted RabGDIα gene.

Q33: L392: RabGDI□ inhibits host defense

A33: Accepted and changed! Please refer to lines 373.

Q34: L392-396: indicate host species in the example cases

A34: Accepted! We add the host species in the example cases. Please refer to lines 376-377.

Q35: L397-398: It seemed conceivable that . . .

A35: Accepted and changed! Please refer to lines 379.

Q36: L402: and the C-terminal 67 aa of RabGDI in maize.

A36: Accepted and changed! Please refer to lines 383-384.

Q37: L403: shows (present tense for previously well-established facts). . . S7-1 shows high (is it highest? One of the top x highest—be precise) expression. . .

A37: As suggested, we modified the sentence. Please refer to lines 384-385.

Q38: L412: Movement is not initiation of viral infection—it is movement of infection. The mechanism proposed with thus be an inhibition of virus cell-to-cell and/or systemic movement, limiting virus to subliminal or local infections. This is at least a partially testable model: if RBSDV still replicates in cells (protoplasts homozygous for ZmGDI-hel), it is not an infection incompatibility in those cells, it may just be a movement restriction.

A38: Thank you for the comment. We agree that P7-1 must play a key role in viral movement within the susceptible maize; while RabGDIα-hel presumably functions to restrict viral movement, limiting virus to local infections. We have changed "to initiate viral infection" to "for successful viral infection" in line 394, and made similar modifications throughout the manuscript to clarify this.

Q39: L432: yes, but it could also be cell-to-cell (not just systemic) movement that is slowed.

A39: Yes! Due to low efficiency in assembly of the P7-1/RabGDIα-hel trafficking complex, RabGDIα-hel could slow viral cell-to-cell movement. We have changed "and thus

interferes with viral systemic infection of a plant” to “and thus limiting the viral movement within a plant”.

Q40: L433: delete ‘massive’. Do you have any evidence to suggest that systemic movement is disrupted or slowed for ZmGDI-hel plants?

A40: As suggested, we deleted “massive”. Since we don’t have conclusive evidence supporting that systemic movement is disrupted or slowed for *RabGDIα-hel* plants, we have changed “systemic infection” to “viral movement with a plant” for clarity. Please refer to lines 415-416.

Q41: L436: There are other examples of potyvirus movement restriction by dominant genes, including RTM resistance to TEV in Arabidopsis and maize potyvirus restriction by Scm1/Wsm1

A41: Yes! Since *RabGDIα-hel* gene is the recessive resistance, we did not mention dominant gene-mediated restriction of viral movement.

Q42: L476: Some previous wording was better. “Since maize diseases are prevalent in tropical areas, tropical maize may be more likely to gain and retain. . .”

A42: Accepted and modified! Please refer to lines 457-458.

Q43: L556: complementation

A43: corrected! Please refer to lines 543-544.

Q44: L616: equal amounts of OD600-normalized cultures?

A44: added! Please refer to lines 603.
